# Changing the Reaction Pathway of Silyl-Prins Cyclization by Switching the Lewis Acid: Application to the Synthesis of an Antinociceptive Compound

**DOI:** 10.1021/acs.joc.3c00050

**Published:** 2023-05-23

**Authors:** Carlos Díez-Poza, Laura Fernández-Peña, Paula González-Andrés, Asunción Barbero

**Affiliations:** Department of Organic Chemistry, Faculty of Sciences, University of Valladolid Faculty of Sciences, Campus Miguel Delibes, Paseo de Belén 7, 47011 Valladolid, Spain

## Abstract

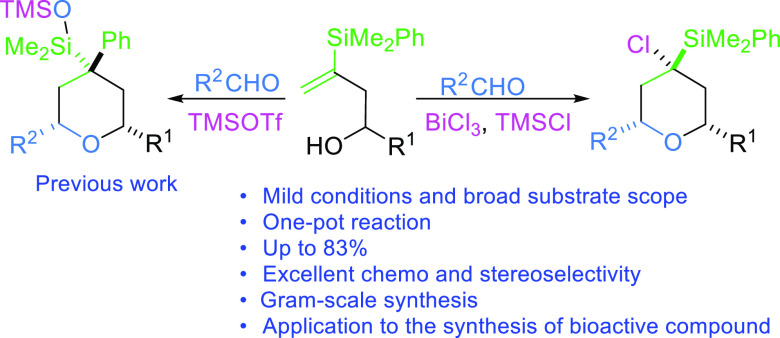

Developing new procedures for the synthesis of tetrahydropyrans in a very stereoselective manner is of great importance for the synthesis of THP-containing natural products. Here, we report an interesting protocol for the synthesis of polysubstituted halogenated tetrahydropyrans by silyl-Prins cyclization of vinylsilyl alcohols, in which the nature of the Lewis acid determines the outcome of the process. The methodology has been applied to the synthesis of a known antinociceptive.

## Introduction

**Figure 1:**
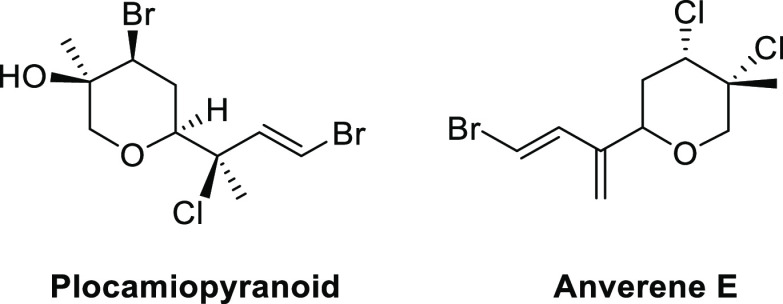
Halogenated marine drugs.

Natural products containing heterocyclic systems have shown to be a remarkable source of bioactive compounds. They show a wide array of activities, such as antitumor, antiviral, antifungal, antifouling, antiproliferative, or anti-inflammatory properties.^1^ Within them, halogenated secondary metabolites play an important role in the development of new therapeutic agents for various pathologies.^2^ In particular, it has been shown that the presence of bromine or chlorine in many of these molecules profoundly influences their bioactivity profile.^3^ Most of these halogenated structures have been isolated from marine organisms such as sponges, fungi, or algae and comprise a large variety of compounds from which tetrahydropyrans have attracted special attention. Examples of compounds of this type include 4-bromo or 4-chloro tetrahydropyrans such as plocamiopyranoid^4^ or anverene *E*,^5^ monoterpenes isolated from Antarctica red algae of the genus *Plocamium cartilagineum* which show promising pharmaceutical properties (Figure 1).

**Scheme 1:**
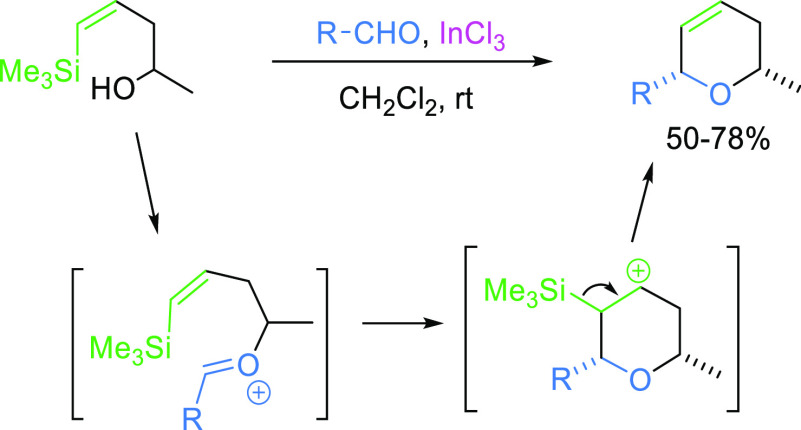
Synthesis of Dihydropyrans by Silyl-Prins Cyclization of *Z*-Vinylsilyl Alcohols

Due to the limited availability of natural sources of these useful products, the efforts of numerous researchers have been devoted to the development of new synthetic methodologies for the preparation of these types of targets. Among the different strategies for the production of tetrahydropyrans,^6^ the Prins cyclization has emerged as a powerful tool for the construction of cyclic ethers in a very efficient and selective manner.^7,8^ The use of electron-rich alkenes, such as alkenylsilanes, in these cyclizations has shown several advantages, including higher reaction rates, higher selectivities, and lower occurrence of secondary reactions.^9,10^ In this type of process, known as silyl-Prins cyclizations, allylsilanes have been frequently used as versatile organosilanes which, depending on their substitution pattern, provide tetrahydropyrans, methylentetrahydropyrans, or dihydropyrans.^11^ In contrast, vinylsilanes have been less commonly employed in such cyclizations. Moreover, reported examples of the utilization of vinylsilanes in silyl-Prins cyclizations are mainly limited to the specific use of *Z*-1-silyl-1-alkenyl derivatives for the synthesis of dihydropyrans.^12^ The mechanism of this process implies the formation of an oxocarbenium ion, by the acid-catalyzed reaction of the silyl-alkenol with the aldehyde, which readily undergoes cyclization to provide the corresponding stabilized β-silyl carbocation. The final loss of the silyl group, with the consequent formation of an endocyclic double bond, affords the final heterocycle (Scheme 1).

**Scheme 2:**
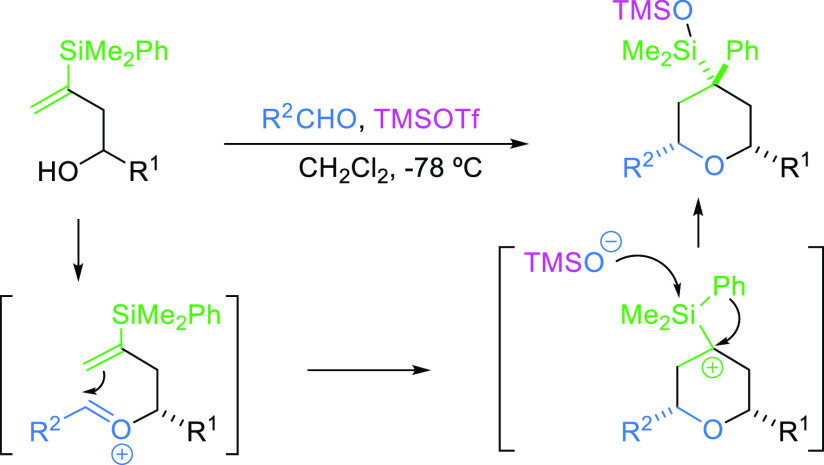
Synthesis of 2,4,4,6-Tetrasubstituted Tetrahydropyrans by Silyl-Prins Cyclization of Vinylsilyl Alcohols

However, only a few examples of silyl-Prins cyclizations using vinylsilyl alcohols in which the silyl group and the chain bearing the alcohol are attached to the same sp^2^ carbon have been reported to date. Within them, we have recently described an interesting process that implies two new features: the cyclization of the vinylsilyl oxocarbenium ion with the formation of an α-silyl carbocation and an unexpected aryl migration from silicon to carbon. The overall reaction affords 2,4,4,6-tetrasubstituted tetrahydropyrans in a very stereoselective manner (Scheme 2).^13^

## Results and Discussion

As shown, this unexpected 1,2-silyl to carbon migration was observed when TMSOTf was used as an activator. We then wondered if the same process would occur when using a metal halide activator such as BiCl_3_. To study that process, we chose the reaction of vinylsilyl alcohol **1a** with cinnamaldehyde, at room temperature, mediated by BiCl_3_ (1 equiv). In contrast to the previous results, the reaction in the presence of BiCl_3_ cleanly provided 4-chloro-tetrahydropyranyl derivative **2h**, in which the silyl group remains in the cycle but not phenyl migration has taken place (Table 1, entry 4). The reaction proceeded with good yield and excellent stereocontrol since a single diastereoisomer is observed.

**Table 1:**
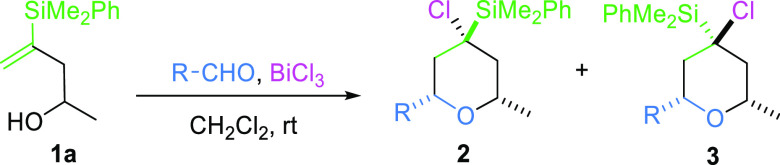
Scope of the BiCl_3_-Mediated Silyl-Prins-Cyclization of **1a**

entry	R	time (min)	ratio **2**/**3**^a^	product^b^ (yield, %)
1	4-MeC_6_H_4_	30	75:25	**2a** + **3a** (44)^c^
2	4-MeOC_6_H_4_	30		CM^d^
3	4-NO_2_C_6_H_4_	40	66:33	**2f** + **3f** (38)^c^
4	(*E*)-PhCH=CH	60	>95:5	**2h** (77)
5	(*E*)-^*n*^PrCH=CH	60		CM^d^

a The ratio of products was determined by ^1^H NMR (400 MHz).

b Conditions: **1a** (1.0 mmol), aldehyde (1.2 mmol), BiCl_3_ (1.0 mmol).

c Other minor unidentified compounds are found in the reaction mixture.

d CM stands for the complex mixture.

We then decided to check the scope and generality of this reaction. For that purpose, we used various aryl and vinyl aldehydes. The results are shown in Table 1.

Although the formation of these 4-chlorotetrahydropyran derivatives was very promising, it was clear that the reaction mediated by BiCl_3_ failed to provide the products with synthetically useful yields. Inspired by Martín and co-workers’ work,^14^ we decided to use TMSCl as a silicon Lewis acid additive which could serve as a chloride source, being now able to employ substoichiometric amounts of BiCl_3_. Fortunately, the reaction under BiCl_3_ (0.05 equiv) catalysis and in the presence of 1 equiv of TMSCl was shown to be a general and efficient process that provided the desired 4-chloro tetrahydropyrans in high yields and with excellent stereoselectivity. This implies that a catalytic amount of BiCl_3_ is able to catalyze the formation of the oxocarbenium ion, while 1 equiv of TMSCl is required to trap the intermediate tetrahydropyranyl carbocation. In Table 2, the scope of the process is shown.

**Table 2:**
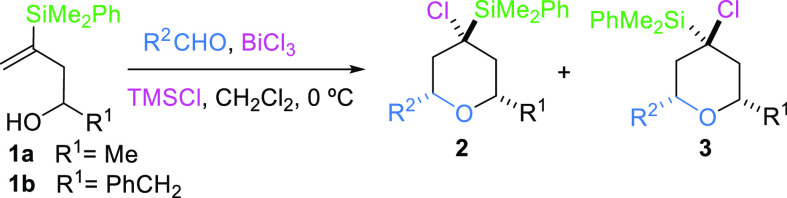

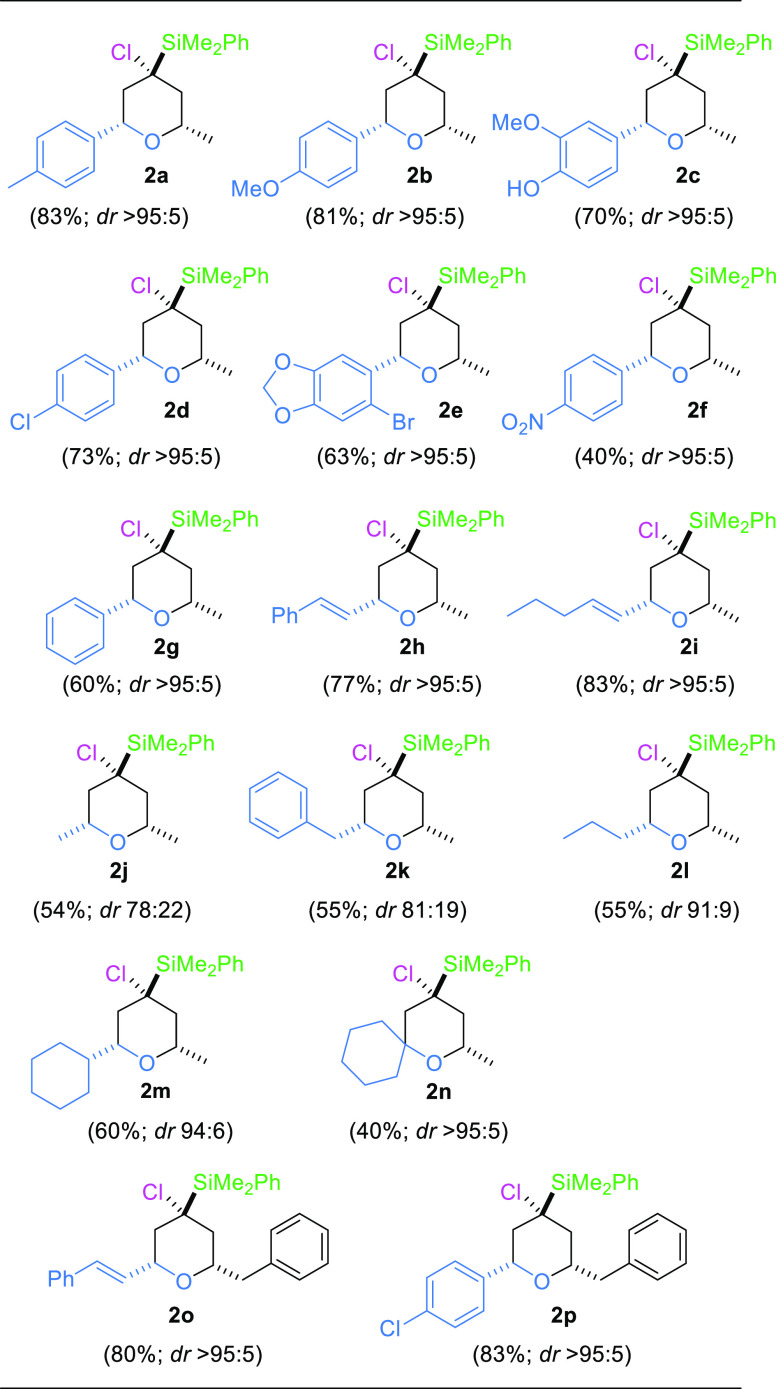
Scope of BiCl_3_/TMSCl-Mediated Silyl-Prins-Cyclization of **1a**^a^

a Diastereoisomer ratios were determined by integration of separated signals in the ^1^H NMR spectra of crude reaction mixtures.

**Scheme 3:**
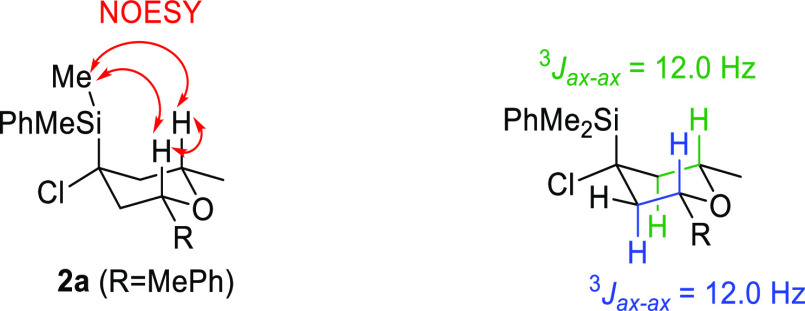
Stereochemistry Assignment

As shown, the reaction with both aromatic (either electron-rich or electron-deficient) and vinylic aldehydes is general and high yielding, providing a single diastereoisomer (**2a**–**2i** and **2o**–**2p**). Good yields are also obtained for aliphatic aldehydes (**2j**–**2m**), although in some cases slightly diminished stereoselectivity is observed. Under the same conditions, the reaction with cyclohexanone provided in moderate yield, but with excellent stereoselectivity, tetrahydropyran **2n**. The relative configuration of stereocenters in tetrahydropyrans **2** was determined on the basis of the NOESY experiment and the measure of coupling constants (Scheme 3).

NOESY correlations of Me-Si and both hydrogens at C2 and C6 positions in **2a** indicate that the three of them are in an axial conformation. Moreover, the axial position of hydrogens at C2 and C6 was readily confirmed by NOESY correlation between them and by the corresponding coupling constants (*J*_H2ax-H3ax_ = 12.0 Hz, and *J*_H5ax-H6ax_ = 12.0 Hz).

**Scheme 4:**
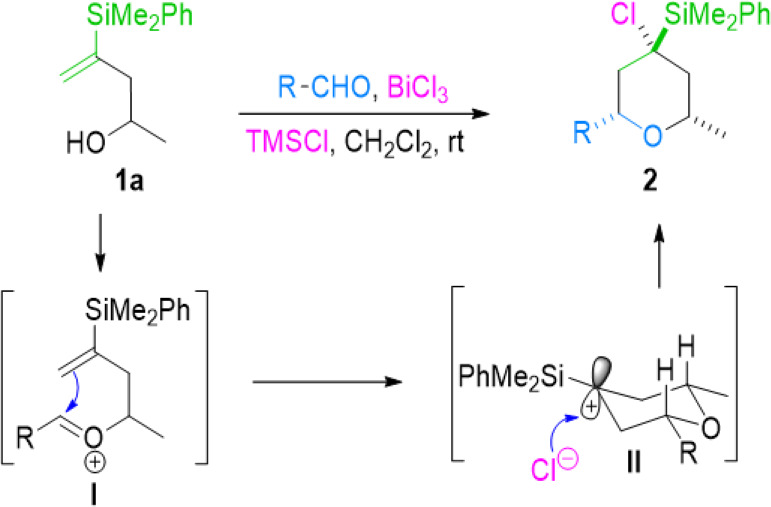
Mechanism of the BiCl_3_-Mediated Silyl-Prins Cyclization of Vinylsilyl Alcohols

The mechanism for this process would imply the preferent formation of an *E*-oxocarbenium anion, which will then undergo 6-*endo* cyclization to provide an α to silicon tertiary carbocation (more stable than the corresponding primary β carbocation).^15^ The final trapping of the tertiary tetrahydropyranyl cation by the chloride would afford the shown product (Scheme 4).

To explain the different outcome of the reaction in the presence of either TMSOTf or BiCl_3_, we hypothesized that the approach of the bulky trimethylsiloxide to the tertiary carbocation may be precluded on the steric ground, while a small nucleophile, such as chloride, would have a relatively clear path to attack the tetrahydropyranyl carbocation. However, other stereoelectronic factors (such as the strength of the bond formed) probably also have an important role and further theoretical calculation would be needed to obtain a rationale for the different behavior of both Lewis acids.

Moreover, the high stereoselectivity observed in the process can be rationalized, according to the theoretical studies by Alder on Prins cyclization,^16^ by a preferred chair-like transition state in which the substituents in C2 and C6 adopt the most stable equatorial position. The subsequent nucleophilic attack (by the chloride provided by the trimethylsilylchloride) over the intermediate tetrahydropyranyl cation thus obtained will then occur through the less hindered equatorial side (Scheme 4).^17^

Although aware of the frequent occurrence of the competitive oxonia-Cope rearrangement in Prins cyclization when the alkenol has an adjacent group to the alcohol able to stabilize the positive charge,^18,19^ we tested the reaction of alcohols **1c** (R^1^ = (*E*)-PhCH=CH) and **1d** (R^1^ = 4-ClPh) with phenylacetaldehyde (R^2^ = Ph-CH_2_). The reaction gave either a lower yield of the corresponding tetrahydropyran (**2o**, 37%) or a complex mixture from which it was difficult to isolate **2p**.^20^ Fortunately, the same tetrahydropyranyl derivatives (**2o** and **2p**) could be obtained in high yield and selectivity by exchanging the substituents (R^1^ = Ph-CH_2_; R^2^ = (*E*)-PhCH=CH or 4-ClPh) in the alcohol and aldehyde (as shown in Table 1). Thus, the synthetic flexibility of this methodology, as two complementary vinylsilyl alcohol/aldehyde combinations can be explored to produce a specific substituted tetrahydroyranyl derivative, increases the chances of a successful outcome.

**Scheme 5:**
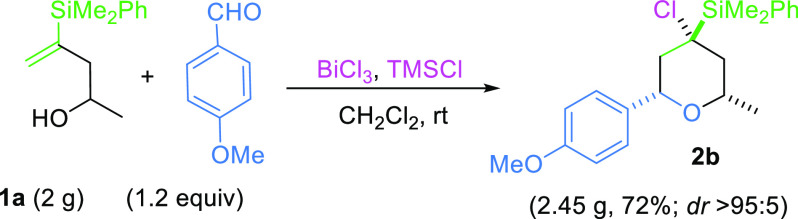
Gram-Scale silyl-Prins Cyclization

In order to show the potential applicability of this procedure, we decided to test a gram-scale experiment, under the standard conditions, as shown in Scheme 5. To our delight, the corresponding polysusbstituted tetrahydropyranyl derivative **2b** was obtained in good yield and excellent stereoselectivity.

**Scheme 6:**
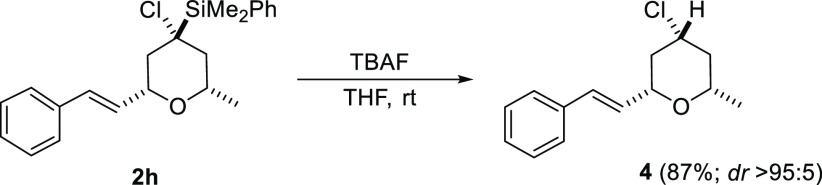
Stereoselective Desilylation Process

To generate further value from this methodology, we needed to be able to control the interconversion of functional groups at the quaternary C4 in a stereoselective manner. For this purpose, we chose a desilylation process. Fortunately, treatment of compound **2n** with TBAF provided the corresponding 4-clorotetrahydropyran **4** in high yield and with total retention of the configuration (Scheme 6).^21^ It has to be noticed that a desilylation process at quaternary carbon may be a challenging process, which has been reported to occur with either retention,^22^ inversion,^23^ or loss of stereocontrol.^24^

**Scheme 7:**
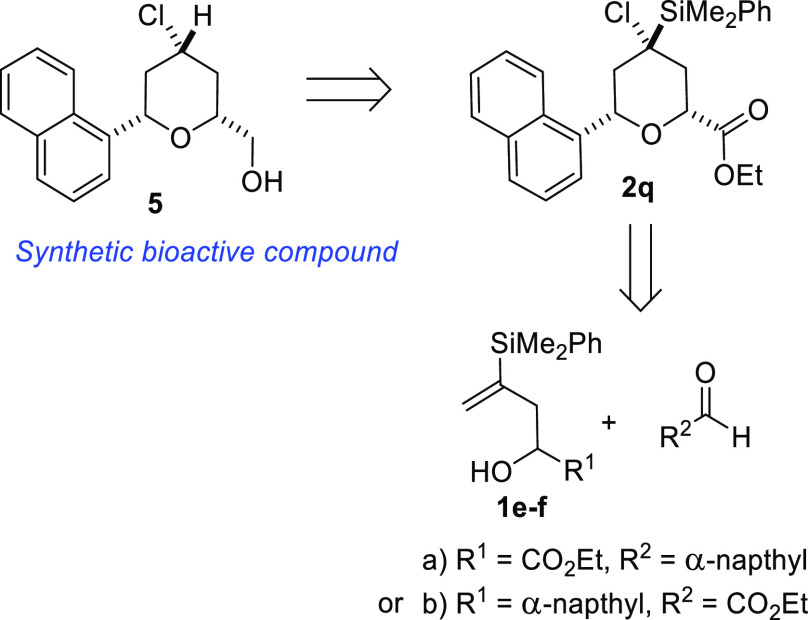
Retrosynthetic Route for Bioactive Compound **5**

With these promising results in hand, we next decided to apply this methodology to the synthesis of biologically and pharmaceutically active compounds. For that purpose, we chose a known synthetic bioactive molecule **5**, related to the structure of naproxen, which exhibits antinociceptive activity.^25^ The key intermediate to be obtained would be tetrahydropyran **2q**, which would be accessed using the described methodology (Scheme 7).

**Scheme 8:**
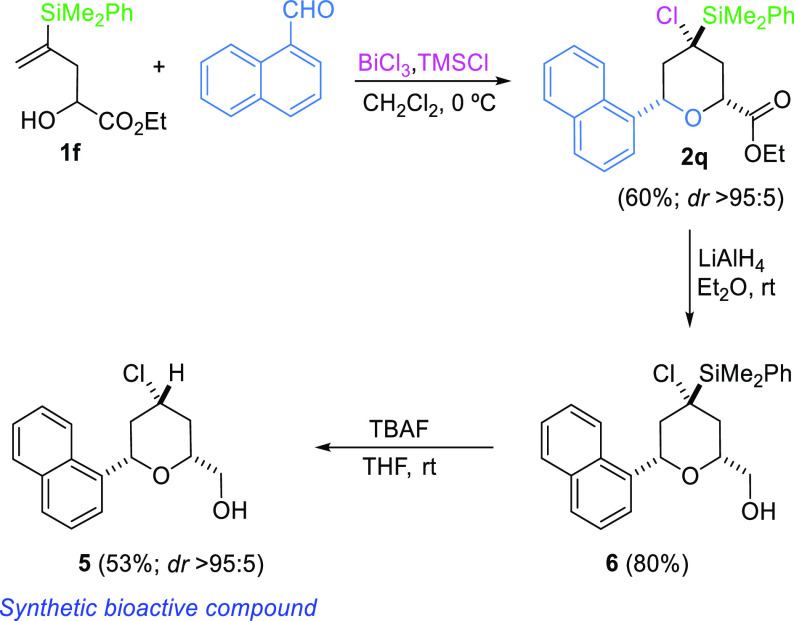
Synthesis of 2,4,4,6-Tetrasubstituted Tetrahydropyrans by Silyl-Prins Cyclization of Vinylsilyl Alcohols

From the two possibilities of accessing **2q**, the reaction of alcohol **1e** (R^1^ = α-naphthyl) with ethyl glyoxylate leads to a complex mixture, from which we could not isolate any cyclic product. However, when the alcohol bears a group R^1^ = CO_2_Et (**1f**) and the α-naphthyl moiety is introduced in the aldehyde, the desired heterocycle **2q** could be isolated in a satisfying 60% (Scheme 8). Interestingly, a single 2,6-*cis*-tetrahydropyran **2q** was obtained, despite Loh’s report on the formation of 2,6-*trans*-tetrahydropyranyl derivatives in Prins cyclization when the starting alcohol bears an α-alkoxycarbonyl group.^26^

Once the desired precursor (**2q**) of bioactive compound **5** was obtained, two further steps (desilylation and reduction of the ester) were needed to synthesize the final target. Since trying to remove the silyl moiety, by the reaction of **2q** with TBAF, led to the degradation of the starting material, we decided to apply the reduction step first (Scheme 8). Thus, treatment of **2q** with LiAlH_4_ produced the hydroxyl derivative **6** in 80% yield. After purification, **6** was subjected to desilylation with excess TBAF to provide the desired bioactive compound **5** in 53% yield and with excellent stereoselectivity.

In conclusion, we have developed a general procedure for the synthesis of polysubstituted halogenated tetrahydropyrans in a one-pot reaction in which tertiary and quaternary stereogenic centers are created with high stereoselectivity. Interestingly, a change in the catalyst employed (from TMSOTf to BiCl_3_) has proceeded with a modification of the reaction pathway (from cyclization with tandem silicon to carbon aryl-migration, to cyclization to give an α-silyl carbocation with subsequent stereoselective capture by the Lewis acid counteranion). An interesting methodology has been applied to the synthesis of a known bioactive compound with antinociceptive activity.

## Experimental Section

### General Information

Unless otherwise noted, experiments were carried out with dry solvents under nitrogen atmosphere. Dichloromethane was dried with preactivated molecular sieves. Flash column chromatography was performed using Silica Gel 60 (230–400 mesh ASTM). Thin layer chromatography (TLC) was performed using an aluminum backed plate, precoated with silica gel (0.20 mm, silica gel 60) with a fluorescent indicator (254 nm) from Macherey. NMR spectra were recorded at nuclear magnetic resonance service of the Laboratory of Instrumental Techniques (L.T.I., www.laboratoriotecnicasinstrumentales.es), University of Valladolid at Varian 400 MHz (1 H, 399.85 MHz; 13C, 100.61 MHz), Varian 500 MHz (^1^H, 500.12 MHz; ^13^C, 100.61 MHz) spectrometers at room temperature (25 °C). Chemical shifts (δ) were reported in parts per million (ppm) relative to the residual solvent peaks recorded, rounded to the nearest 0.01 for 1 H-NMR and 0.1 for ^13^C-NMR (reference: CDCl_3_ [^1^H: 7.26, ^13^C: 77.2]). Spin–spin coupling constants (*J*) in ^1^H-NMR were given in Hz to the nearest 0.1 Hz, and peak multiplicity was indicated as follows s (singlet), d (doublet), t (triplet), q (quartet), m (multiplet), and br (broad). ^13^C {H} NMR was recorded with complete proton decoupling. Carbon types, structure assignments, and attribution of peaks were determined from two-dimensional correlation experiments (HSQC, COSY, and HMBC). Relative stereochemistry was assigned based on the 2D-NOE experiments. High-resolution mass spectra (HRMS) were measured at the mass spectrometry service of the Laboratory of Instrumental Techniques, University of Valladolid, using a quadrupole spectrometer equipped with a TOF analyzer, on a UPLC-MS system (UPLC: Waters ACQUITY H-class UPLC; MS: Bruker Maxis Impact) by electrospray ionization (ESI positive and negative).

### General Procedure for the BiCl_3_/TMSCl-Promoted Cyclization

TMSCl (0.076 mL, 0.6 mmol, 1.2 equiv) was slowly introduced into a suspension of BiCl_3_ (7.9 mg, 0.025 mmol, 0.05 equiv) in 4.8 mL of dichloromethane, containing the corresponding aldehyde (0.6 mmol, 1.2 equiv) and cooled to 0 °C. The mixture is stirred for 5 min and a solution of alcohol **1a** or **1b** (0.5 mmol, 1 equiv) in 0.2 mL of dichloromethane is added dropwise and the reaction is followed by TLC. When starting materials are consumed (typically 30 min–1 h), it is partly evaporated and filtered through a small plug of silica. Volatiles are evaporated under reduced pressure. The crude mixture is purified by column chromatography (mixtures of hexane/ethyl acetate) yielding compounds **2a–p**.

#### (2S*,4R*,6S*)-4-Chloro-4-(dimethyl(phenyl)silyl)-2-methyl-6-(p-tolyl)tetrahydro-2*H*-pyran (**2a**)

Faint yellow oil. According to the general procedure, the title compound **2a** was obtained from alcohol **1a** (110 mg, 0.500 mmol) and 4-tolualdehyde to give, after column chromatography (hexane/EtOAc: 40:1), a yellow oil (149 mg, 83%). ^1^H NMR (500 MHz, CDCl_3_) δ 7.68–7.62 (m, 2H), 7.45–7.40 (m, 3H), 7.11 (s, 4H), 4.14 (dd, *J* = 12.0, 2.0 Hz, 1H), 3.55–3.44 (m, 1H), 2.47 (dt, *J* = 13.5, 1.9 Hz, 1H), 2.40 (dt, *J* = 13.4, 1.9 Hz, 1H), 2.31 (s, 3H), 2.07 (dd, *J* = 13.5, 12.0 Hz, 1H), 1.90 (dd, *J* = 13.4, 11.6 Hz, 1H), 1.17 (d, *J* = 6.1 Hz, 3H), 0.59 (s, 6H). ^13^C {H} NMR (101 MHz, CDCl_3_) δ 138.9, 137.3, 136.9, 134.7, 129.9, 129.0, 128.1, 125.8, 76.9, 71.4, 60.9, 46.9, 46.8, 22.1, 21.1, −3.2 (CH_3_Si), −3.6. HRMS (ESI^+^) *m/z* calcd for C_21_ClH_27_NaOSi ([M + Na]^+^): 381.1412, found 381.1420.

#### (2S*,4R*,6S*)-4-Chloro-4-(dimethyl(phenyl)silyl)-2-(4-methoxyphenyl)-6-methyltetrahydro-2*H*-pyran (**2b**)

Yellowish solid (mp: 72.3–73.7 °C). According to the general procedure, the title compound **2b** was obtained from alcohol **1a** (110 mg, 0.500 mmol) and anisaldehyde to give, after column chromatography (hexane/EtOAc: 30:1), a yellowish solid (152 mg, 81%). ^1^H NMR (400 MHz, CDCl_3_) δ 7.66–7.59 (m, 2H), 7.45–7.36 (m, 3H), 7.17–7.09 (m, 2H), 6.88–6.78 (m, 2H), 4.10 (dd, *J* = 11.9, 2.0 Hz, 1H), 3.76 (s, 3H), 3.53–3.41 (m, 1H), 2.44 (d, *J* = 13.5 Hz, 1H), 2.38 (d, *J* = 13.4 Hz, 1H), 2.06 (dd, *J* = 13.5, 11.9 Hz, 1H), 1.88 (dd, *J* = 13.4, 11.6 Hz, 1H), 1.14 (d, *J* = 6.1 Hz, 3H), 0.57 (s, 6H). ^13^C {H} NMR (101 MHz, CDCl_3_) δ 159.1 (C, p-OMe), 137.0, 134.7, 134.1, 129.9, 128.1, 127.1, 113.8, 76.6, 71.4, 60.9, 55.3, 46.9, 46.8, 22.1, −3.2, −3.6. HRMS (ESI^+^) *m/z* calcd for C_21_ClH_27_NaO_2_Si ([M + Na]^+^): 397.1361, found 397.1371.

#### (2S*,4R*,6S*)-4-Chloro-4-(dimethyl(phenyl)silyl)-6-(4-hydroxy-3-methoxyphenyl)-2-methyltetrahydro-2*H*-pyran (**2c**)

Off-white solid (mp: 78.8–80.8 °C). According to the general procedure, the title compound **2c** was obtained from alcohol **1a** (110 mg, 0.500 mmol) and vanillin to give, after column chromatography (hexane/EtOAc: 10:1), an off-white solid (137 mg, 70%). ^1^H NMR (400 MHz, CDCl_3_) δ 7.67–7.60 (m, 2H), 7.46–7.39 (m, 3H), 6.86–6.80 (m, 1H), 6.78–6.73 (m, 1H), 6.68–6.62 (m, 1H), 5.54 (s, 1H, OH), 4.08 (d, *J* = 11.6 Hz, 1H), 3.89 (s, 3H), 3.54–3.44 (m, 1H), 2.45 (d, *J* = 13.8 Hz, 1H) 2.40 (d, *J* = 13.4 Hz, 1H), 2.07 (dd, *J* = 13.8, 11.6 Hz, 1H), 1.90 (dd, *J* = 13.4, 12.0 Hz, 1H), 1.17 (d, *J* = 6.1 Hz, 3H), 0.59 (s, 6H). ^13^C {H} NMR (101 MHz, CDCl_3_) δ 146.5, 145.2, 136.9, 134.7, 133.9, 130.0, 128.1, 119.1, 114.0, 108.4, 77.0, 71.5, 60.9, 55.9, 46.9, 46.8, 22.1, −3.1, −3.6. HRMS (ESI^+^) *m/z* calcd for C_21_ClH_27_NaO_3_Si ([M + Na]^+^): 413.1310, found 413.1313.

#### (2S*,4R*,6S*)-4-Chloro-4-(dimethyl(phenyl)silyl)-2-(4-chlorophenyl)-6-methyltetrahydro-2*H*-pyran (**2d**)

Faint yellow liquid. According to the general procedure, the title compound **2d** was obtained from alcohol **1a** (110 mg, 0.500 mmol) and 4-chlorobenzaldehyde to give, after column chromatography (hexane/EtOAc: 25:1), a yellow oil (138 mg, 73%). ^1^H NMR (500 MHz, CDCl_3_) δ 7.66–7.61 (m, 2H, Ph), 7.45–7.39 (m, 3H, Ph), 7.27 (d, *J* = 8.8 Hz, 2H), 7.14 (d, *J* = 8.8 Hz, 2H), 4.12 (dd, *J* = 12.0, 2.0 Hz, 1H), 3.53–3.45 (m, 1H), 2.48–2.37 (m, 2H), 1.99 (dd, *J* = 13.5, 12.0 Hz, 1H), 1.89 (dd, *J* = 13.3, 11.7 Hz, 1H), 1.17 (d, *J* = 6.1 Hz, 3H, CH_3_), 0.59 (s, 3H, CH_3_-Si), 0.59 (s, 3H, CH_3_-Si). ^13^C {H} NMR (101 MHz, CDCl_3_) δ 140.6, 137.0, 134.8, 133.4, 130.2, 128.7, 127.3, 76.4, 71.7, 60.6, 47.1, 46.9, 22.2, −3.1, −3.5. HRMS (ESI^+^) *m/z* calc. For C_20_H_24_Cl_2_NaOSi ([M + Na]^+^): 401.0866, found 401.0872.

#### (2S*,4R*,6S*)-2-(6-Bromobenzo[d][1,3]dioxol-5-yl)-4-chloro-4-(dimethyl(phenyl)silyl)-6-methyltetrahydro-2*H*-pyran (**2e**)

Yellowish solid (mp: 105.6–107.7 °C). According to the general procedure, the title compound **2e** was obtained from alcohol **1a** (110 mg, 0.500 mmol) and 6-bromopiperonal to give, after column chromatography (hexane/EtOAc: 30:1), a colorless oil (147 mg, 63%). ^1^H NMR (500 MHz, CDCl_3_) δ 7.72–7.68 (m, 2H), 7.43–7.35 (m, 3H), 7.04 (s, 1H), 6.93 (s, 1H), 5.95–5.93 (m, 2H), 4.63 (dd, *J* = 11.9, 2.0 Hz, 1H), 3.60–3.51 (m, 1H), 2.62 (dt, *J* = 13.5, 2.0 Hz, 1H), 2.36 (dt, *J* = 13.4, 1.9 Hz, 1H), 1.84 (dd, *J* = 13.4, 11.6 Hz, 1H), 1.79 (dd, *J* = 13.5, 11.9 Hz, 1H), 1.18 (d, *J* = 6.1 Hz, 3H), 0.66 (s, 3H), 0.62 (s, 3H). ^13^C {H} NMR (101 MHz, CDCl_3_) δ 147.8, 147.6, 136.1, 135.0, 134.9, 129.8, 127.8, 112.1, 111.5, 107.6, 101.7, 76.0, 71.3, 60.5, 46.5, 45.6, 22.0, −2.6, −2.8. HRMS (ESI^+^) *m/z* calcd for C_21_BrClH_24_NaO_3_Si ([M + Na]^+^): 489.0259, found 489.0266.

#### (2S*,4R*,6S*)-4-Chloro-4-(dimethyl(phenyl)silyl)-2-methyl-6-(4-nitrophenyl)tetrahydro-2*H*-pyran (**2f**)

Faint yellow oil. According to the general procedure, the title compound **2f** was obtained from alcohol **1a** (110 mg, 0.500 mmol) and 4-nitrobenzaldehyde to give, after column chromatography (hexane/EtOAc: 30:1), a yellow oil (78 mg, 40%). ^1^H NMR (400 MHz, CDCl_3_) δ 8.17–8.10 (m, 2H), 7.68–7.59 (m, 2H), 7.46–7.40 (m, 3H), 7.38–7.32 (m, 2H), 4.20 (dd, *J* = 12.2, 2.0 Hz, 1H), 3.56–3.45 (m, 1H), 2.51–2.38 (m, 2H), 1.98–1.84 (m, 2H), 1.18 (d, *J* = 6.1 Hz, 3H), 0.60 (s, 3H), 0.59 (s, 3H). ^13^C {H} NMR (101 MHz, CDCl_3_) δ 149.2, 147.2, 136.7, 134.6, 130.2, 128.3, 126.3, 123.6, 75.9, 71.6, 59.9, 46.8, 46.6, 21.9, −3.3, −3.7. HRMS (ESI^+^) *m/z* calcd for C_20_ClH_24_NNaO_3_Si ([M + Na]^+^): 412.1106, found 412.1112.

#### (2S*,4R*,6S*)-4-Chloro-4-(dimethyl(phenyl)silyl)-2-methyl-6-phenyltetrahydro-2*H*-pyran (**2g**)

Faint yellow oil. According to the general procedure, the title compound **2g** was obtained from alcohol **1a** (110 mg, 0.500 mmol) and benzaldehyde to give, after column chromatography (hexane/EtOAc: 30:1), a yellow oil (103 mg, 60%). ^1^H NMR (500 MHz, CDCl_3_) δ 7.67–7.62 (m, 2H), 7.45–7.41 (m, 3H), 7.32–7.28 (m, 2H), 7.25–7.20 (m, 3H), 4.17 (dd, *J* = 12.0, 2.0 Hz, 1H), 3.54–3.46 (m, 1H), 2.49 (dt, *J* = 13.6, 2.0 Hz, 1H), 2.41 (dt, *J* = 13.4, 1.9 Hz, 1H), 2.06 (dd, *J* = 13.6, 12.0 Hz, 1H), 1.91 (dd, *J* = 13.4, 11.7 Hz, 1H), 1.17 (d, *J* = 6.1 Hz, 3H), 0.60 (s, 3H), 0.59 (s, 3H). ^13^C {H} NMR (101 MHz, CDCl_3_) δ 142.0, 137.0, 134.8, 130.0, 128.4, 128.2, 127.7, 125.8, 77.0, 71.5, 60.9, 47.0, 46.9, 22.1, −3.2, −3.5. HRMS (ESI^+^) *m/z* calcd for C_20_ClH_25_NaOSi ([M + Na]^+^): 367.1255, found 367.1260.

#### (2S*,4R*,6S*)-4-Chloro-4-(dimethyl(phenyl)silyl)-2-methyl-6-((*E*)-styryl)tetrahydro-2*H*-pyran (**2h**)

Yellowish viscous oil. According to the general procedure, the title compound **2h** was obtained from alcohol **1a** (110 mg, 0.500 mmol) and *trans*-cinnamaldehyde to give, after column chromatography (hexane/EtOAc: 30:1), a yellowish solid (143 mg, 77%). ^1^H NMR (400 MHz, CDCl_3_) δ 7.64–7.58 (m, 2H), 7.44–7.38 (m, 3H), 7.37–7.32 (m, 2H), 7.32–7.26 (m, 2H), 7.26–7.19 (m, 1H), 6.45 (d, *J* = 16.0 Hz, 1H), 6.08 (dd, *J* = 16.0, 6.4 Hz, 1H), 3.84 (dd, *J* = 11.8, 6.4 Hz, 1H), 3.55–3.44 (m, 1H), 2.43 (d, *J* = 13.4 Hz, 1H), 2.35 (d, *J* = 13.5 Hz, 1H), 1.98 (dd, *J* = 13.4, 11.8 Hz, 1H), 1.84 (dd, *J* = 13.5, 11.6 Hz, 1H), 1.16 (d, *J* = 6.1 Hz, 3H), 0.59 (s, 3H), 0.58 (s, 3H). ^13^C {H} NMR (101 MHz, CDCl_3_) δ 136.8, 136.5, 134.7, 131.0, 130.0, 129.3, 128.5, 128.1, 127.7, 126.5, 75.6, 71.0, 60.4, 46.7, 44.8, 22.0, −3.2, −3.5. HRMS (ESI^+^) *m/z* calcd for C_22_ClH_27_NaOSi ([M + Na]^+^): 393.1412, found 393.1406.

#### (2S*,4R*,6S*)-4-Chloro-4-(dimethyl(phenyl)silyl)-2-methyl-6-((*E*)-pent-1-en-1-yl)tetrahydro-2*H*-pyran (**2i**)

Faint yellow oil. According to the general procedure, the title compound **2i** was obtained from alcohol **1a** (110 mg, 0.500 mmol) and *trans*-2-hexen-1-al to give, after column chromatography (hexane/EtOAc: 30:1), a yellow oil (140 mg, 83%). ^1^H NMR (400 MHz, CDCl_3_) δ 7.58–7.54 (m, 2H), 7.40–7.34 (m, 3H), 5.53 (dtd, *J* = 15.5, 6.6, 1.0 Hz, 1H), 5.33 (ddt, *J* = 15.5, 6.7, 1.5 Hz, 1H), 3.62 (dd, *J* = 11.8, 6.7 Hz, 1H), 3.41–3.30 (m, 1H), 2.32–2.26 (m, 2H), 1.99–1.92 (m, 2H), 1.86 (dd, *J* = 13.7, 11.8 Hz, 1H), 1.76 (dd, *J* = 13.6, 11.6 Hz, 1H), 1.41–1.32 (m, 2H), 1.10 (d, *J* = 6.1 Hz, 3H), 0.87 (t, *J* = 7.4 Hz, 3H), 0.54 (s, 3H) 0.53 (s, 3H). ^13^C {H} NMR (101 MHz, CDCl_3_) δ 136.8, 134.7, 132.9, 130.1, 129.8, 128.0, 75.8, 70.8, 60.7, 46.7, 45.1, 34.3, 22.1, 22.0, 13.7, −3.1, −3.5. HRMS (ESI^+^) *m/z* calcd for C_19_ClH_29_NaOSi ([M + Na]^+^): 359.1568, found 359.1573.

#### (2R*,4r*,6S*)-4-Chloro-4-(dimethyl(phenyl)silyl)-2,6-dimethyltetrahydro-2*H*-pyran (**2j**)

Faint yellow oil. According to the general procedure, the title compound **2j** was obtained from alcohol **1a** (110 mg, 0.500 mmol) and acetaldehyde to give, after column chromatography (hexane/EtOAc: 30:1), a yellow oil (76 mg, 54%) as a mixture of stereoisomers. ^1^H NMR (400 MHz, CDCl_3_) δ 7.61–7.52 (m, 2H), 7.44–7.33 (m, 3H), 3.39–3.31 (m, 2H), 2.29 (d, *J* = 13.8 Hz, 2H), 1.75 (dd, *J* = 13.8, 11.7 Hz, 1H), 1.10 (d, *J* = 6.2, 6H), 0.53 (s, 6H). ^13^C {H} NMR (101 MHz, CDCl_3_) δ 136.6, 134.7, 129.8, 127.9, 70.9, 60.7, 46.6, 22.0, −3.1. HRMS (ESI^+^) *m/z* calcd for C_15_ClH_23_NaOSi ([M + Na]^+^): 305.1099, found 305.1100.

#### (2R*,4s*,6S*)-4-Chloro-4-(dimethyl(phenyl)silyl)-2,6-dimethyltetrahydro-2*H*-pyran (**3j**) (Minor) (Distinguishable Signals)

^1^H NMR (400 MHz, CDCl_3_) δ 7.61–7.52 (m, 2H), 7.44–7.33 (m, 3H), 4.05–3.97 (m, 2H), 1.78–1.72 (m, 2H), 1.53–1.46 (m, 2H), 1.13–1.11 (m, 6H), 0.44 (s, 6H). ^13^C {H} NMR (101 MHz, CDCl_3_) δ 134.8, 129.7, 127.7, 67.5, 62.8, 40.5, 21.5, −6.5.

#### (2R*,4R*,6S*)-2-Benzyl-4-chloro-4-(dimethyl(phenyl)silyl)-6-methyltetrahydro-2*H*-pyran (**2k**)

Colorless oil. According to the general procedure, the title compound **2k** was obtained from alcohol **1a** (110 mg, 0.500 mmol) and phenylacetaldehyde to give, after column chromatography (hexane/EtOAc: 30:1), a colorless oil (99 mg, 55%) as a mixture of stereoisomers. ^1^H NMR (400 MHz, CDCl_3_) δ 7.41–7.22 (m, 7H), 7.15–7.07 (m, 3H), 3.44–3.32 (m, 2H), 2.90 (dd, *J* = 13.2, 5.2 Hz, 1H), 2.50 (dd, *J* = 13.2, 8.4 Hz, 1H), 2.32 (d, *J* = 13.4 Hz, 1H), 2.24 (d, *J* = 13.3 Hz, 1H), 1.80 (dd, *J* = 13.4, 11.7 Hz, 1H), 1.71 (dd, *J* = 13.3, 11.6 Hz, 1H), 1.12 (d, *J* = 6.1 Hz, 3H), 0.45 (s, 3H), 0.38 (s, 3H). ^13^C {H} NMR (101 MHz, CDCl_3_) δ 137.7, 136.5, 134.7, 129.6, 129.4, 128.3, 127.9, 126.4, 76.1, 71.2, 60.9, 46.9, 43.8, 42.8, 22.0, −3.0, −3.8. HRMS (ESI^+^) *m/z* calcd for C_21_ClH_27_NaOSi ([M + Na]^+^): 381.1412, found 381.1418.

#### (2R*,4r*,6S*)-4-Chloro-4-(dimethyl(phenyl)silyl)-2,6-dimethyltetrahydro-2*H*-pyran (**3k**) (Minor) (Distinguishable Signals)

^1^H NMR (400 MHz, CDCl_3_) δ 4.13–3.94 (m, 2H), 2.83 (dd, *J* = 13.6, 6.4 Hz, 1H), 2.57 (dd, *J* = 13.6, 5.0 Hz, 1H), 1.58–1.48 (m, 2H), 1.12–1.09 (m, 3H), 0.42 (s, 3H), 0.41 (s, 3H). ^13^C {H} NMR (101 MHz, CDCl_3_) δ 138.6, 134.8, 129.7, 129.3, 128.1, 127.8, 126.1, 72.4, 67.6, 62.5, 42.2, 40.6, 38.6, 21.3, −6.5.

#### (2S*,4R*,6R*)-4-Chloro-4-(dimethyl(phenyl)silyl)-2-methyl-6-propyltetrahydro-2*H*-pyran (**2l**)

Faint yellow liquid. According to the general procedure, the title compound **2l** was obtained from alcohol **1a** (110 mg, 0.500 mmol) and butyraldehyde to give, after column chromatography (hexane/EtOAc: 30:1), a yellow oil (86 mg, 55%). ^1^H NMR (500 MHz, CDCl_3_) δ 7.58–7.55 (m, 2H, Ph), 7.43–7.36 (m, 3H, Ph), 3.35–3.28 (m, 1H), 3.19–3.14 (m, 1H), 2.31 (d, *J* = 13.4 Hz, 2H), 1.77 (dd, *J* = 13.4, 11.9 Hz, 1H), 1.73 (dd, *J* = 13.4, 11.7 Hz, 1H), 1.46–1.40 (m, 1H), 1.35–1.23 (m, 3H), 1.10 (d, *J* = 6.1 Hz, 3H, CH_3_), 0.83 (t, *J* = 7.1 Hz, 3H, CH_3_), 0.53 (s, 6H, Si-CH_3_). ^13^C {H} NMR (101 MHz, CDCl_3_) δ 136.9, 134.9, 129.9, 128.1, 74.8, 71.1, 61.3, 47.2, 45.1, 38.6, 22.2, 18.8, 14.1, −2.9, −3.0. HRMS (ESI^+^) *m/z* calcd for C_17_H_27_ClNaOSi ([M + Na]^+^): 333.1412, found 333.1420.

#### (2S*,4R*,6S*)-4-Chloro-2-cyclohexyl-4-(dimethyl(phenyl)silyl)-6-methyltetrahydro-2*H*-pyran (**2m**)

Faint yellow oil. According to the general procedure, the title compound **2m** was obtained from alcohol **1a** (110 mg, 0.500 mmol) and cyclohexane carboxaldehyde to give, after column chromatography (hexane/EtOAc: 30:1), a yellow oil (105 mg, 60%). ^1^H NMR (500 MHz, CDCl_3_) δ 7.58–7.53 (m, 2H, Ph), 7.42–7.34 (m, 3H, Ph), 3.33–3.25 (m, 1H), 2.92 (ddd, *J* = 11.9, 6.6, 1.6 Hz, 1H), 2.36–2.26 (m, 2H), 1.83–1.77 (m, 1H, Cy) 1.76–1.71 (m, 2H), 1.70–1.59 (m, 4H, Cy), 1.32–1.23 (m, 1H, Cy), 1.23–1.11 (m, 3H, Cy), 1.09 (d, *J* = 6.2 Hz, 3H, CH_3_), 0.96–0.87 (m, 1H, Cy), 0.87–0.78 (m, 1H, Cy), 0.53 (s, 6H, Si-CH_3_). ^13^C {H} NMR (101 MHz, CDCl_3_) δ 136.9, 134.9, 129.9, 128.0, 79.3, 71.2, 62.0, 47.4, 43.1, 42.0, 29.3, 28.5, 26.7, 26.2, 26.1, 22.1, −2.8, −3.0. HRMS (ESI^+^) *m/z* calcd for C_20_H_31_ClNaOSi ([M + Na]^+^): 373.1725, found 373.1729.

#### (2S*,4R*)-4-Chloro-4-(dimethyl(phenyl)silyl)-2-methy-1-oxaspiro[5.5]undecane (**2n**)

Faint yellow oil. According to the general procedure, the title compound **2n** was obtained from alcohol **1a** (110 mg, 0.500 mmol) and cyclohexanone to give, after column chromatography (hexane/EtOAc: 30:1), a yellow oil (67 mg, 40%). ^1^H NMR (500 MHz, CDCl_3_) δ 7.60–7.55 (m, 2H, Ph), 7.43–7.34 (m, 3H, Ph), 3.47–3.37 (m, 1H), 2.19 (dd, *J* = 14.7, 4.7 Hz, 1H), 1.98 (d, *J* = 15.1 Hz, 1H), 1.93–1.89 (m, 1H), 1.91 (dd. *J* = 14.7, 9.4 Hz, 1H), 1.78 (d, *J* = 15.1 Hz, 1H), 1.71–1.63 (m, 1H), 1.62–1.57 (m, 1H), 1.53–1.47 (m, 2H, Cy), 1.45–1.36 (m, 2H, Cy), 1.31–1.26 (m, 2H, Cy), 1.22–1.19 (m, 1H, Cy), 1.15 (d, *J* = 6.2 Hz, 3H, CH_3_), 0.46 (s, 6H, Si-CH_3_). ^13^C {H} NMR (101 MHz, CDCl_3_) δ 135.6, 134.9, 129.8, 127.9, 73.1, 63.4, 59.0, 42.1, 42.0, 40.3, 36.8, 25.9, 23.1, 22.4, 22.3, −5.1, −5.5. HRMS (ESI^+^) *m/z* calcd for C_19_H_29_ClNaOSi ([M + Na]^+^): 359.1568, found 359.1578.

#### (2R*,4S*,6R*)-2-Benzyl-4-chloro-4-(dimethyl(phenyl)silyl)-6-((*E*)-styryl)tetrahydro-2*H*-pyran (**2o**)

Faint yellow oil. According to the general procedure, the title compound **2o** was obtained from alcohol **1b** (148 mg, 0.500 mmol) and *trans*-cinnamaldehyde to give, after column chromatography (hexane/EtOAc: 30:1), a yellow oil (179 mg, 80%). ^1^H NMR (400 MHz, CDCl_3_) δ 7.46–7.12 (m, 15H, Ph), 6.48 (d, *J* = 16.0 Hz, 1H, Ph-CH=), 6.10 (dd, *J* = 16.0, 6.1 Hz, 1H), 3.89 (dd, *J* = 12.0, 6.1 Hz, 1H), 3.53–3.45 (m, 1H), 2.98 (dd, *J* = 13.2, 5.1 Hz, 1H), 2.57 (dd, *J* = 13.2, 8.4 Hz, 1H), 2.45 (d, *J* = 13.5 Hz, 1H), 2.31 (d, *J* = 13.6 Hz, 1H), 2.02 (dd, *J* = 13.5, 12.0 Hz, 1H), 1.80 (dd, *J* = 13.6, 11.7 Hz, 1H), 0.52 (s, 3H, SiCH_3_), 0.40 (s, 3H, SiCH_3_). ^13^C {H} NMR (101 MHz, CDCl_3_) δ 137.6, 136.7, 136.5, 134.7, 130.9, 129.9, 129.5, 129.2, 128.5, 128.4, 128.1, 127.7, 126.5, 126.4, 76.1, 75.7, 60.6, 45.1, 43.9, 42.8, −3.2, −4.1. HRMS (ESI^+^) *m/z* calcd for C_28_H_31_ClNaOSi ([M + Na]^+^): 469.1725, found 469.1733.

#### (2S*,4R*,6S*)-2-Benzyl-4-chloro-4-(dimethyl(phenyl)silyl)-6-(4-chlorophenyl)tetrahydro-2*H*-pyran (**2p**)

Faint yellow oil. According to the general procedure, the title compound **2p** was obtained from alcohol **1b** (148 mg, 0.500 mmol) and 4-chlorobenzaldehyde to give, after column chromatography (hexane/EtOAc: 30:1), a yellow oil (189 mg, 83%). ^1^H NMR (500 MHz, CDCl_3_) δ 7.47–7.25 (m, 10H), 7.17–7.13 (m, 4H), 4.15 (dd, *J* = 12.0, 2.0 Hz, 1H), 3.58–3.51 (m, 1H), 2.94 (dd, *J* = 13.3, 5.2 Hz, 1H), 2.62 (dd, *J* = 13.3, 8.0 Hz, 1H), 2.48 (dt, *J* = 13.5, 2.0 Hz, 1H), 2.35 (dt, *J* = 13.4, 2.0 Hz, 1H), 2.01 (dd, *J* = 13.5, 12.0 Hz, 1H), 1.85 (dd, *J* = 13.4, 11.7 Hz, 1H), 0.53 (s, 3H, CH_3_), 0.42 (s, 3H, CH_3_). ^13^C {H} NMR (101 MHz, CDCl_3_) δ 140.4, 137.5, 136.8, 134.7, 133.2, 130.0, 129.5, 128.5, 128.3, 128.2, 127.0, 126.5, 76.4, 76.3, 60.6, 47.2, 43.9, 42.7, −3.3, −4.1. HRMS (ESI^+^) *m/z* calcd for C_26_H_28_Cl_2_NaOSi ([M + Na]^+^): 477.1179, found 477.1175.

### Synthesis of Compound **4** by the Stereoselective Desilylation Process

Over a suspension of 1.734 g TBAF (15% in alumina, 0.996 mmol, 3 equiv) in 6 mL THF, at room temperature and open to air, a solution of 123 mg (0.332 mmol, 1 equiv) of **2h** in 0.4 mL THF is added dropwise, the system is closed, and the mixture is stirred overnight. The reaction is quenched with 20 mL of water, layers are separated and the aqueous layer is extracted 3 times with diethyl ether. The combined organic extracts are washed with brine, dried over MgSO_4_, filtered, and evaporated under reduced pressure. After purification by flash column chromatography (hexane-acetate 20:1) 68 mg (0.287 mmol, 87%) of compound **4** are obtained as a yellowish oil.

#### (2R*,4S*,6R*)-4-Chloro-2-methyl-6-((*E*)-styryl)tetrahydro-2*H*-pyran

The spectroscopic data are in accordance with those reported in the literature.^27^^1^H NMR (400 MHz, CDCl_3_) δ 7.40–7.33 (m, 2H), 7.32–7.26 (m, 2H), 7.25–7.19 (m, 1H), 6.60 (d, *J* = 16.0 Hz, 1H), 6.18 (dd, *J* = 16.0, 6.2 Hz, 1H), 4.07 (tt, *J* = 11.8, 4.5 Hz, 1H), 4.04–3.97 (m, 1H, overlapped), 3.62–3.51 (m, 1H), 2.31–2.21 (m, 1H), 2.20–2.10 (m, 1H), 1.71 (dd, *J* = 13.6, 11.8 Hz, 1H), 1.56 (dd, *J* = 13.5, 11.8 Hz, 1H), 1.28 (d, *J* = 6.2 Hz, 3H). ^13^C {H} NMR (101 MHz, CDCl_3_) δ 136.5, 131.1, 128.7, 128.5, 127.7, 126.5, 77.1, 72.8, 55.3, 43.8, 42.2, 21.5. HRMS (ESI^+^) *m/z* calcd for C_14_H_17_ClKO ([M + K]^+^): 275.0605, found 275.0817.

### Synthesis of Antinociceptive Compound **5**

#### Synthesis of Tetrahydropyran **2q**

To a suspension of BiCl_3_ (59 mg, 0.187 mmol, 0.05 equiv) in 30 mL of dichloromethane, 1-naphthaldehyde (4.52 mmol, 1.2 equiv) is added. Then the suspension is cooled to 0 °C and TMSCl (0.95 mL, 7.54 mmol, 1.2 equiv) is added dropwise. The mixture is stirred for 5 min and a solution of 1.049 g of alcohol **1f** (3.77 mmol, 1 equiv) in 4 mL of dichloromethane is added dropwise and the reaction is followed by TLC. When starting materials are consumed, the reaction is quenched with NaOH (aq) 2 M solution. The aqueous layer is extracted three times with dichloromethane. Combined organic extracts are washed with brine, dried over MgSO_4_, and filtered and volatiles are evaporated under reduced pressure. The crude mixture is purified by column chromatography (hexane/ethyl acetate 10:1) yielding 1.02 g (2.25 mmol, 60%) of compound **2q** as a yellow oil.

#### Ethyl (2S*,4R*,6R*)-4-Chloro-4-(dimethyl(phenyl)silyl)-6-(naphthalen-1-yl)tetrahydro-2*H*-pyran-2-carboxylate (**2q**)

^1^H NMR (400 MHz, CDCl_3_) δ 7.86–7.60 (m, 6H), 7.51–7.42 (m, 6H), 4.94 (d, *J* = 11.8 Hz, 1H), 4.28–4.17 (m, 3H), 2.86 (d, *J* = 13.5 Hz, 1H), 2.73 (d, *J* = 13.9 Hz, 1H), 2.38–2.28 (m, 2H), 1.31 (t, *J* = 7.1 Hz, 3H, CH_3_), 0.71 (s, 3H, CH_3_Si) 0.68 (s, 3H, CH_3_Si). ^13^C {H} NMR (101 MHz, CDCl_3_) δ 169.9, 136.5, 134.8, 133.6, 130.4, 130.1, 128.9, 128.5, 128.3, 128.1, 125.8, 125.6, 125.4, 123.7, 122.9, 74.6, 73.9, 61.3, 59.7, 45.6, 41.3, 14.2, −3.4, −3.7. HRMS (ESI^+^) *m/z* calcd for C_26_H_30_ClO_3_Si ([M + H]^+^): 453.1647, found 453.1655.

#### Synthesis of Tetrahydropyran **6**

Over a suspension of 105 mg of LiAlH_4_ (2.775 mmol, 3.0 equiv) in 8.8 mL of dry ethyl ether at 0 °C, a solution of **2q** (419 mg, 0.925 mmol, 1.0 equiv) in 0.5 mL dry ethyl ether is added dropwise. When starting material is consumed (typically 1 h) the reaction is quenched with HCl 1 M. Layers are separated, and the aqueous layer is extracted 3 times with diethyl ether. The combined organic extracts are washed with brine, dried over MgSO4, filtered, and evaporated under reduced pressure. After purification by flash column chromatography (hexane-acetate 4:1) compound **6** was isolated as a colorless oil (304 mg, 0.74 mmol, 80%).

#### ((2S*,4R*,6R*)-4-Chloro-4-(dimethylphenylsilyl)-6-(naphthalen-1-yl)tetrahydro-2*H*-pyran-2-yl)methanol (**6**)

Colorless oil. ^1^H NMR (500 MHz, CDCl_3_) δ 7.87–7.67 (m, 5H), 7.58–7.55 (m, 1H), 7.50–7.42 (m, 6H), 4.99 (dd, *J* = 12.0, 1.9 Hz, 1H), 3.77–3.72 (m, 1H), 3.66–3.54 (m, 2H), 2.74 (dt, *J* = 13.7, 1.9 Hz, 1H), 2.46–2.36 (m, 2H), 2.08 (dd, *J* = 13.5, 12.1 Hz, 1H), 1.92 (dd, *J* = 8.5, 4.6 Hz, 1H, OH), 0.68 (s, 3H, CH_3_-Si), 0.66 (s, 3H, CH_3_-Si). ^13^C {H} NMR (101 MHz, CDCl_3_) δ 136.7, 136.5, 134.9, 133.7, 130.5, 130.1, 128.9, 128.5, 128.3, 126.0, 125.5, 125.4, 123.3, 123.0, 76.2, 73.5, 66.1, 60.7, 45.4, 40.7, −3.2, −3.3. HRMS (ESI^+^) *m/z* calcd for C_24_H_27_ClNaO_2_Si ([M + Na]^+^): 433.1361, found 433.1366.

#### Synthesis of Antinociceptive Compound **5**

Over a suspension of 3.05 g TBAF (15% in alumina, 1.75 mmol, 4 equiv) in 1.5 mL THF, at room temperature and open to air, a solution of 180 mg (0.438 mmol, 1 equiv) of **6** in 0.3 mL THF is added dropwise, the system is closed, and the mixture is stirred overnight. The reaction is quenched with 20 mL of water, layers are separated and the aqueous layer is extracted 3 times with diethyl ether. The combined organic extracts are washed with brine, dried over MgSO_4_, filtered, and evaporated under reduced pressure. After purification by flash column chromatography (hexane-acetate 2:1), 64 mg (0.232 mmol, 53%) of compound **5** are obtained as a yellowish solid (mp: 80.1–82.6 °C).

#### ((2S*,4R*,6R*)-4-Chloro-6-(naphthalen-1-yl)tetrahydro-2*H*-pyran-2-yl)methanol (**5**)

The spectroscopic data are in accordance with the literature.^26,28^^1^H NMR (400 MHz, CDCl_3_) δ 8.02–7.98 (m, 1H), 7.90–7.80 (m, 2H), 7.64–7.60 (m, 1H), 7.57–7.46 (m, 3H), 5.15 (dd, *J* = 11.3, 1.9 Hz, 1H), 4.35 (tt, *J* = 11.8, 4.5 Hz, 1H, CH-Cl), 3.91–3.82 (m, 1H), 3.79–3.67 (m, 2H), 2.65–2.57 (m, 1H), 2.29–2.22 (m, 1H), 2.11 (dt, *J* = 13.1, 11.6 Hz, 1H), 2.06–2.03 (m, 1H, OH), 1.84 (dt, *J* = 12.8, 11.6 Hz, 1H). ^13^C {H} NMR (101 MHz, CDCl_3_) δ 136.1, 133.8, 130.4, 129.0, 128.6, 126.3, 125.7, 125.4, 123.3, 122.9, 78.0, 75.7, 65.7, 55.4, 42.9, 38.1. HRMS (ESI^+^) *m/z* calcd for C_16_H_17_ClNaO_2_ ([M + Na]^+^): 299.0809, found 299.0818.

## Data Availability

The data underlying this study are available in the published article and its Supporting Information.

## Supporting Information Available

The synthetic procedure to prepare starting vinylsilyl alcohols and copies of NMR spectra (1D and 2D) for new compounds (PDF)
