# Supplementary material for: Changing the Reaction Pathway of Silyl-Prins Cyclization by Switching the Lewis Acid: Application to the Synthesis of an Antinociceptive Compound
Source: J Org Chem. 2023 May 23;88(11):6776–83. doi: 10.1021/acs.joc.3c00050 (PMC10242753; doi:10.1021/acs.joc.3c00050)

Supporting Information

Changing the reaction pathway of Silyl-Prins cyclization by switching the Lewis Acid: application to the synthesis of an antinociceptive compound.

Carlos Díez-Poza, Laura Fernández-Peña, Paula González-Andrés and Asunción Barbero*

Department of Organic Chemistry, Faculty of Science, University of Valladolid, Campus Miguel Delibes, 47011, Valladolid, SPAIN.
E-mail: asuncion.barbero@uva.es

Web: http://organosilanesorganicsynthesis.blogs.uva.es/

Table of Contents

1. Synthesis of the starting vinylsilyl alcohols **1a-f** S-1 to S-3

2. Copies of NMR Spectra S-4 to S-66

1. Synthesis of vinylsilyl alcohols 1a-f

The synthesis of the starting vinylsilyl alcohols was achieved in three high yielding steps from commercial reagents:

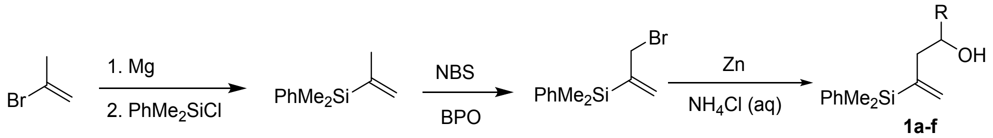

1.1. Synthesis of starting 2-dimethylphenylsilyl-prop-1-ene by Grignard reaction

To a suspension of 10.7 g of magnesium turnings (0.44 mol, 1.1 equiv) in 40 mL of dry THF at 50°C, 1.78 mL of 2-bromopropene (20 mmol, 0.05 equiv) are added under nitrogen atmosphere to start the reaction. After 5 min, the remaining 2-bromopropene (33.8 mL, 0.38 mol, 0.94 equiv) dissolved in 100 mL of dry THF is added dropwise through an addition funnel at a rate sufficient to maintain reflux (heating is turned off as it is an exothermic reaction). The mixture is heated again, and after 30 min, heating is turned off and phenyldimethylchlorosilane (66.3 mL, 0.4 mol, 1 equiv) is added. The mixture is then heated again to reflux during 90 min, after which it is cooled down to room temperature, placed in an ice-water bath, and slowly hydrolized with an ice-cold saturated aqueous solution of NH_4Cl. The crude mixture is then extracted three times with diethyl ether, organic extracts are washed with saturated aqueous NaCl, dried over MgSO4, filtered, and solvents eliminated under reduced pressure to afford ompound xx as a yellow oil (69.8 g, 396 mmol, 99%). Spectroscopic data (**^{1}H NMR** (400 MHz, $CDCl_3$) δ 7.54 – 7.50 (m, 2H), 7.39 – 7.33 (m, 3H), 5.68 (br s, 1H), 5.34 (br s, 1H), 1.82 (s, 3H), 0.37 (s, 6H). **^{13}C {^{1}H} NMR** (101 MHz, $CDCl_3$) δ 146.1, 138.0, 133.9, 128.9, 127.7, 126.5, 22.5, -3.5) are in accordance with the literature.[1]

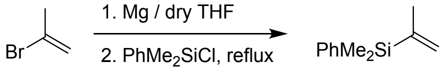

1.2. Bromination of the vinylsilane with N-bromosuccinimide

N-bromo-succinimide (3.68 g, 20.7 mmol, 0.9 equiv) and benzoyl peroxide (0.371 g, 1.15 mmol, 0.05 equiv) are added to a two-necked flask containing 40 mL of carbon tetrachloride. Then, to the suspension formed, vinylsilane xx (4.067 g, 23 mmol) is added and the mixture is heated to reflux and stirred until consumption of the starting material (8 to 12 hours). After that time, the reaction is let to cool down to room temperature, filtered, and evaporated under reduced pressure. The crude mixture is purified by column chromatography (hexane) and compound xx is obtained as a yellow oil ((2.50 g, 9.80 mmol, 47%). Spectroscopic data (**^{1}H NMR** (400 MHz, $CDCl_3$) δ 7.55 – 7.50 (m, 2H), 7.40 – 7.33 (m, 3H), 6.03 (br s, 1H), 5.57 (br s, 1H), 4.09 (br s, 2H), 0.47 (s, 6H). **^{13}C {^{1}H} NMR** (101 MHz, $CDCl_3$) δ 145.7, 136.8, 133.9, 131.0, 129.4, 127.9, 37.4, -2.7) are in accordance with those in the literature.[1]

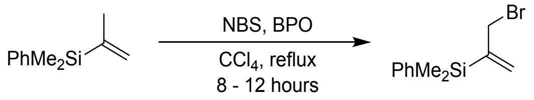

1.3. Synthesis of vinylsilyl alcohols by Barbier reaction

Method A (compounds **1a**-**e**): The corresponding commercial aldehyde (1.5 equiv. for acetaldehyde, 0.6 equiv. for other aldehydes) is added over a suspension of zinc (3 mmol, 1.5 equiv.) in 2 mL of a saturated aqueous NH_4Cl solution. Then, a solution of (3-bromoprop-1-en-2-yl)dimethyl(phenyl)silane (2 mmol, 1 equiv.) in 0.4 mL THF is added dropwise (the temperature of the mixture rises considerably, due to exothermic reaction). The reaction is followed by TLC, and when starting materials are consumed (tipycally around 3 hours) is is hydrolized with HCl (aqueous, 1M). Phases are then separated, and the aqueous layer is extracted three times with diethyl ether. Organic extracts are combined and dried over magnesium sulfate, filtered, and evaporated under reduced pressure. After column chromatography (hexane – ethyl acetate 10:1) compounds **1a**-**e** are obtained.

[1] Fleming, I.; Rowley, M.; Cuadrado, P.; González-Nogal, A.M.; Pulido, F.J. *Tetrahedron*, **1989**, *45*, 413–424.

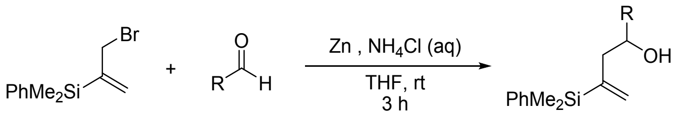

1a-e

Method B (compound **1f**): over a suspension of 133 mg of zinc dust (2.00 mmol, 1.5 equiv.) in 0.5 mL dry THF, 0.26 mL of TMSCl (2.0 mmol, 1.5 equiv.) are added under nitrogen atmosphere. Then, a mixture of 0.265 mL of a 50% ethyl glyoxylate (1.33 mmol, 1.0 equiv.) solution in toluene and a solution of 3-bromoprop-1-en-2-yl)dimethyl(phenyl)silane (1.33 mmol, 1.0 equiv.) in 0.7 mL dry THF is added dropwise (the temperature of the mixture rises considerably, due to exothermic reaction). The reaction is let to stir overnight and is then (ca. 18 hours) hydrolized with 2 mL of a saturated aqueous NH_4Cl solution. The crude mixture is extracted three times with diethyl ether, washed with saturated aqueous $NaHCO_3$, dried over $MgSO_4$, and evaporated under reduced pressure. Then, column chromatography (hexane – ethyl acetate 10:1) afforded 52 mg (0.187 mmol, 28%) of compound 1f as a yellow oil.

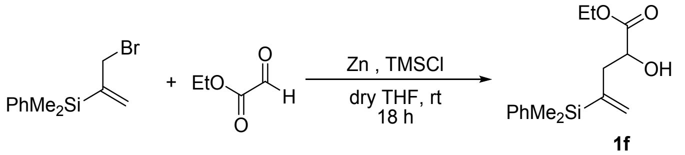

1f

Compound 1a: *4-(dimethyl(phenyl)silyl)pent-4-en-2-ol*

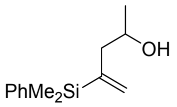

According to the general procedure (method A), the title compound **1a** was obtained from 3-bromoprop-1-en-2-yl)dimethyl(phenyl)silane (4.40 g, 17.29 mmol) and 1.5 equiv. acetaldehyde to give, after column chromatography (hexane/EtOAc: 10:1), a yellow oil (1.83 g, 96%). **^{1}H RMN** (500 MHz, $CDCl_3$) δ 7.52 – 7.49 (m, 2H), 7.38 – 7.35 (m, 3H), 5.79 (br s, 1H), 5.58 (br s, 1H), 3.75-3.67 (m, 1H), 2.36-2.31 (m, 1H), 2.19 (dd, *J* = 13.8, 9.9Hz, 1H), 1.58 (br s, 1H, OH), 1.11 (d, *J* = 6.2 Hz, 3H), 0.40 (s, 6H). **^{13}C {^{1}H} RMN** (101 MHz, $CDCl_3$) δ 147.6, 137.7, 133.8, 129.5, 129.2, 127.9, 65.9, 46.6, 22.8, -2.9, -3.0. **HRMS** (ESI+) *m/z* calcd for $C_{13}H_{20}NaOSi$ ($[M+Na]^+$): 243.1176, found 243.1173.

Compound 1b: *4-(dimethyl(phenyl)silyl)-1-phenylpent-4-en-2-ol*

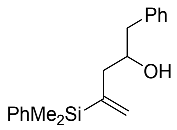

According to the general procedure (method A), the title compound **1b** was obtained from 3-bromoprop-1-en-2-yl)dimethyl(phenyl)silane (1.50 g, 5.91 mmol) and 0.6 equiv. phenylacetaldehyde to give, after column chromatography (hexane/EtOAc: 10:1), a yellow oil (397 mg, 46%). **^{1}H NMR** (400 MHz, $CDCl_3$) δ 7.51 – 7.46 (m, 2H), 7.40 – 7.32 (m, 3H), 7.30 – 7.23 (m, 2H), 7.23 – 7.17 (m, 1H), 7.08 - 7.03 (m, 2H), 5.84 – 5.79 (m, 1H), 5.58 (d, *J* = 2.9, 1H), 3.78 – 3.69 (m, 1H), 2.71 – 2.60 (m, 2H), 2.41 (dd, *J* = 13.8, 4.2 Hz, 1H), 2.26 (dd, *J* = 13.8, 8.7 Hz, 1H), 0.38 (s, 3H), 0.36 (s, 3H). **^{13}C {^{1}H} NMR** (101 MHz, $CDCl_3$) δ 147.5, 138.5, 137.8, 133.8, 129.5, 129.3, 129.2, 128.4, 127.9, 126.3, 70.7, 44.3, 43.4, -2.9, -3.0. **HRMS** (ESI+) *m/z* calcd for $C_{19}H_{24}NaOSi$ ($[M+Na]^+$): 319.1489, found 319.1488.

Compound 1c: *(E)-5-(dimethyl(phenyl)silyl)-1-phenylhexa-1,5-dien-3-ol*

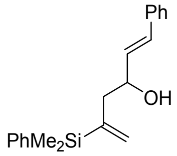

According to the general procedure (method A), the title compound **1c** was obtained from 3-bromoprop-1-en-2-yl)dimethyl(phenyl)silane (1.94 g, 7.63 mmol) and cinnamaldehyde to give, after column chromatography (hexane/EtOAc: 8:1), a yellow oil (727 mg, 62%). **^{1}H NMR** (400 MHz, $CDCl_3$) δ 7.63 – 7.12 (m, 10H), 6.44 (d, *J* = 15.9, 1H), 6.13 (dd, *J* = 15.8, 6.2, 1H), 5.87 – 5.85 (m, 1H), 5.68 – 5.57 (m, 1H), 4.24 – 4.17 (m, 1H), 2.51 (dd, *J* = 14.0, 4.4 Hz, 1H), 2.39 (dd, *J* = 14.0, 8.9 Hz, 1H), 1.75 (brs, 1H, OH), 0.43 (s, 3H), 0.42 (s, 3H). **^{13}C {^{1}H} NMR** (101 MHz, $CDCl_3$) δ 146.7, 137.6, 136.6, 133.8, 131.5, 130.0, 129.9, 129.2, 128.5, 127.9, 127.5, 126.4, 70.7, 44.8, -2.9, -3.0. **HRMS** (ESI+) *m/z* calcd for $C_{20}H_{24}NaOSi$ ($[M+Na]^+$): 331.1489, found 331.1493.

Compound 1d: *1-(4-chlorophenyl)-3-(dimethyl(phenyl)silyl)but-3-en-1-ol*

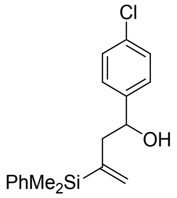

According to the general procedure (method A), the title compound **1d** was obtained from 3-bromoprop-1-en-2-yl)dimethyl(phenyl)silane (520 mg, 2.05 mmol) and 0.6 equiv. 4-chlorobenzaldehyde to give, after column chromatography (hexane/EtOAc: 10:1), a yellow oil (233 mg, 72%). **^{1}H NMR** (400 MHz, $CDCl_3$) δ 7.55 – 7.51 (m, 2H), 7.40 – 7.36 (m, 3H), 7.26 – 7.22 (m, 2H), 7.14 – 7.09 (m, 2H), 5.84 – 5.81 (m, 1H), 5.64 (d, *J* = 2.8 Hz, 1H), 4.49 (dt, *J* = 9.6, 2.8 Hz, 1H), 2.54 (ddd, *J* = 14.1, 3.7, 1.3 Hz, 1H), 2.38 (dd, *J* = 14.1, 9.6 Hz, 1H), 1.94 (d, *J* = 2.3 Hz, 1H, OH), 0.44 (s, 3H), 0.42 (s, 3H). **^{13}C {^{1}H} NMR** (101 MHz, $CDCl_3$) δ 147.2, 142.5, 137.5, 133.9, 132.9, 130.2, 129.3, 128.4, 128.0, 127.1, 71.5, 47.1, -2.9, -3.0. **HRMS** (ESI+) *m/z* calcd for $C_{18}H_{21}ClNaOSi$ ($[M+Na]^+$): 339.0942, found 339.0946.

Compound 1e: *3-(dimethyl(phenyl)silyl)-1-(naphthalen-1-yl)but-3-en-1-ol*

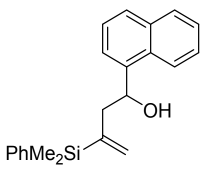

According to the general procedure (method A), the title compound **1e** was obtained from 3-bromoprop-1-en-2-yl)dimethyl(phenyl)silane (2.08 g, 6.47 mmol) and 0.6 equiv. 1-naphthaldehyde to give, after column chromatography (hexane/EtOAc: 8:1), a yellow oil (812 mg, 75%). **^{1}H NMR** (500 MHz, $CDCl_3$) δ 7.84 - 7.81 (m, 1H), 7.75 - 7.71 (m, 1H), 7.64 – 7.61 (m, 3H), 7.58 – 7.54 (m, 1H), 7.46 – 7.39 (m, 5H), 7.37 – 7.33 (m, 1H), 6.02 – 5.98 (m, 1H), 5.76 – 5.74 (m, 1H), 5.31 (d, *J* = 10.4 Hz, 1H), 2.87 – 2.82 (m, 1H), 2.51 (dd, *J* = 14.4, 10.3 Hz, 1H), 2.09 (brs, 1H, OH), 0.51 (s, 3H), 0.46 (s, 3H). **^{13}C {^{1}H} NMR** (101 MHz, $CDCl_3$) δ 148.0, 139.8, 137.6, 134.0, 133.7, 130.24, 130.18, 129.4, 128.8, 128.1, 127.7, 125.8, 125.5, 125.3, 122.9, 122.7, 68.7, 46.1, -2.6, -2.9. **HRMS** (ESI+) *m/z* calcd for $C_{22}H_{24}NaOSi$ ($[M+Na]^+$): 355.1498, found 355.1494.

Compound 1f: *ethyl 4-(dimethyl(phenyl)silyl)-2-hydroxypent-4-enoate*

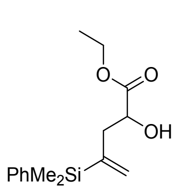

According to the general procedure (method B), the title compound **1f** was obtained from 3-bromoprop-1-en-2-yl)dimethyl(phenyl)silane (339 mg, 1.33 mmol) and 1 equiv. ethyl glyoxylate to give, after column chromatography (hexane/EtOAc: 10:1), a yellow oil (52 mg, 28%). **^{1}H NMR** (400 MHz, CDCl3) δ 7.55 – 7.50 (m, 2H), 7.38 – 7.33 (m, 3H), 5.86 – 5.84 (m, 1H), 5.59 – 5.57 (m, 1H), 4.19 – 4.11 (m, 1H), 4.18 (m, 2H), 2.67 (dddd, *J* = 14.6, 3.9, 1.6, 0.9 Hz, 1H), 2.39 (dd, *J* = 14.6, 8.5 Hz, 1H), 1.26 (t, *J* = 7.1, 3H), 0.413 (s, 3H), 0.409 (s, 3H). **^{13}C {^{1}H} NMR** (101 MHz, CDCl3) δ 174.6, 145.5, 137.7, 133.9, 129.7, 129.1, 127.8, 69.9, 61.5, 40.9, 14.2, -2.8, -3.0. **HRMS** (ESI+) *m/z* calcd for $C_{15}H_{22}NaO_3Si$ ($[M+Na]^+$): 301.1230, found 301.1228.

2. NMR spectra of compounds

^{1}H NMR (400 MHz, $CDCl_3$)

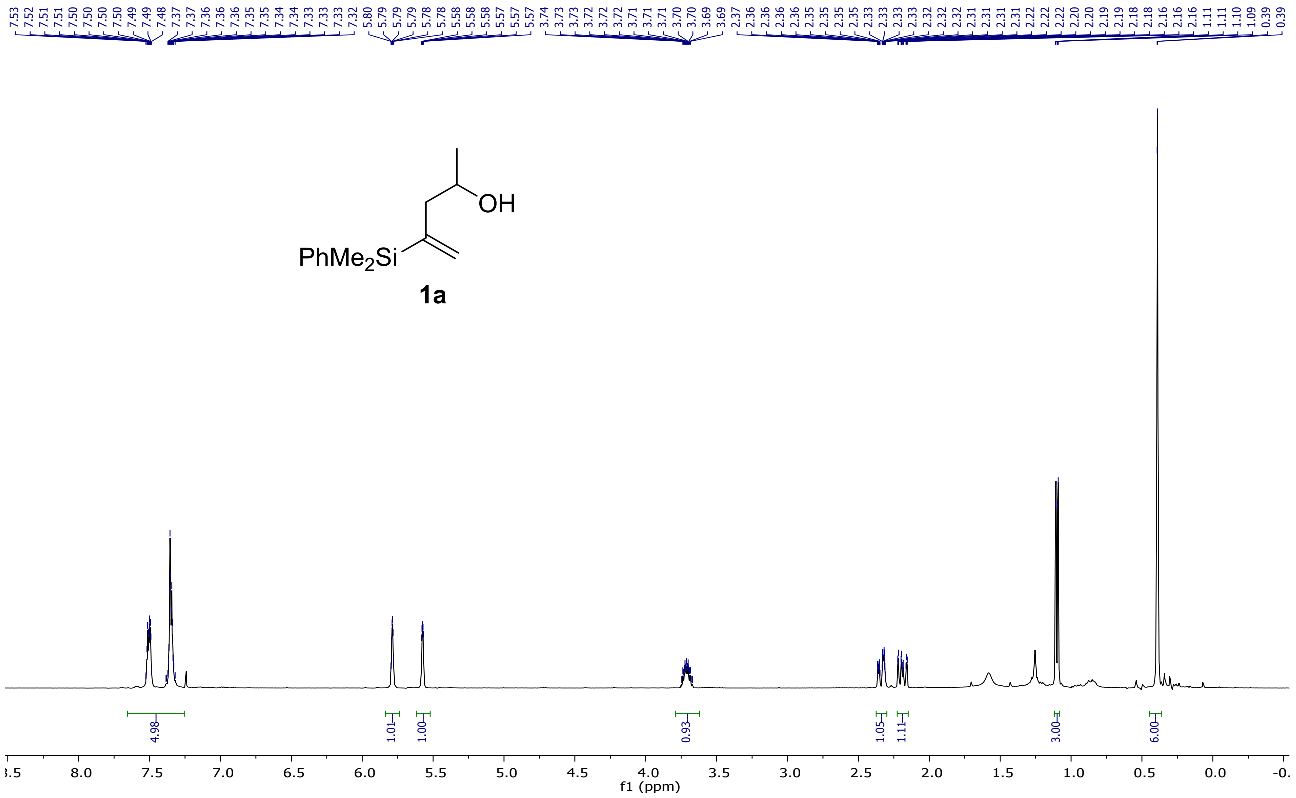

^{13}C {^{1}H} NMR (101 MHz, $CDCl_3$)

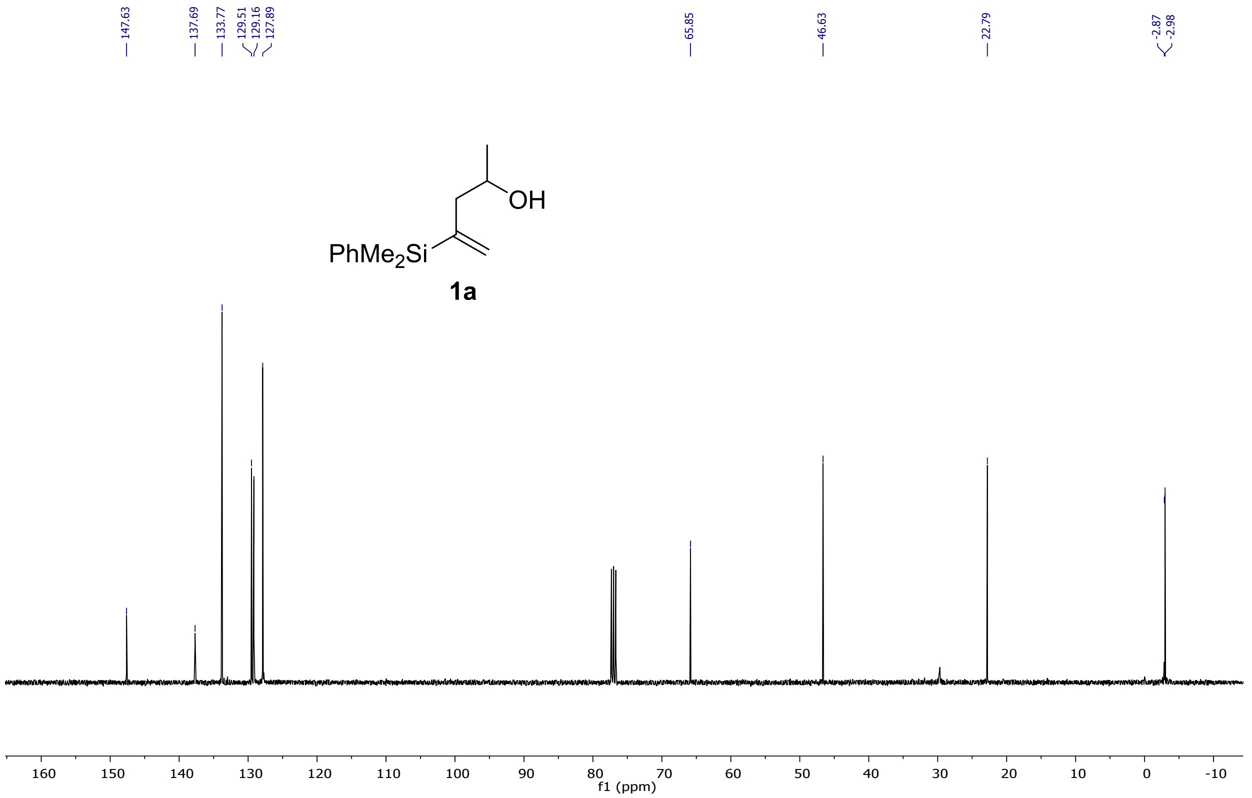

2D-COSY of compound 1a

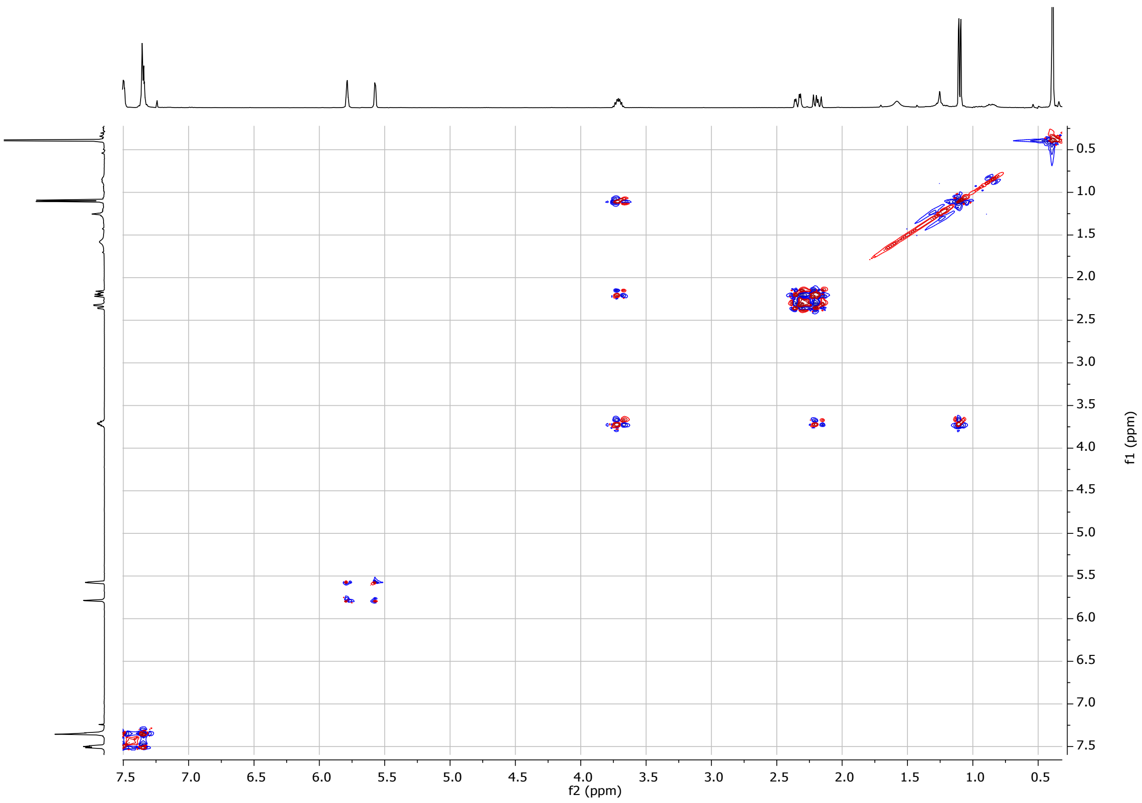

^{1}H NMR (400 MHz, CDCl$_3$)

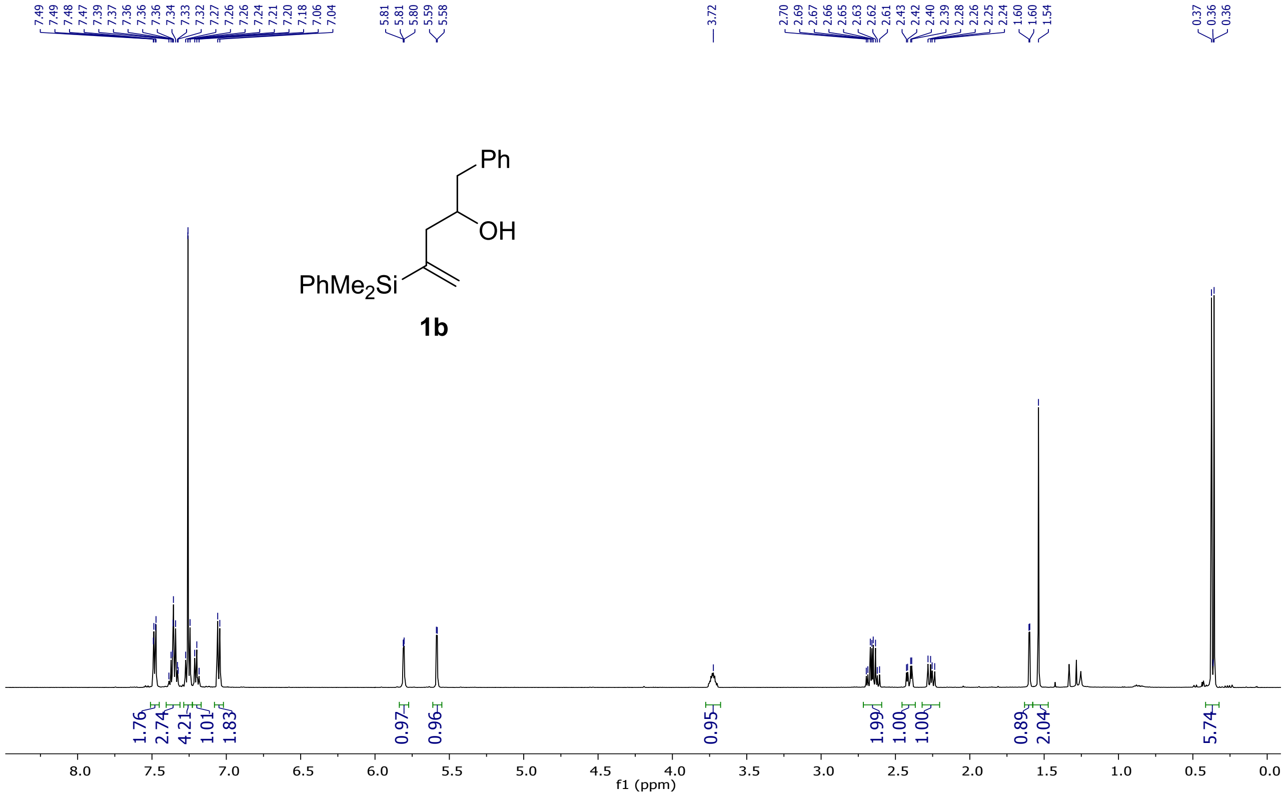

^{13}C {^{1}H} NMR (101 MHz, CDCl$_3$)

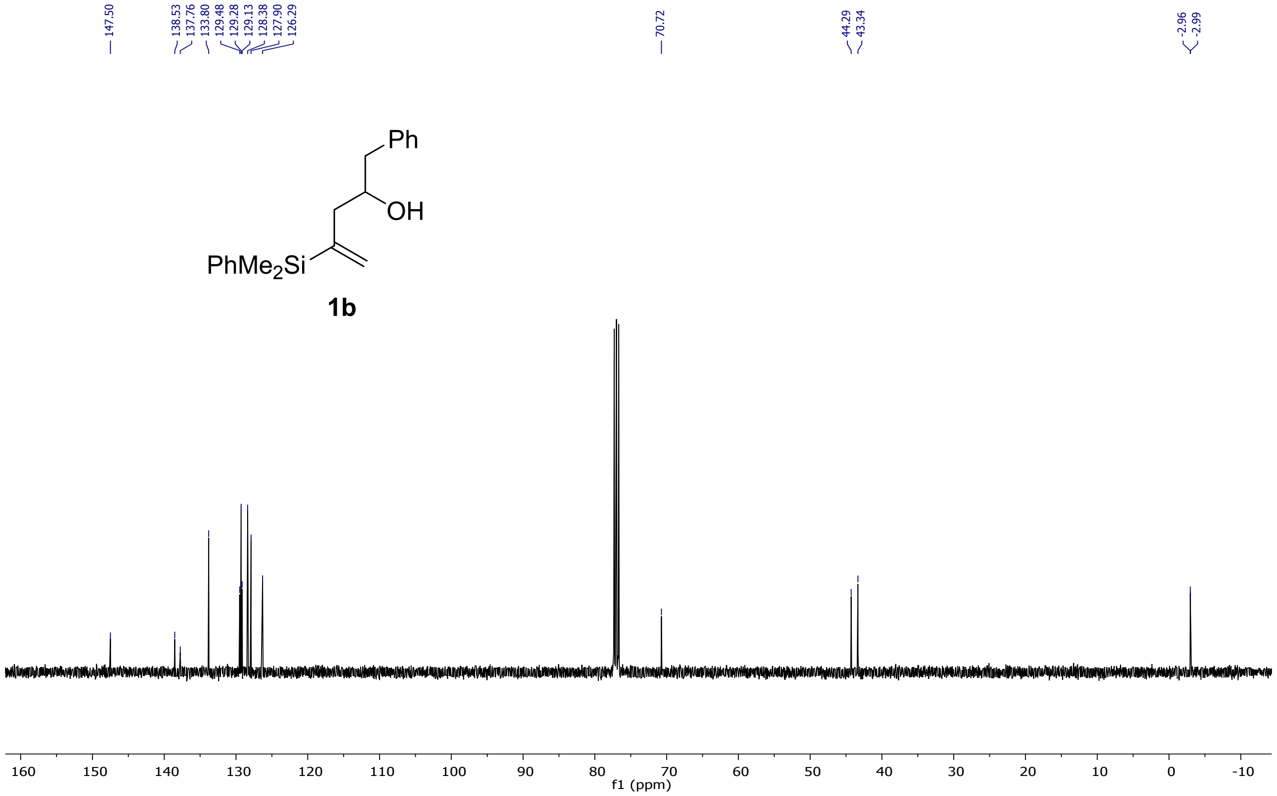

2D-COSY of compound 1b

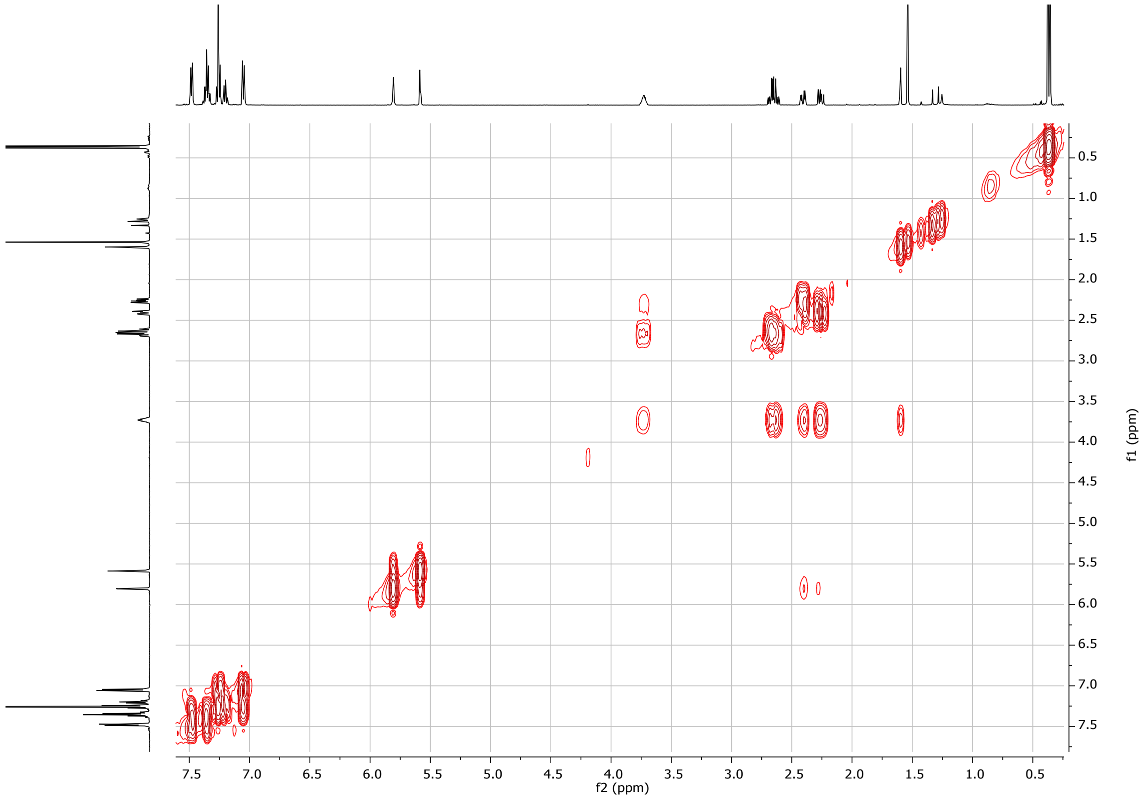

^{1}H NMR (400 MHz, $CDCl_3$)

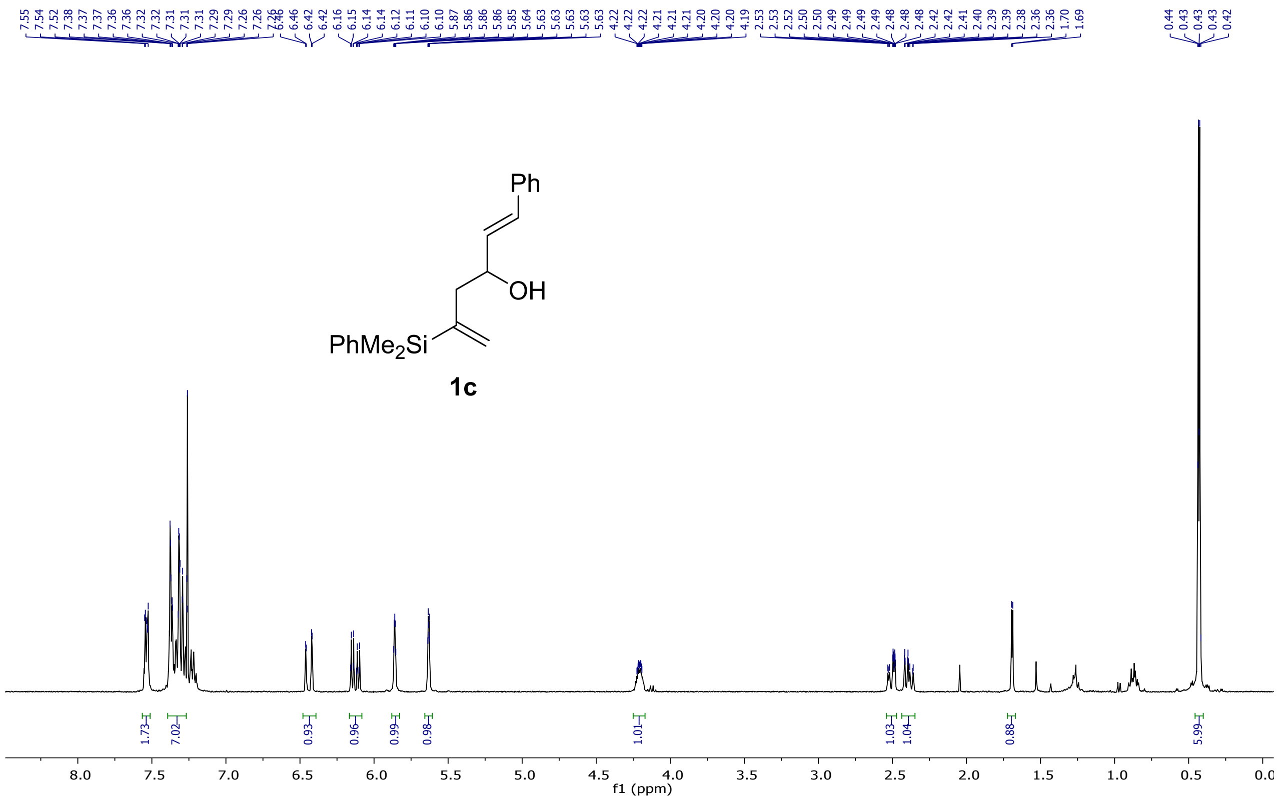

^{13}C {^{1}H} NMR (101 MHz, $CDCl_3$)

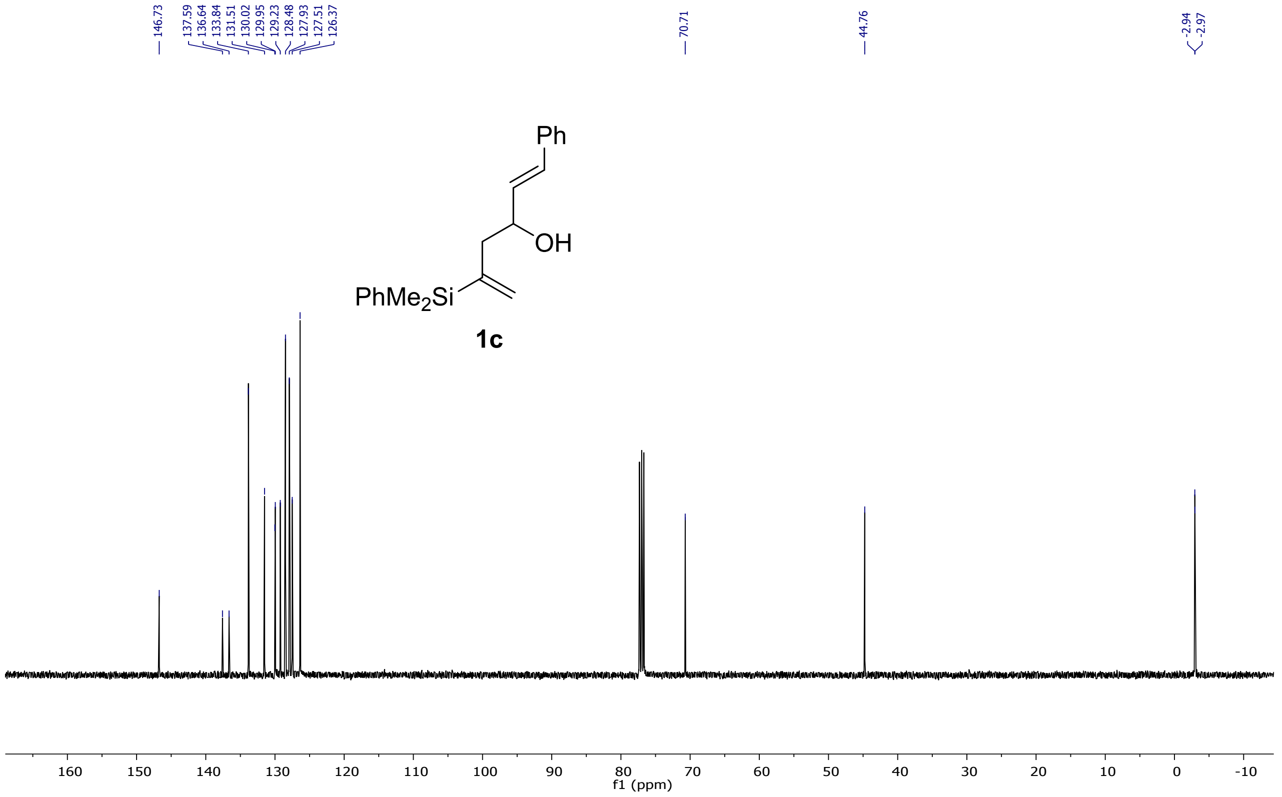

2D-COSY of compound 1c

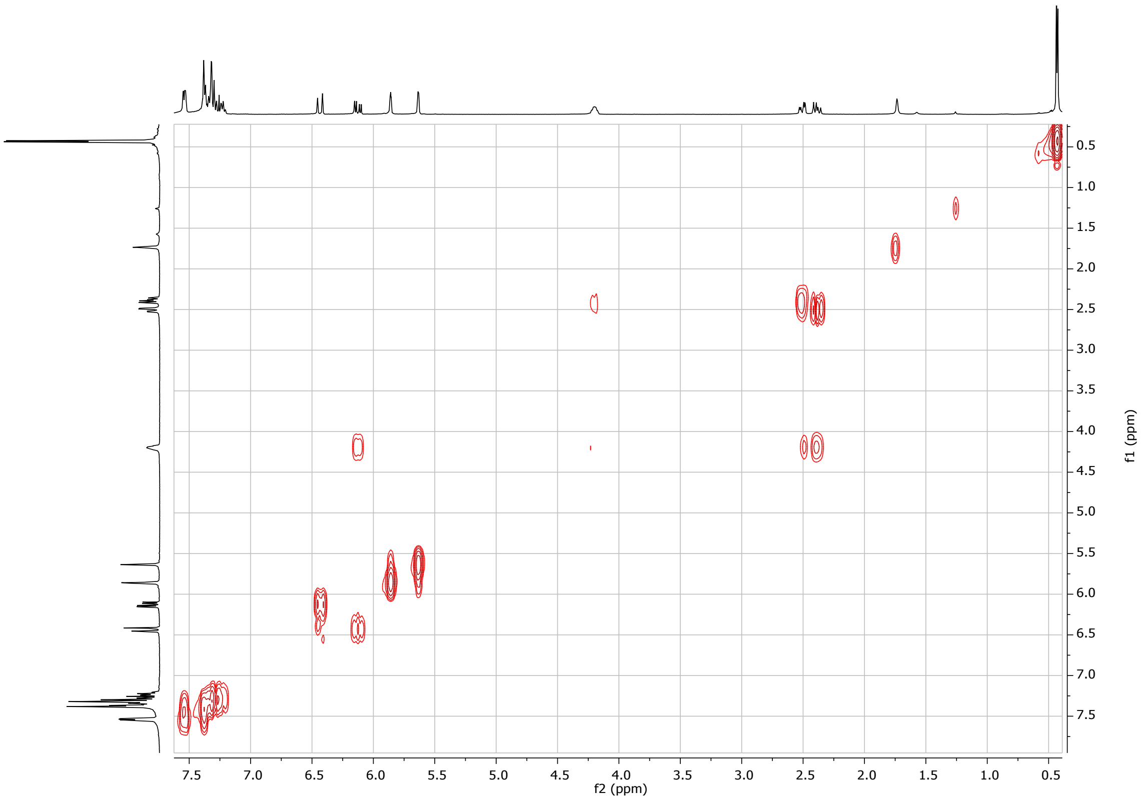

^{1}H NMR (400 MHz, $CDCl_3$)

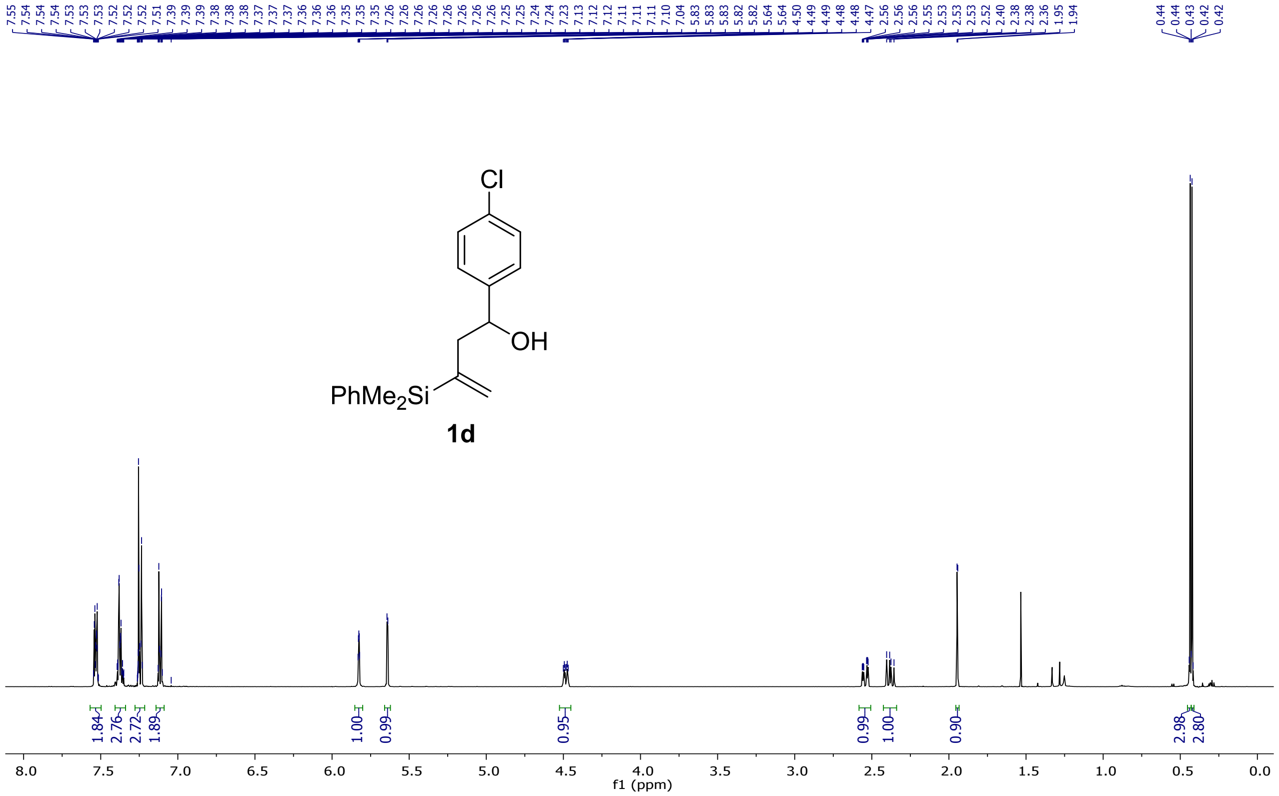

^{13}C {^{1}H} NMR (101 MHz, $CDCl_3$)

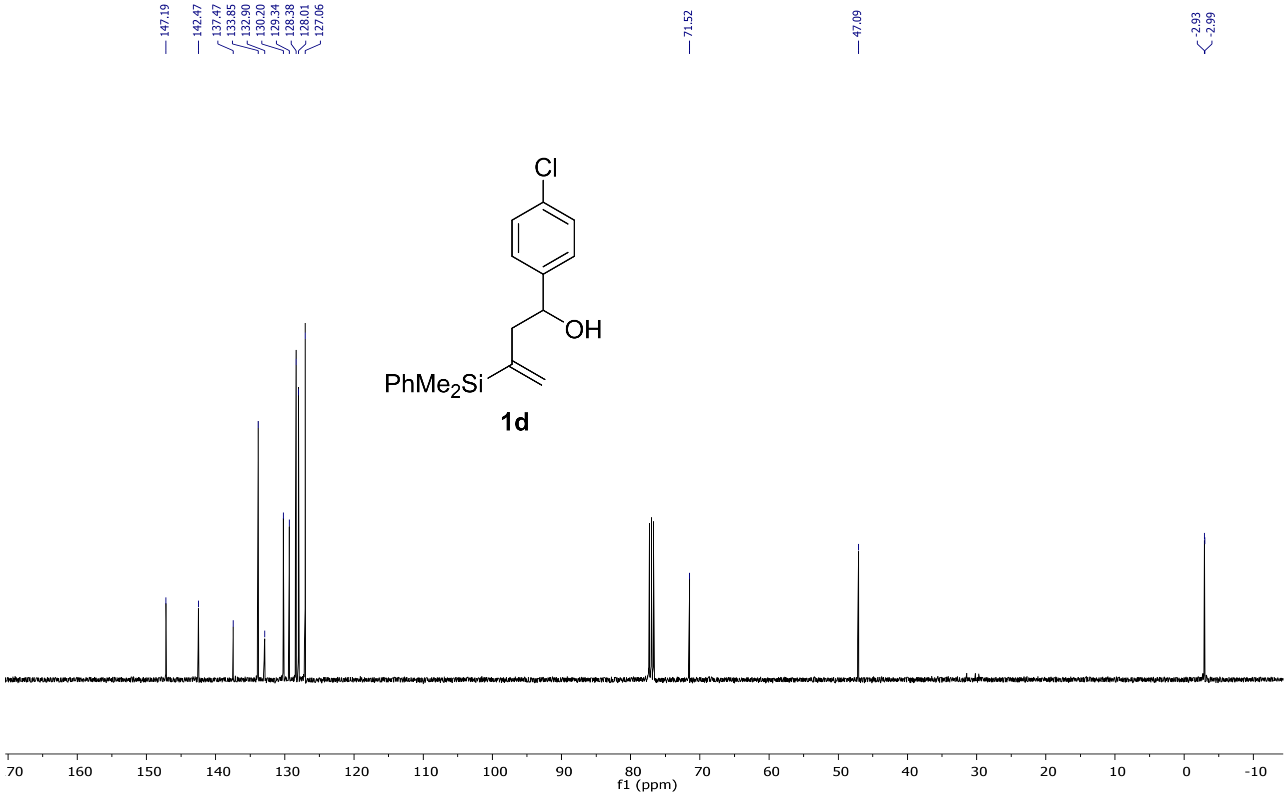

2D-COSY of compound 1d

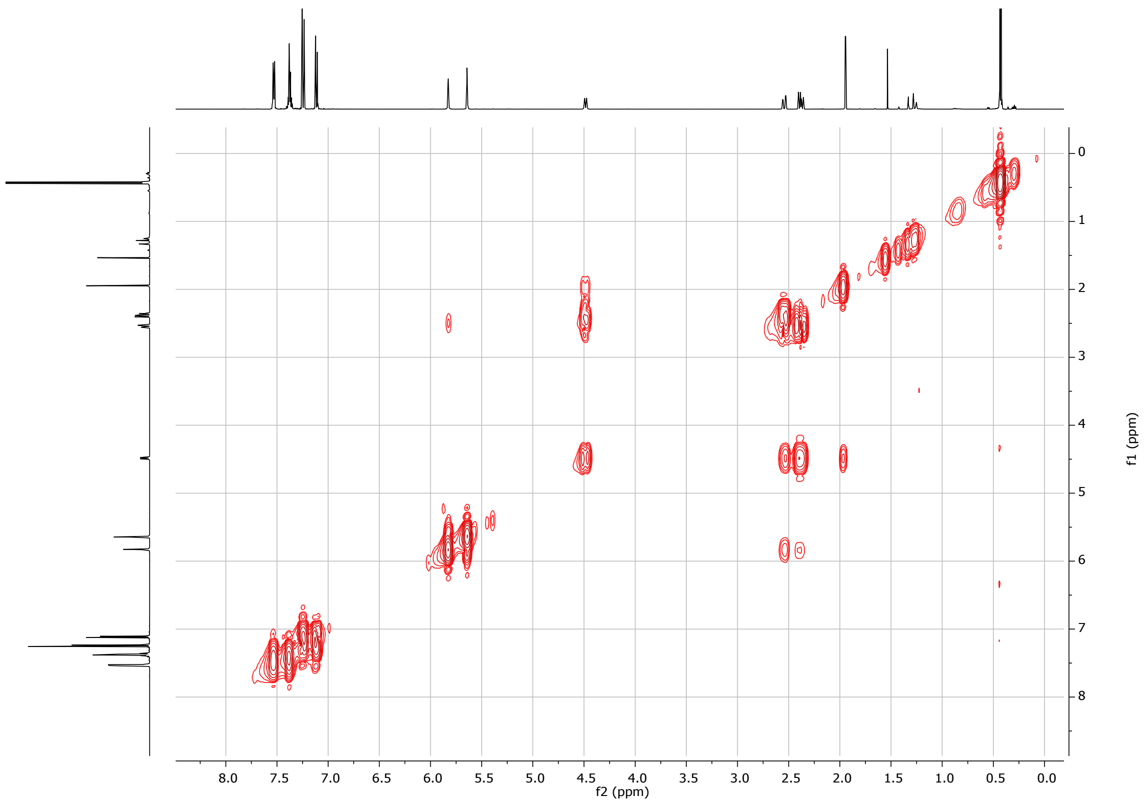

^{1}H NMR (400 MHz, $CDCl_3$)

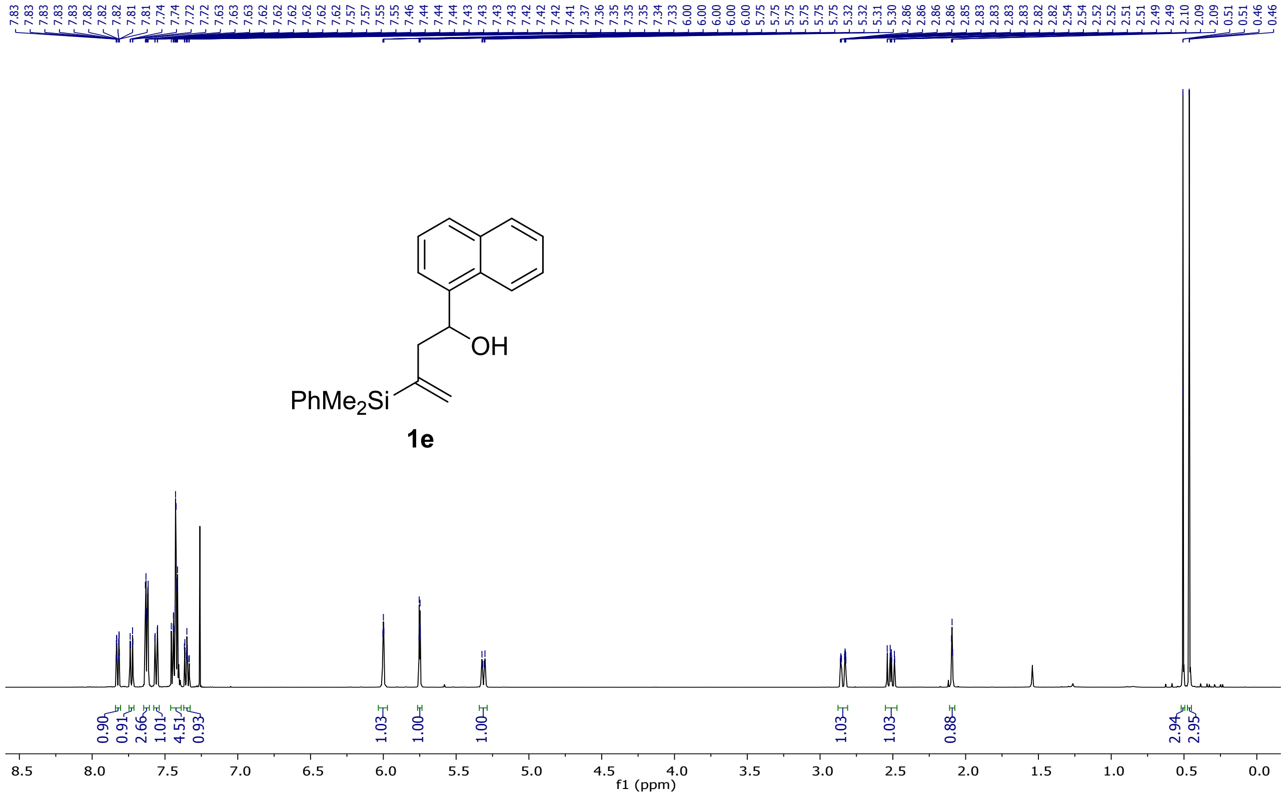

^{13}C {^{1}H} NMR (101 MHz, $CDCl_3$)

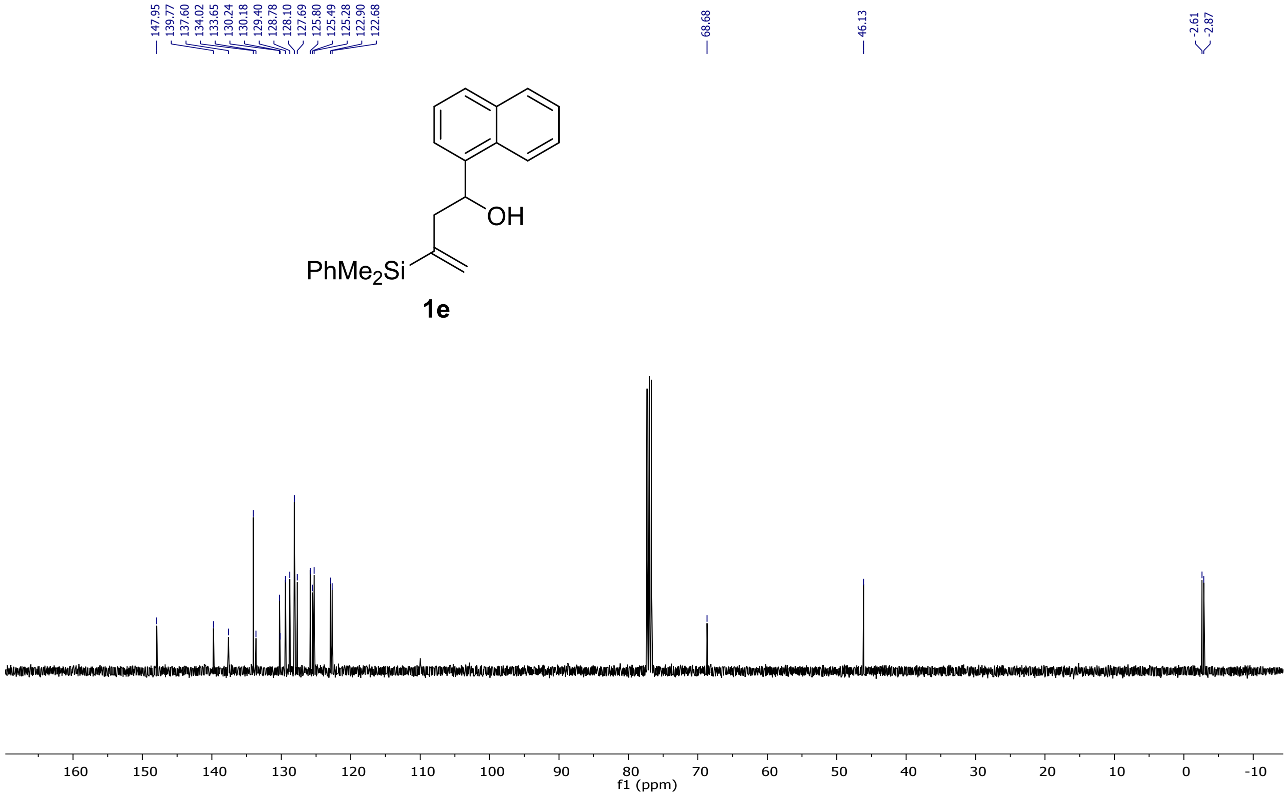

2D-COSY of compound 1e

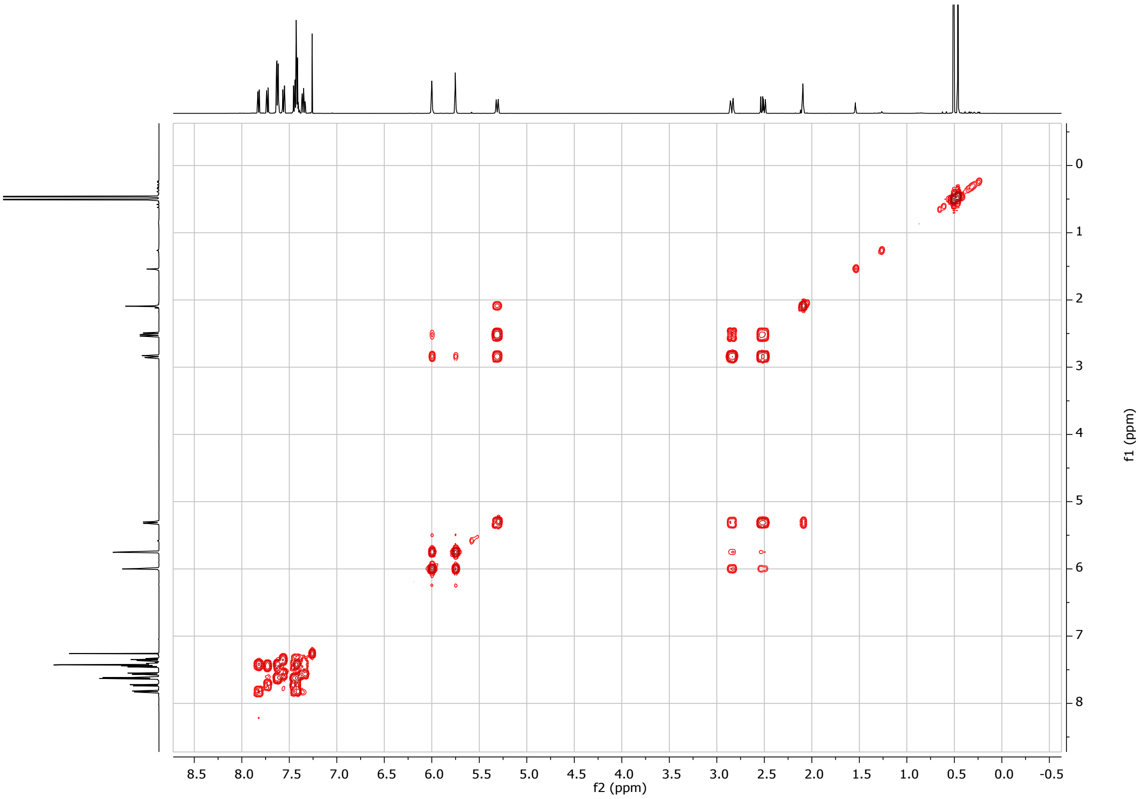

^{1}H NMR (400 MHz, $CDCl_3$)

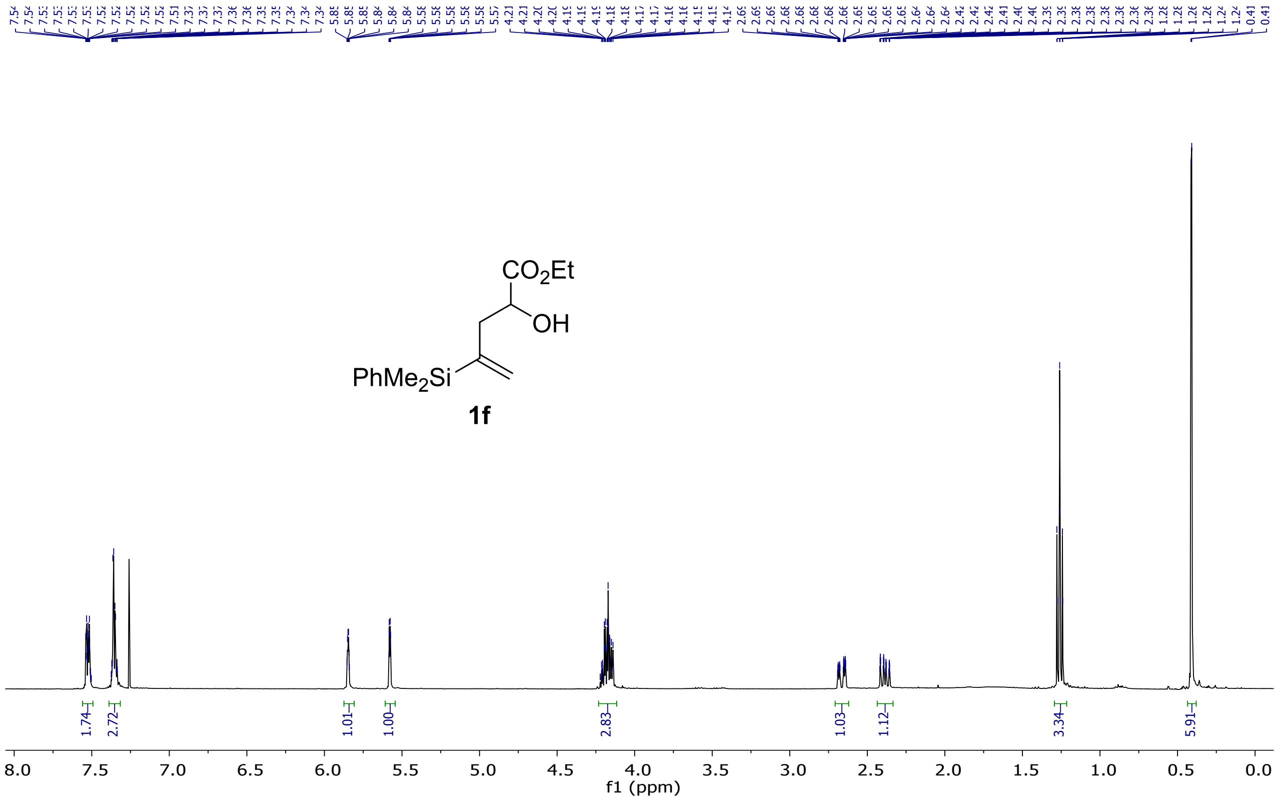

^{13}C {^{1}H} NMR (101 MHz, $CDCl_3$)

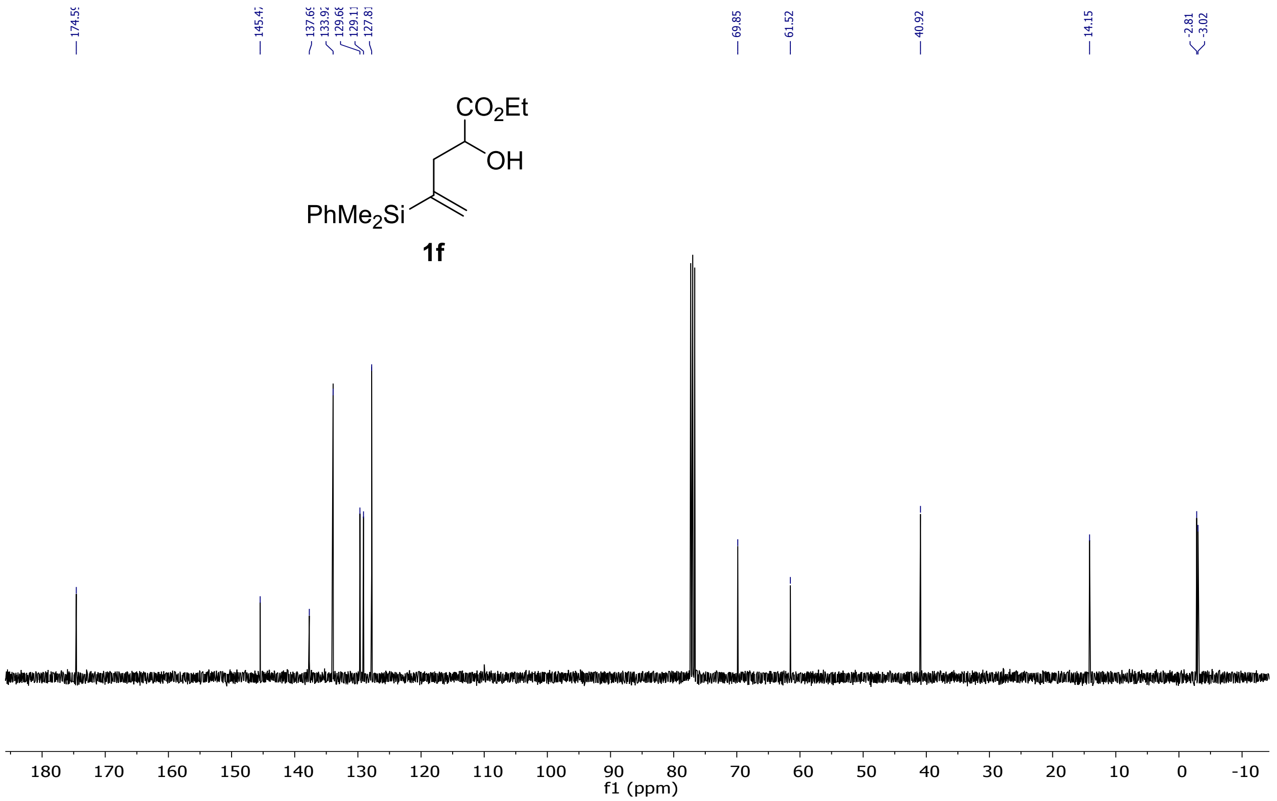

2D-COSY of compound 1f

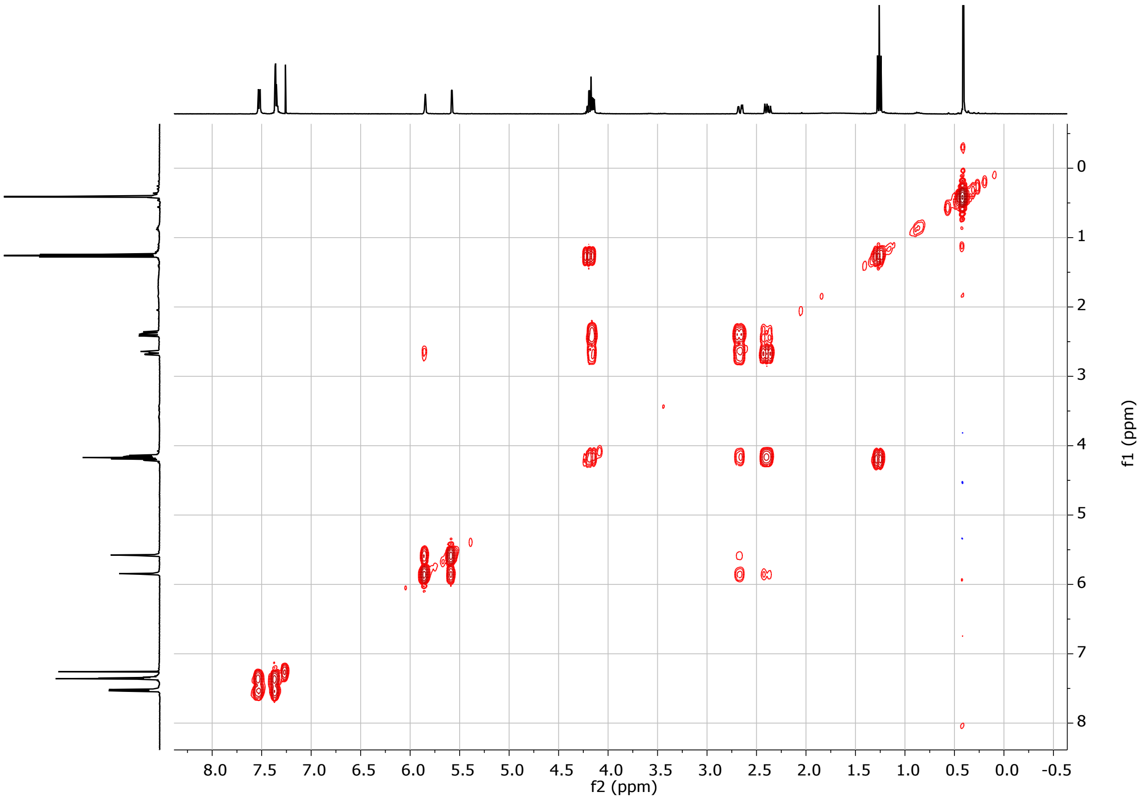

^{1}H NMR (400 MHz, $CDCl_3$)

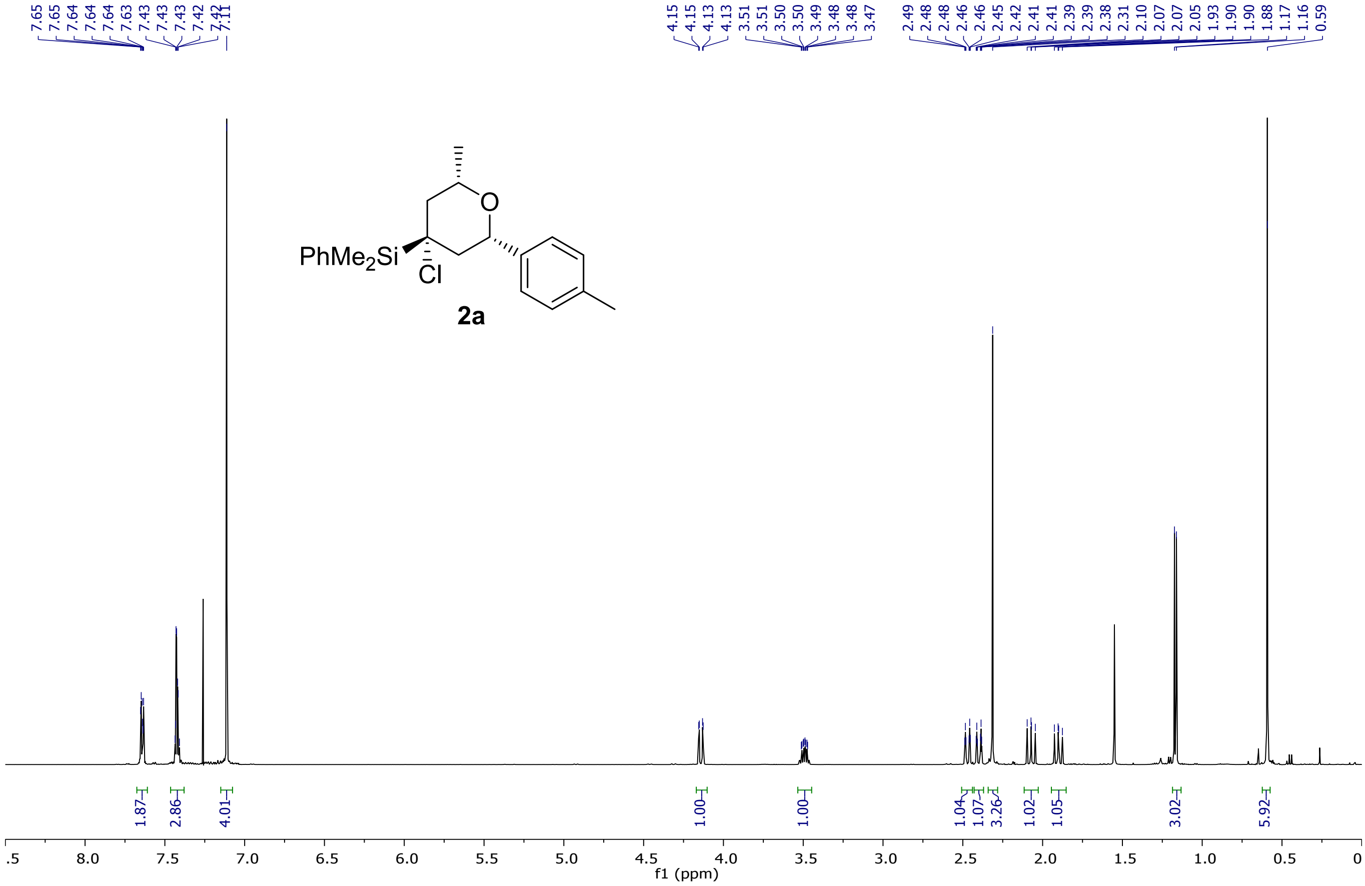

^{13}C {^{1}H} NMR (101 MHz, $CDCl_3$)

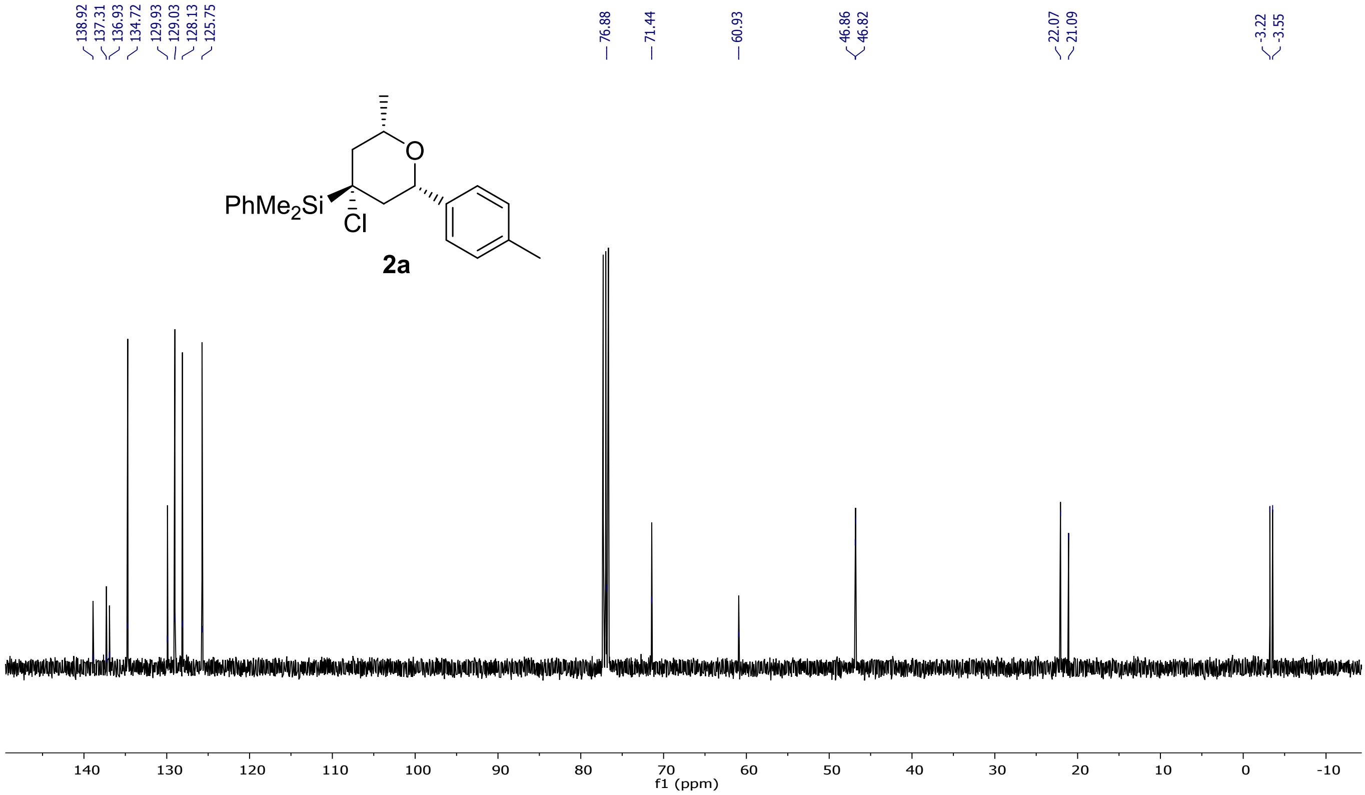

2D-COSY of compound 2a

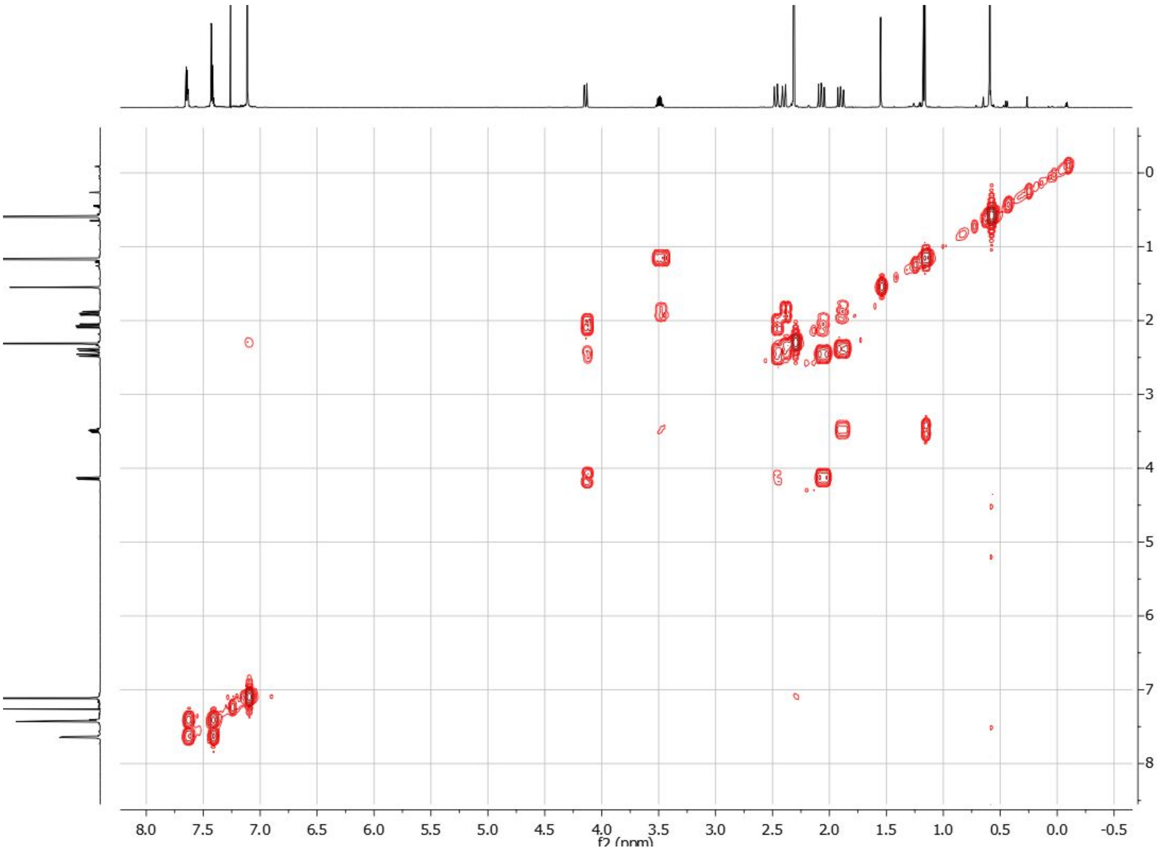

2D-HMBC of compound 2a

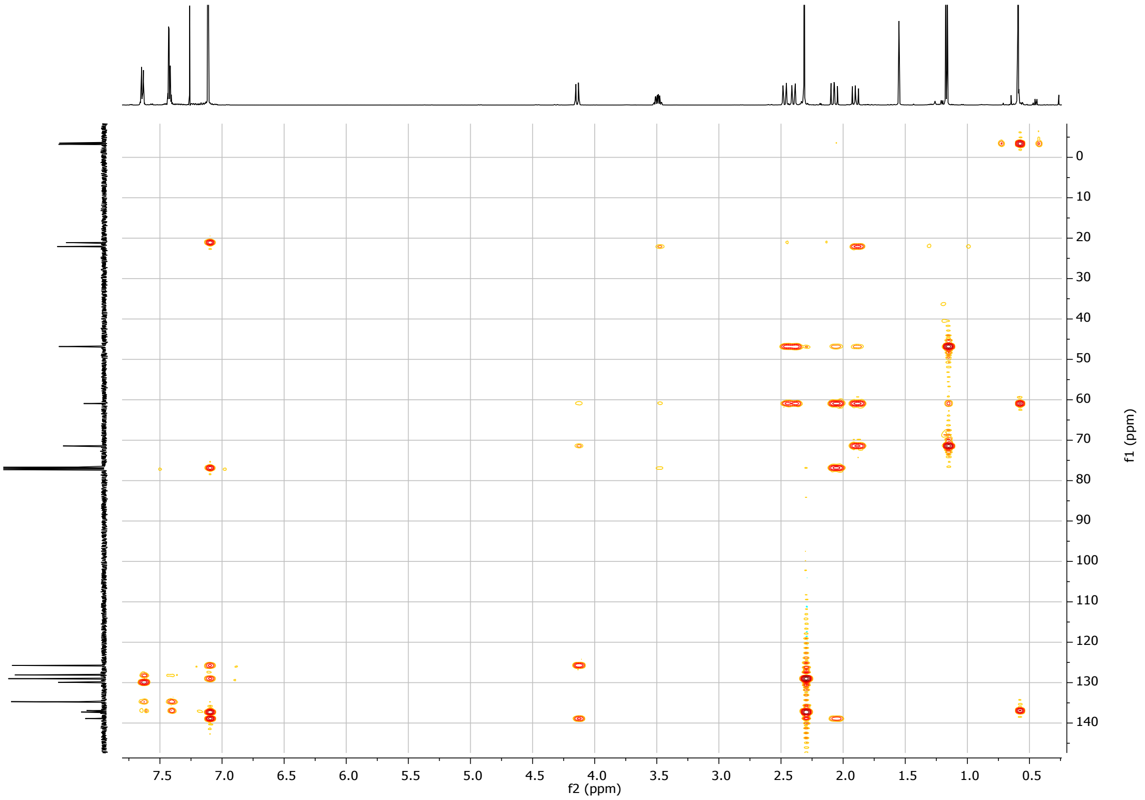

2D-NOESY of compound 2a

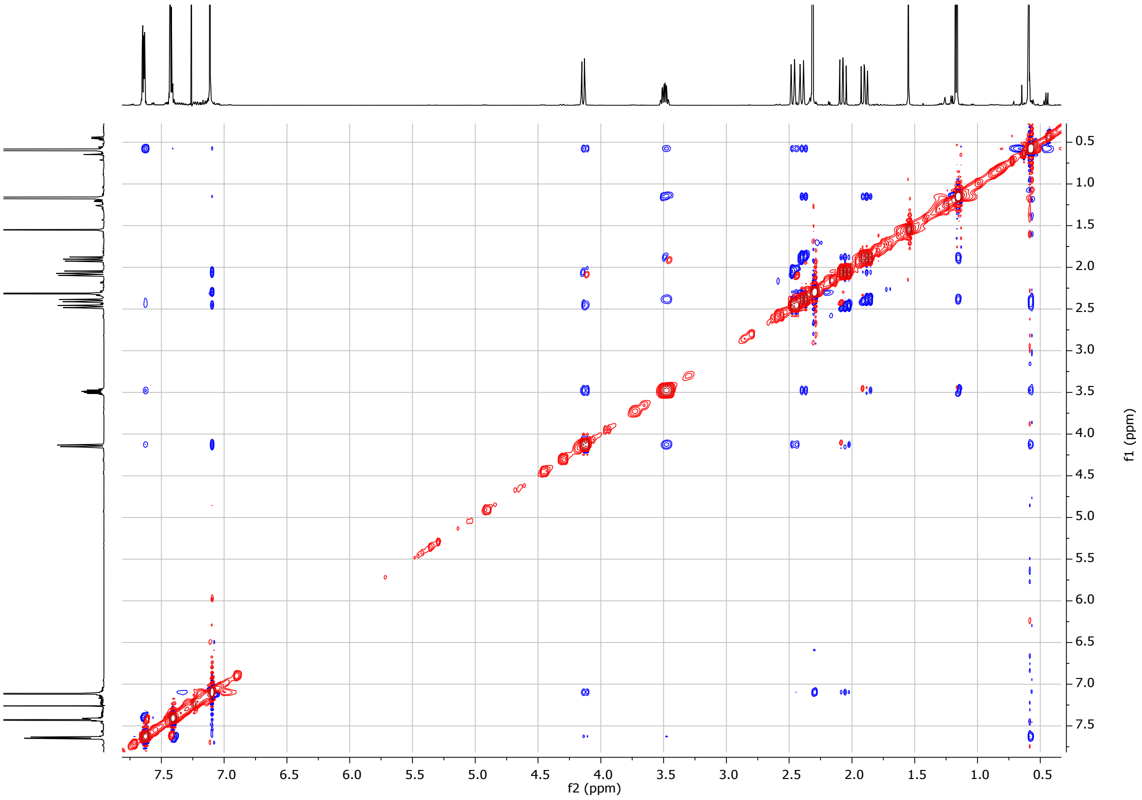

^{1}H NMR (400 MHz, CDCl$_3$)

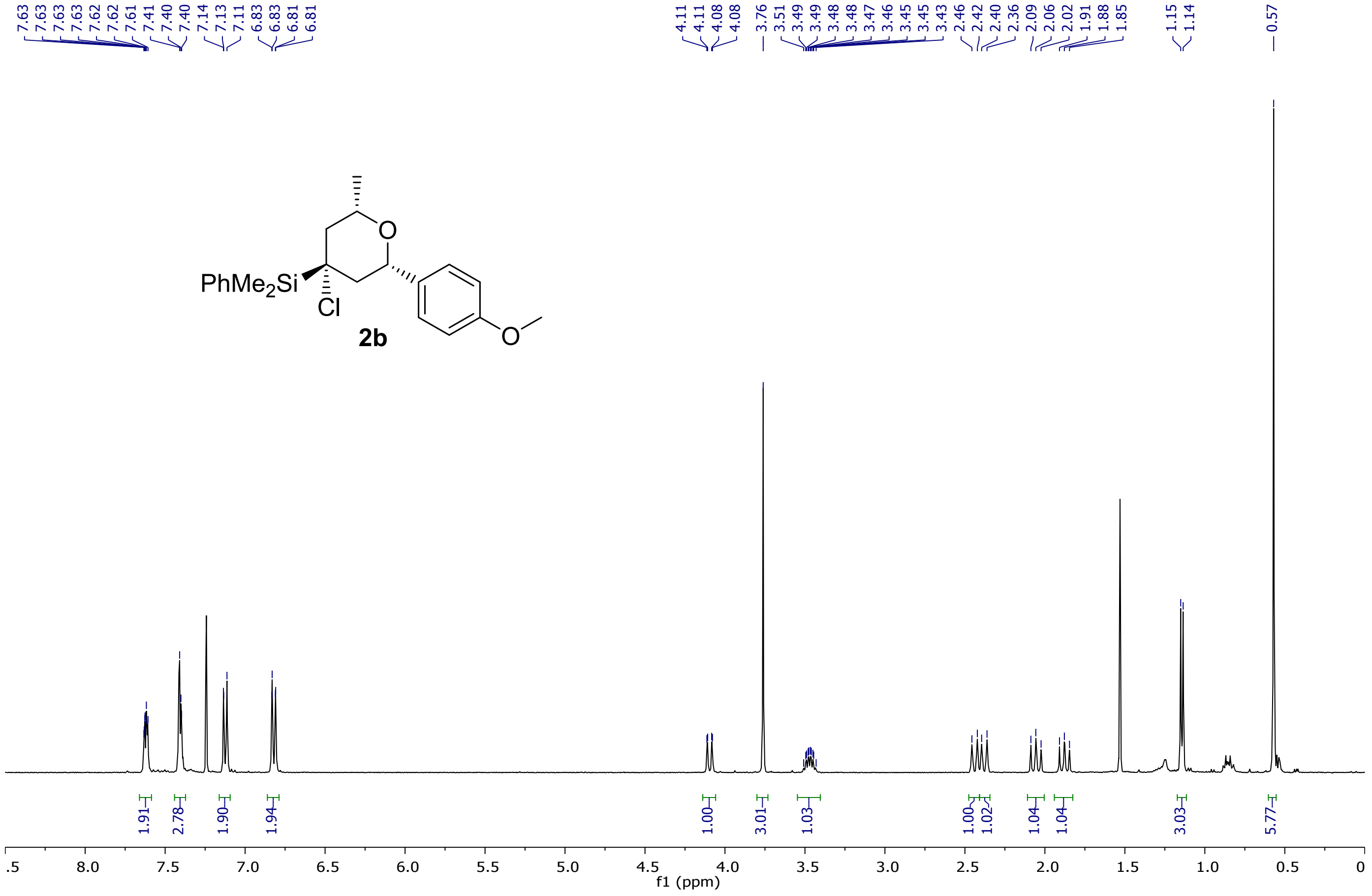

^{13}C {^{1}H} NMR (101 MHz, CDCl$_3$)

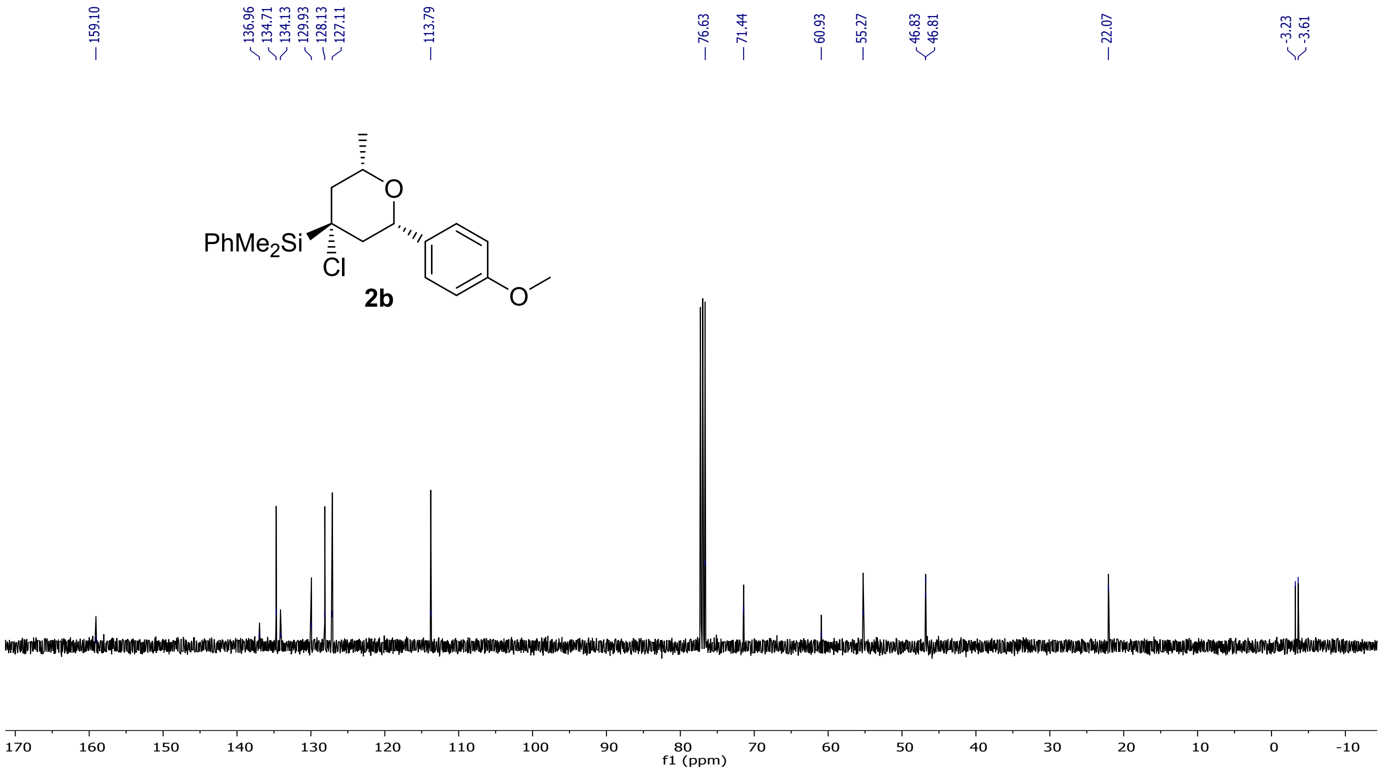

2D-COSY of compound 2b

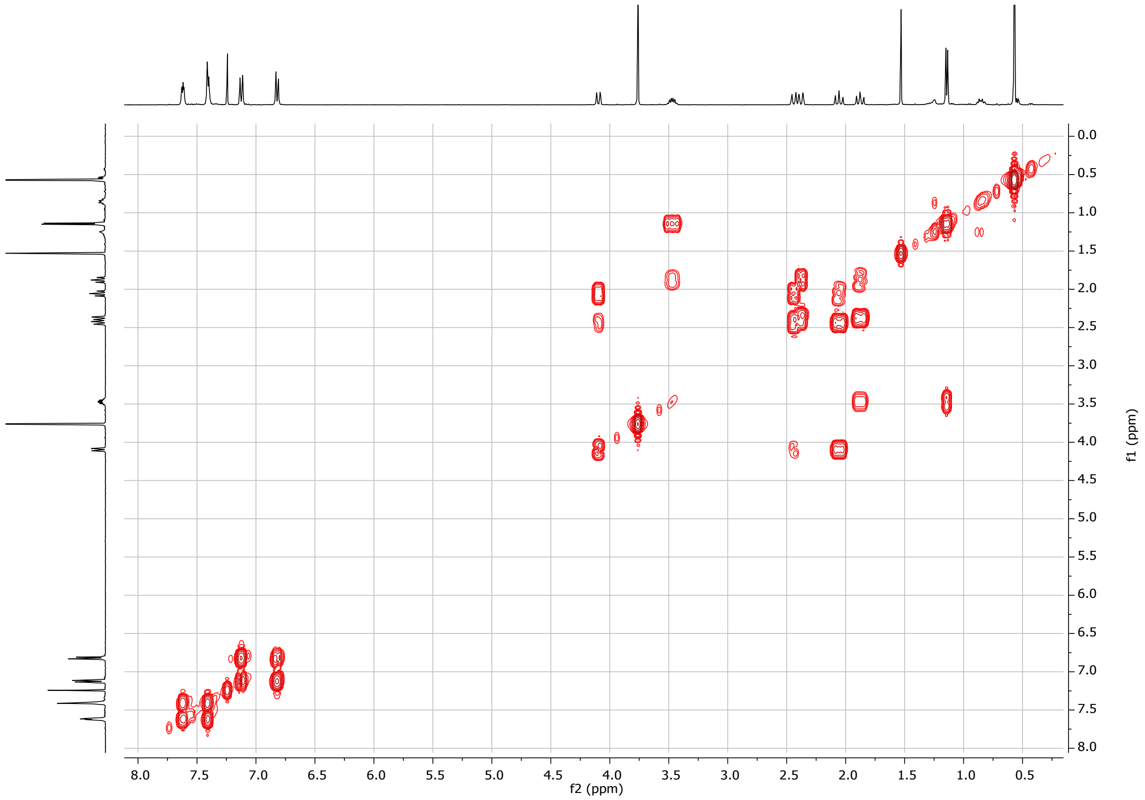

2D-HMBC of compound 2b

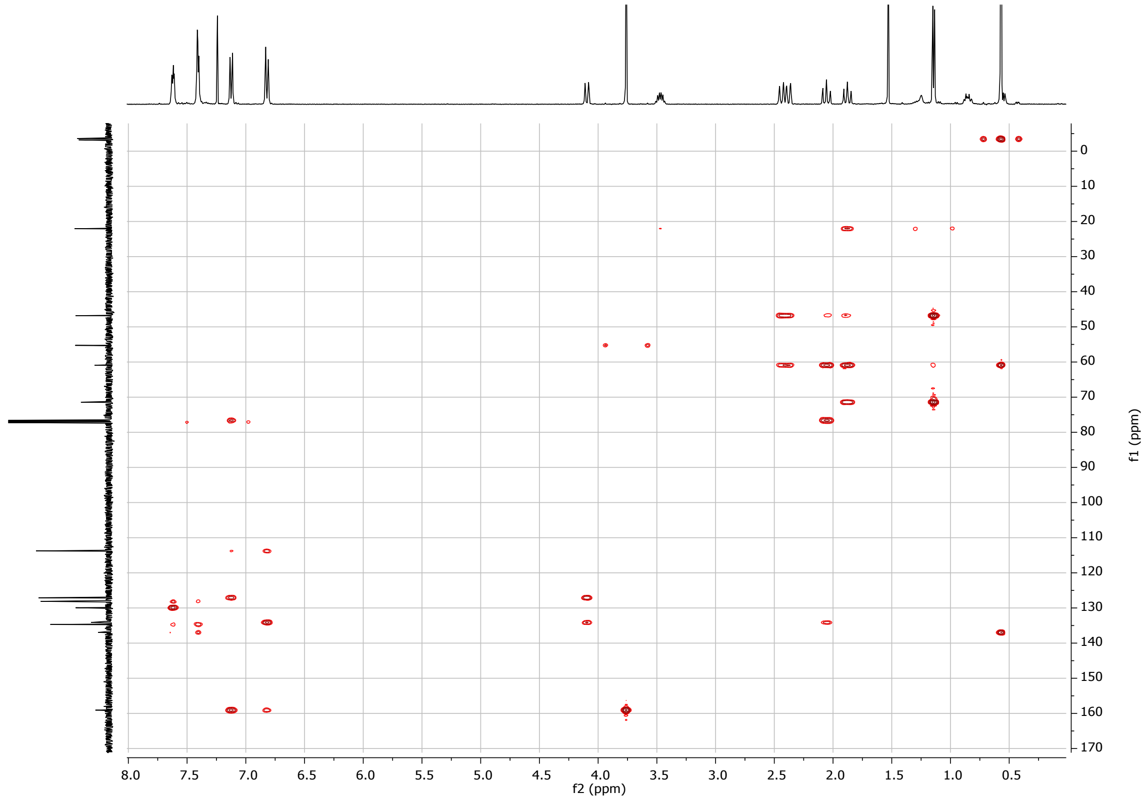

2D-NOESY of compound 2b

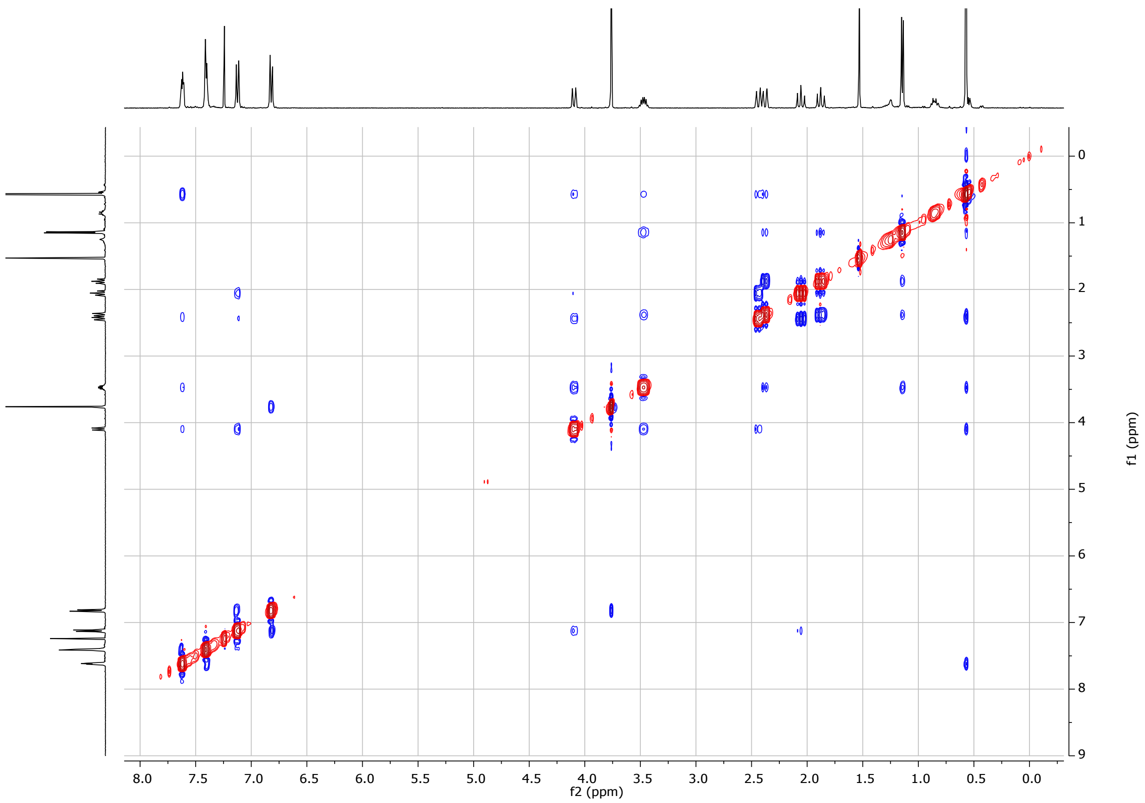

^{1}H NMR (400 MHz, $CDCl_3$)

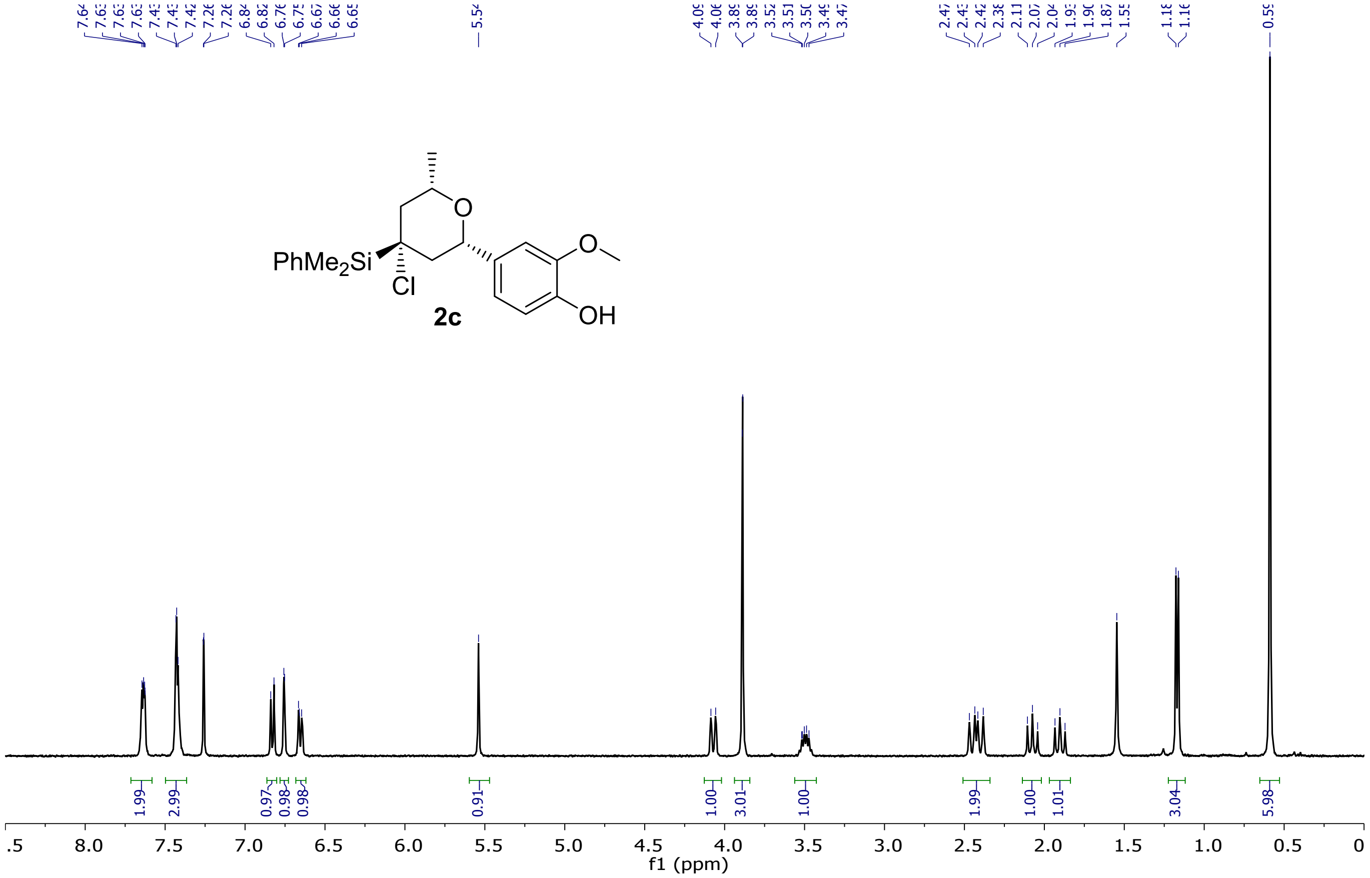

^{13}C {^{1}H} NMR (101 MHz, $CDCl_3$)

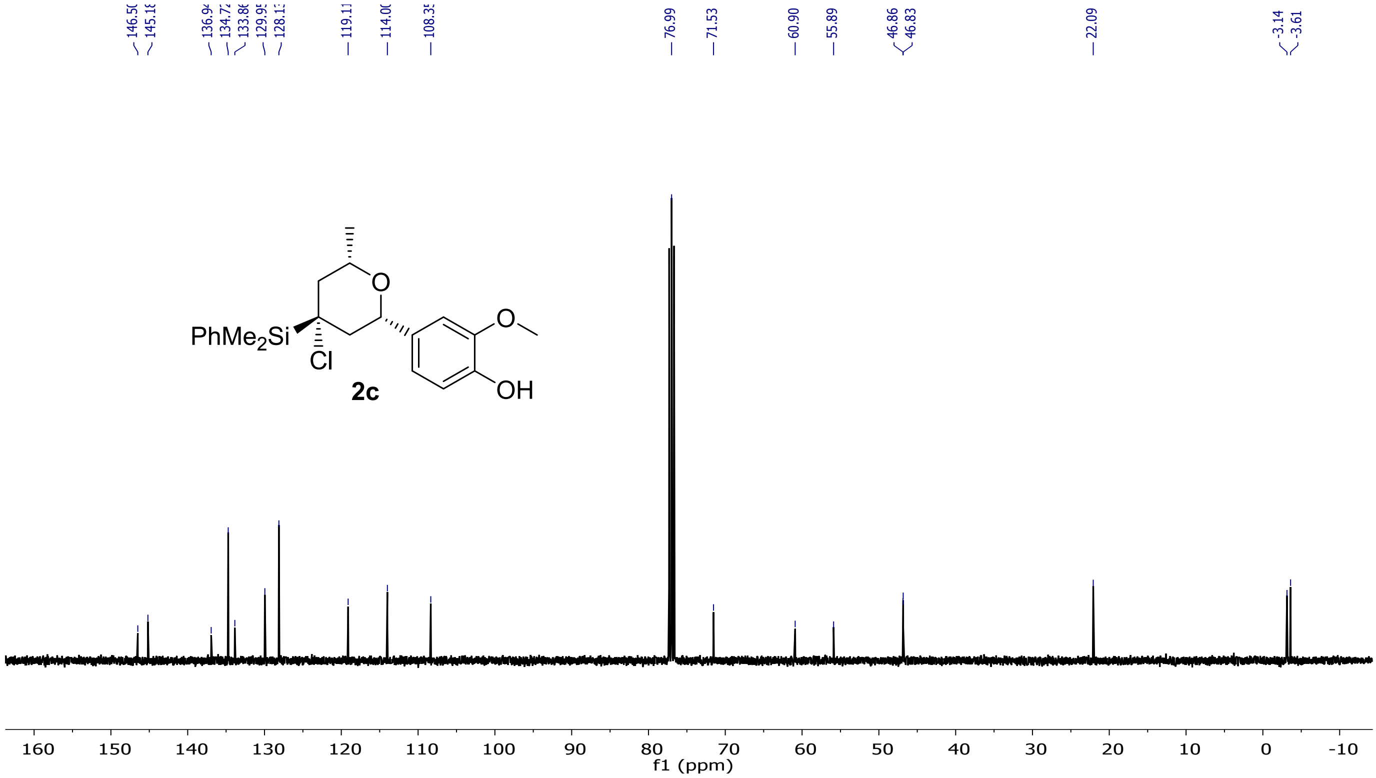

2D-COSY of compound 2c

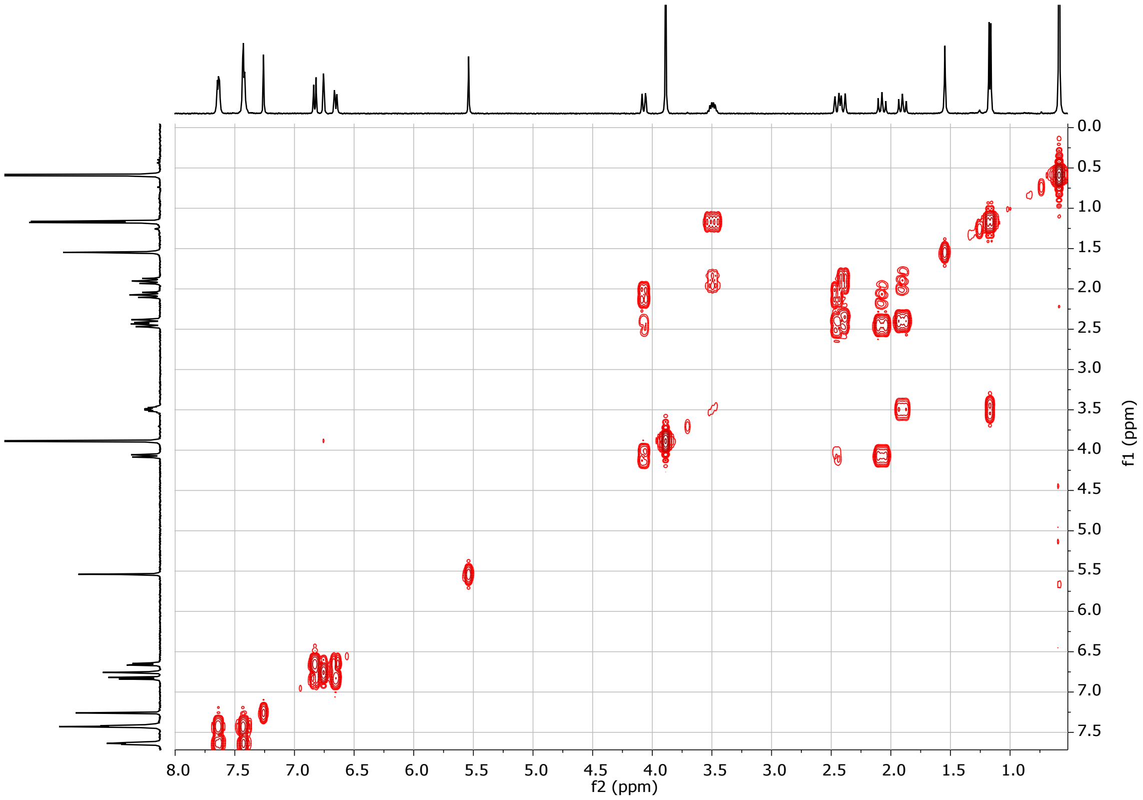

2D-HMBC of compound 2c

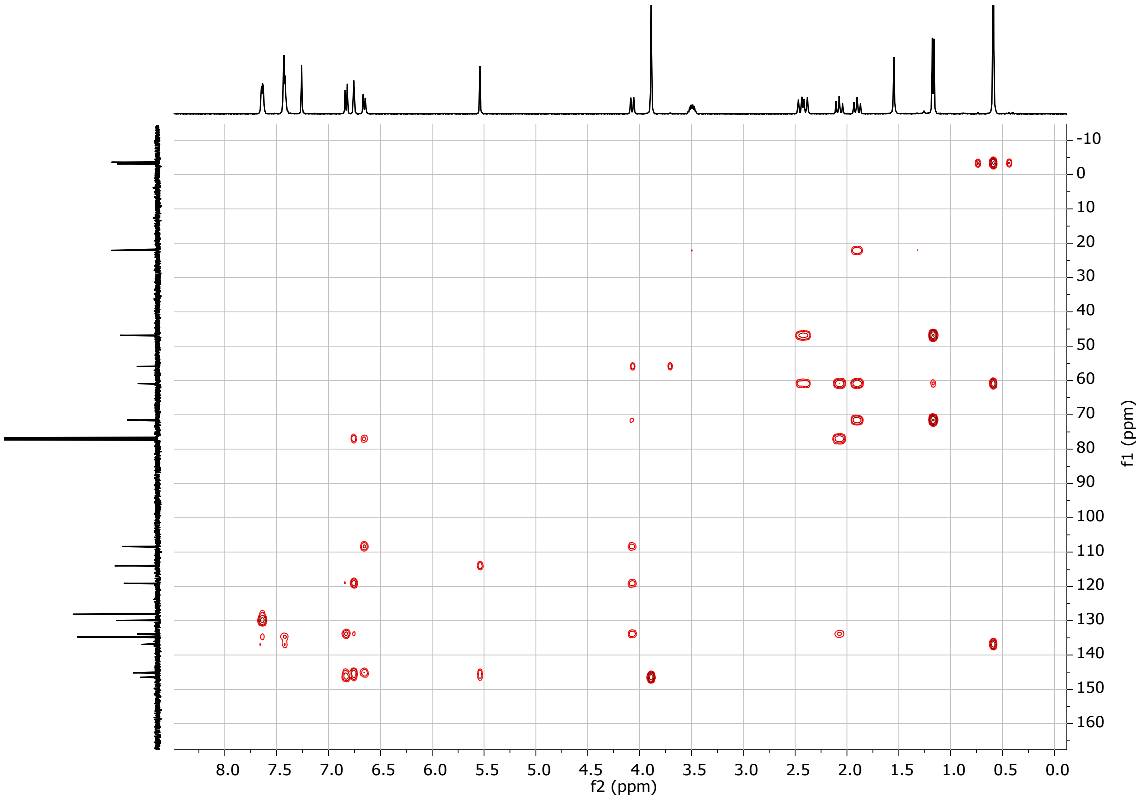

2D-NOESY of compound 2c

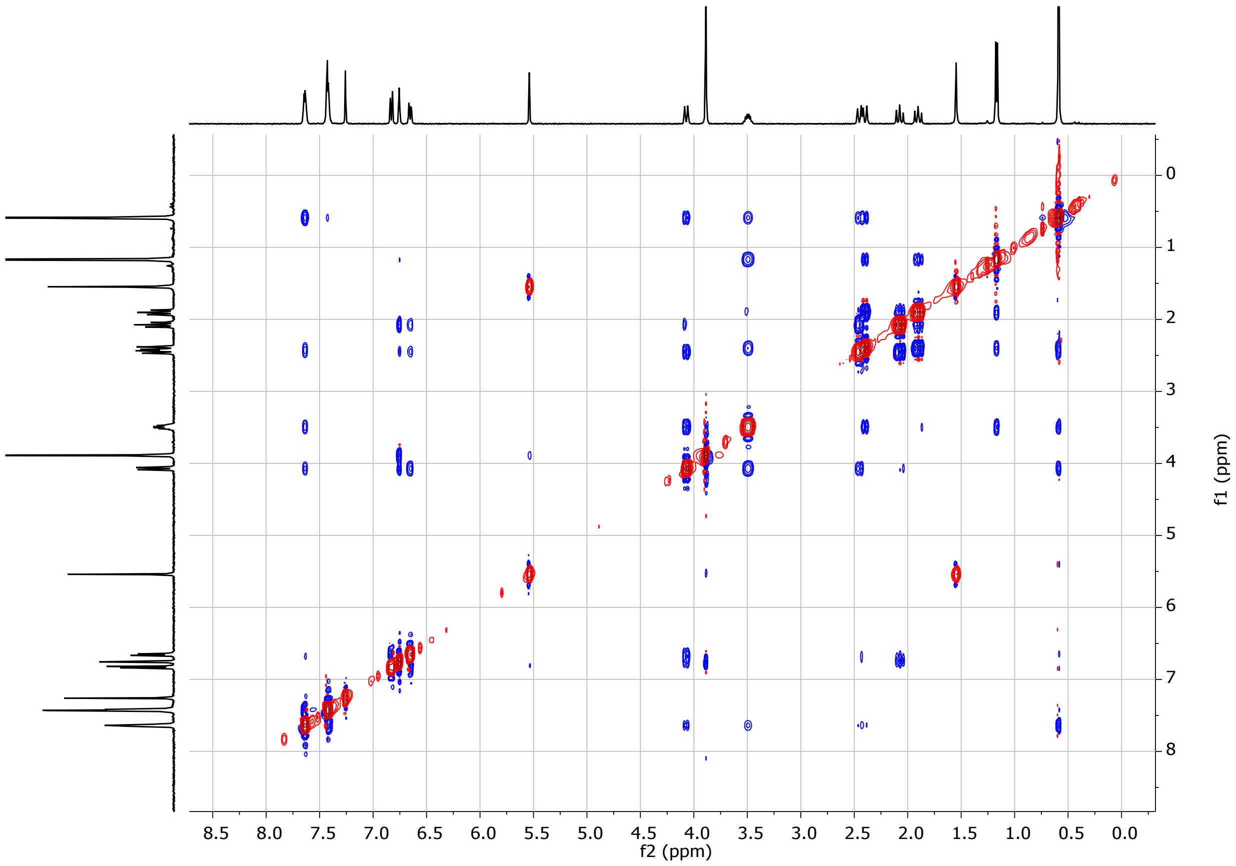

^{1}H NMR (400 MHz, $CDCl_3$)

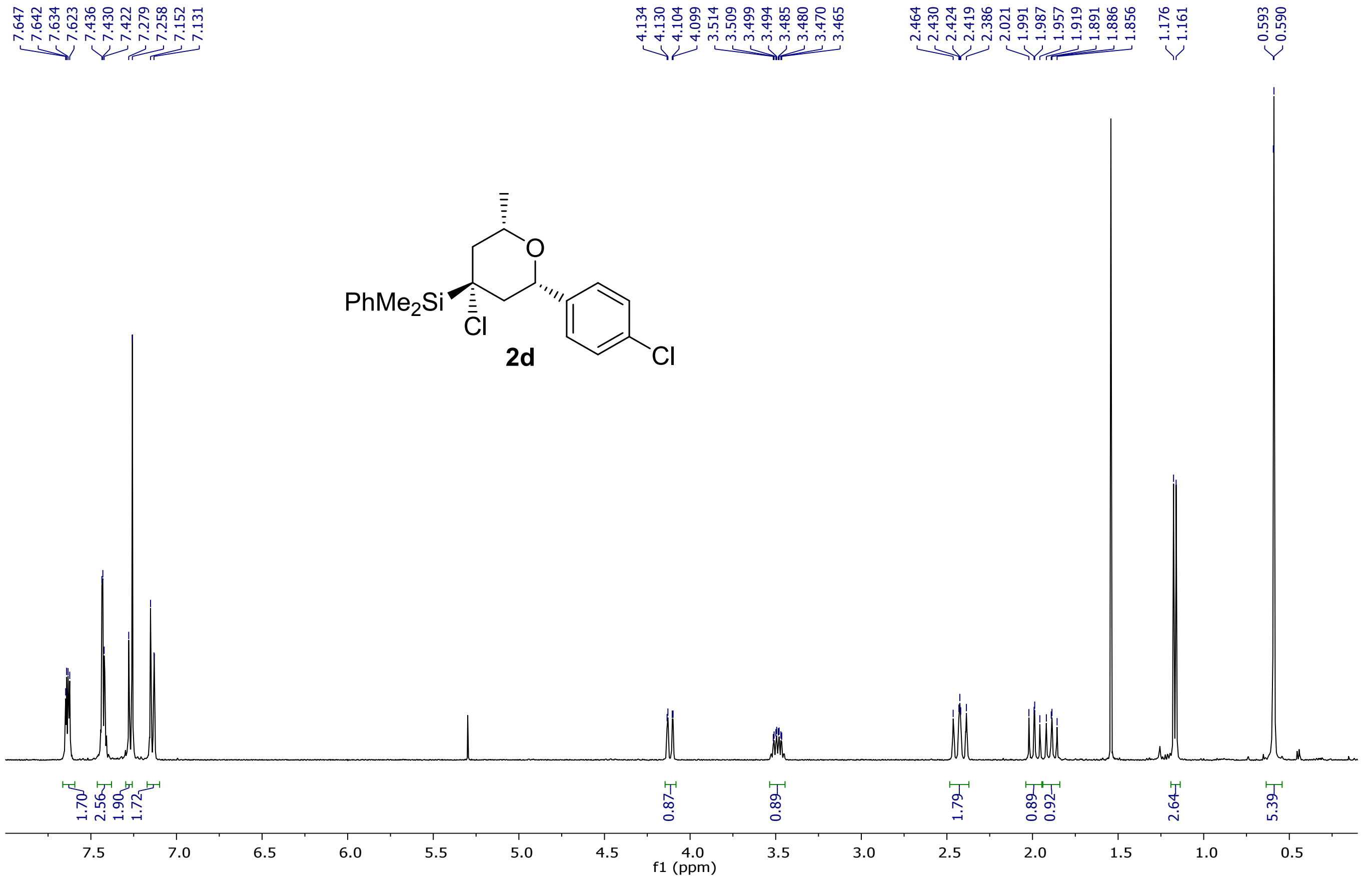

^{13}C {^{1}H} NMR (101 MHz, $CDCl_3$)

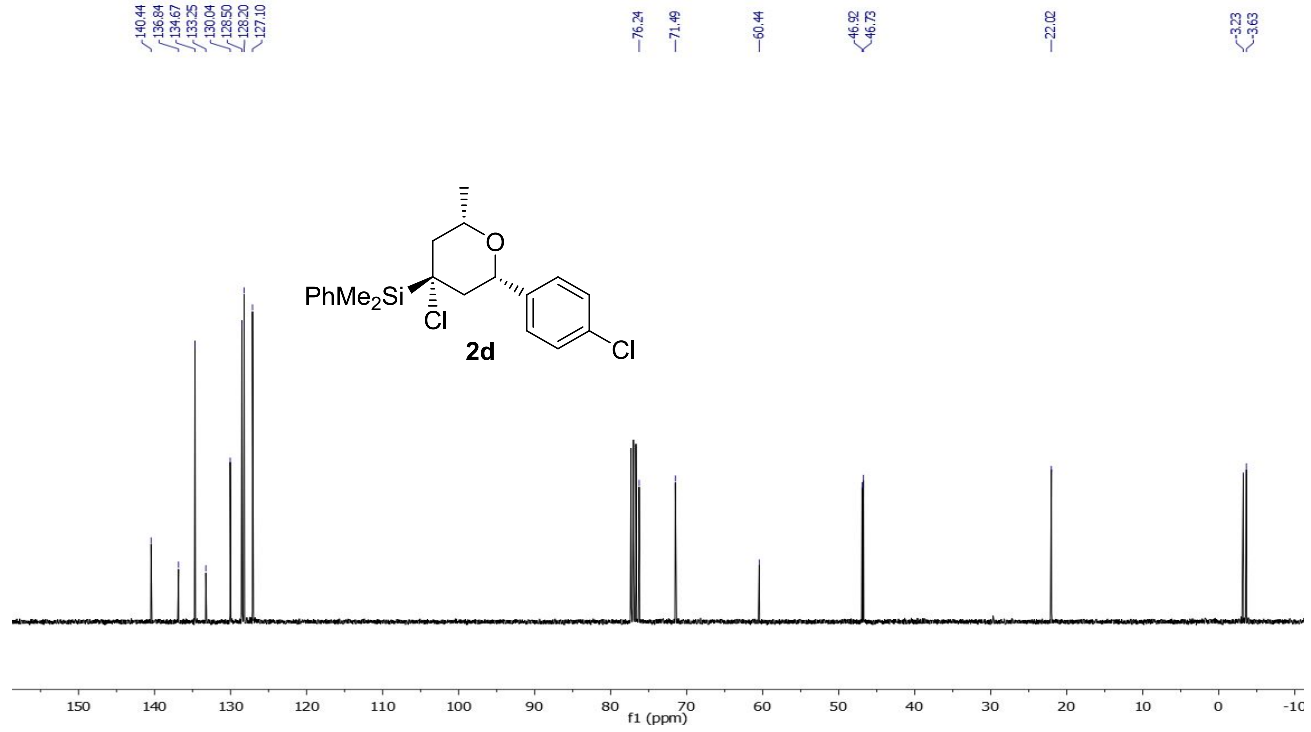

2D-COSY of compound 2d

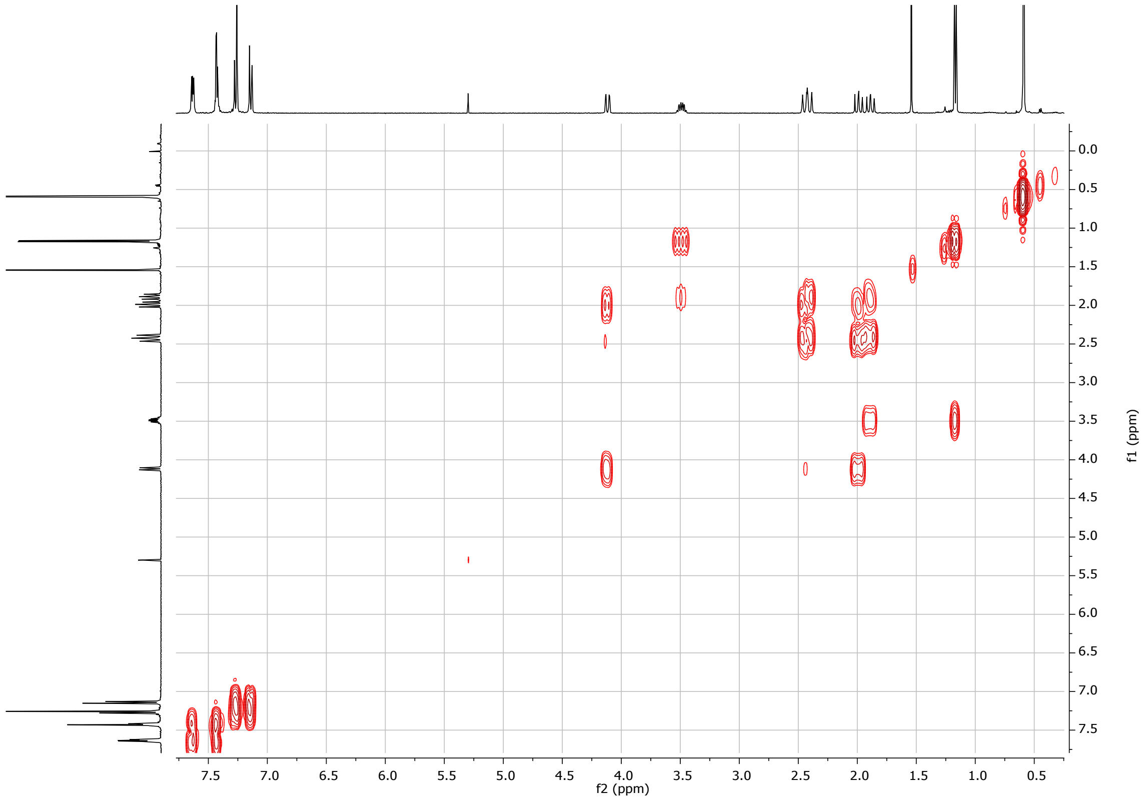

2D-HMBC of compound 2d

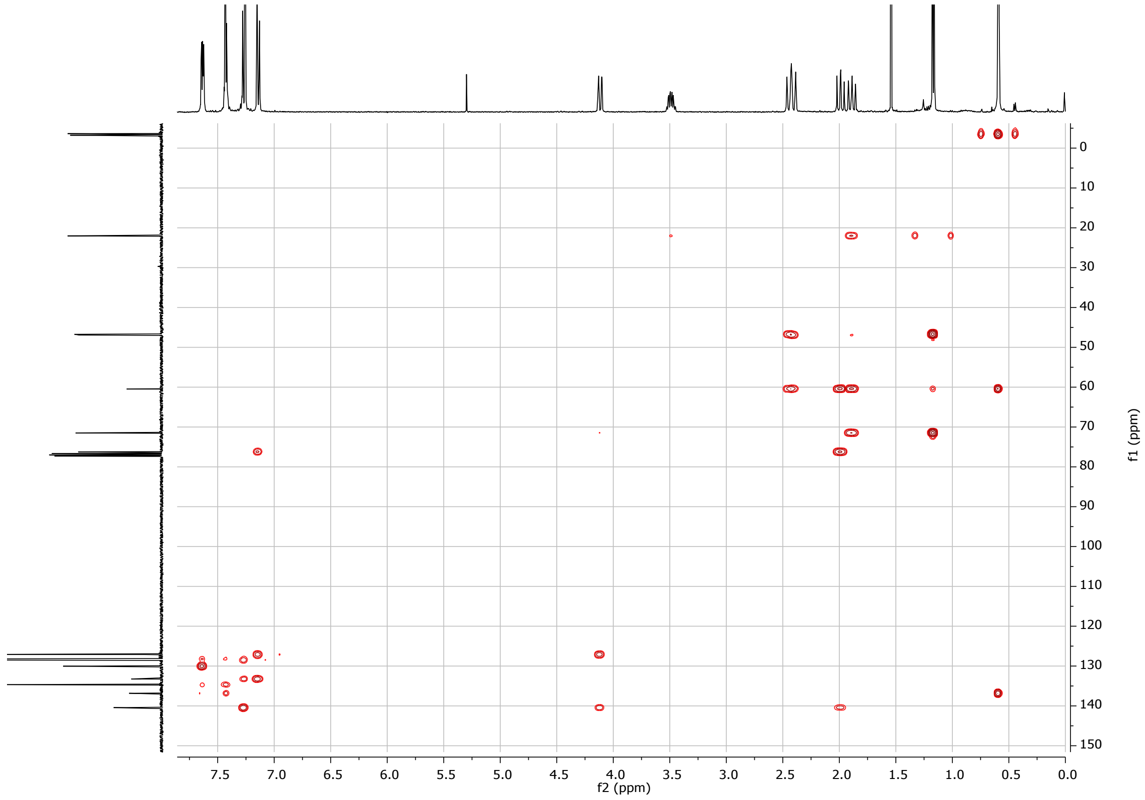

2D-NOESY of compound 2d

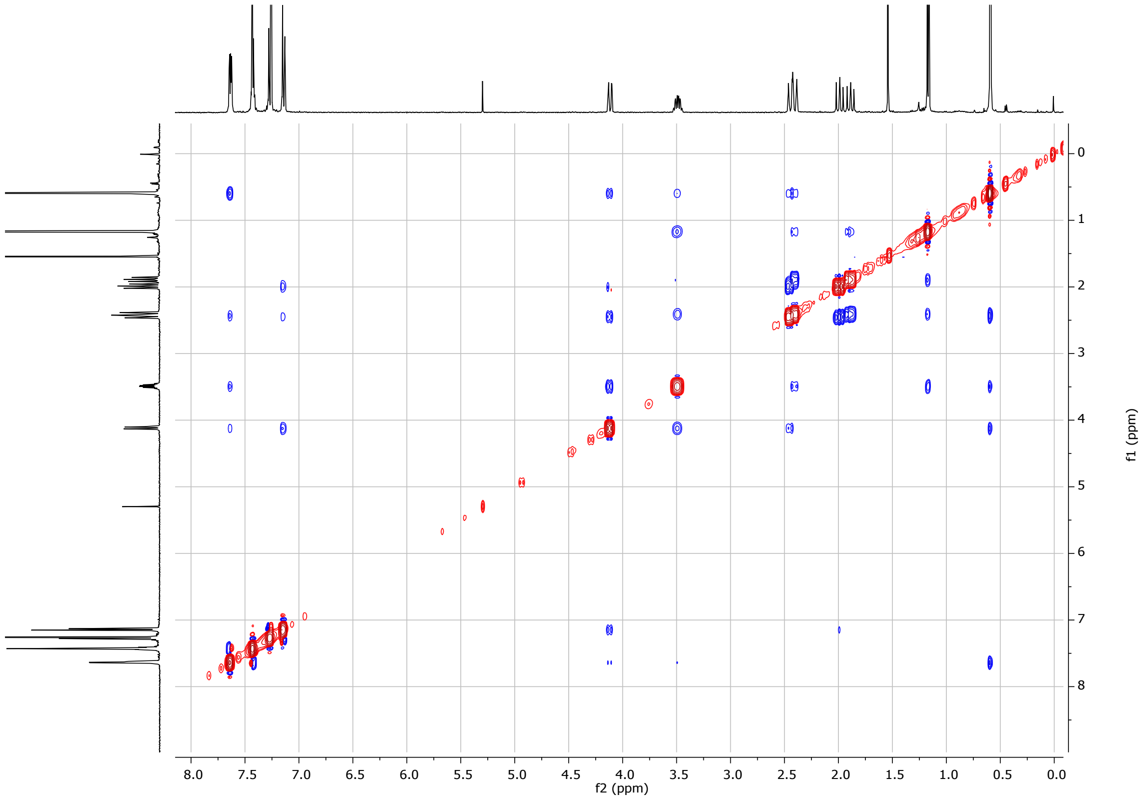

^{1}H NMR (400 MHz, $CDCl_3$)

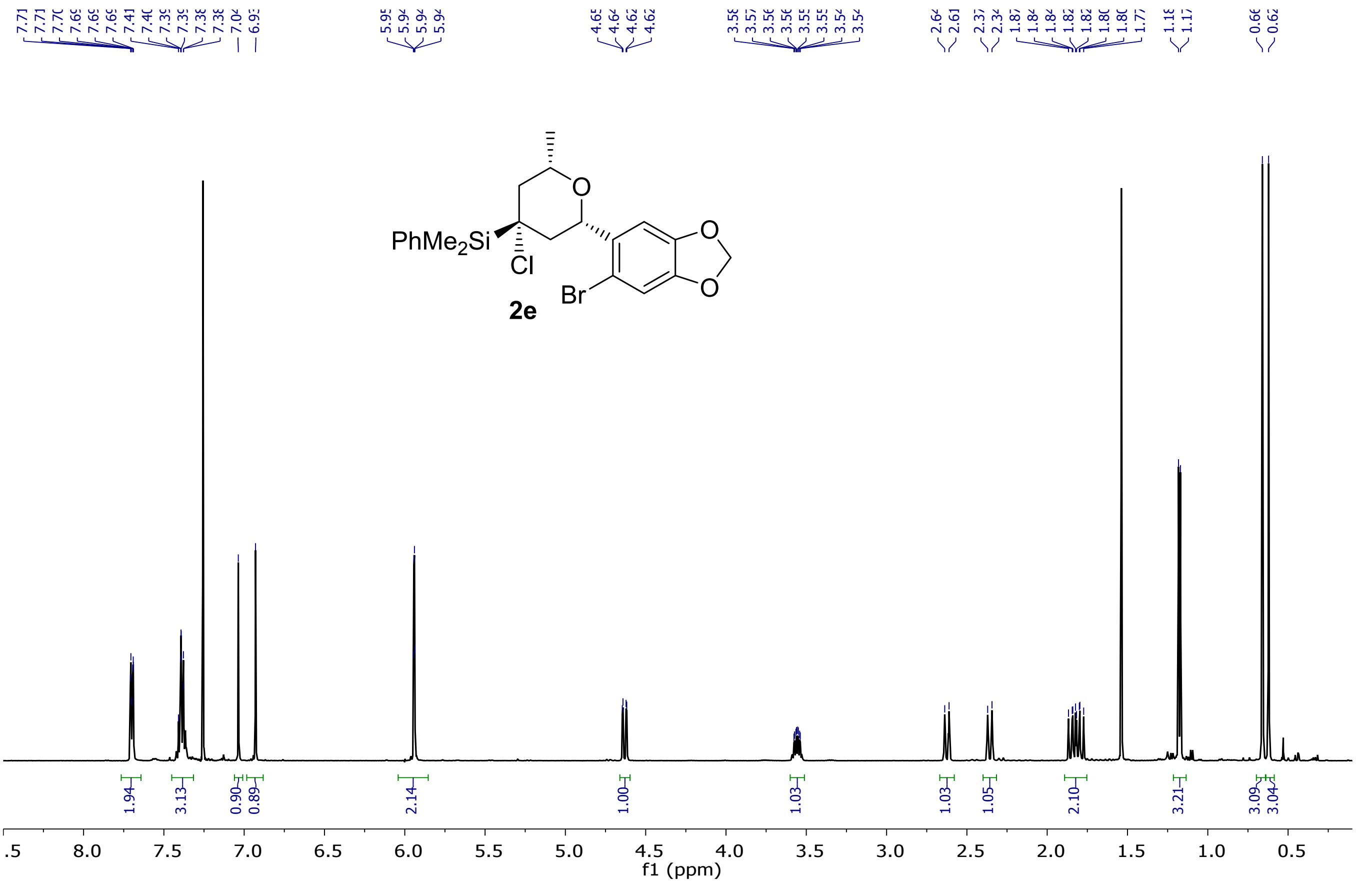

^{13}C {^{1}H} NMR (101 MHz, $CDCl_3$)

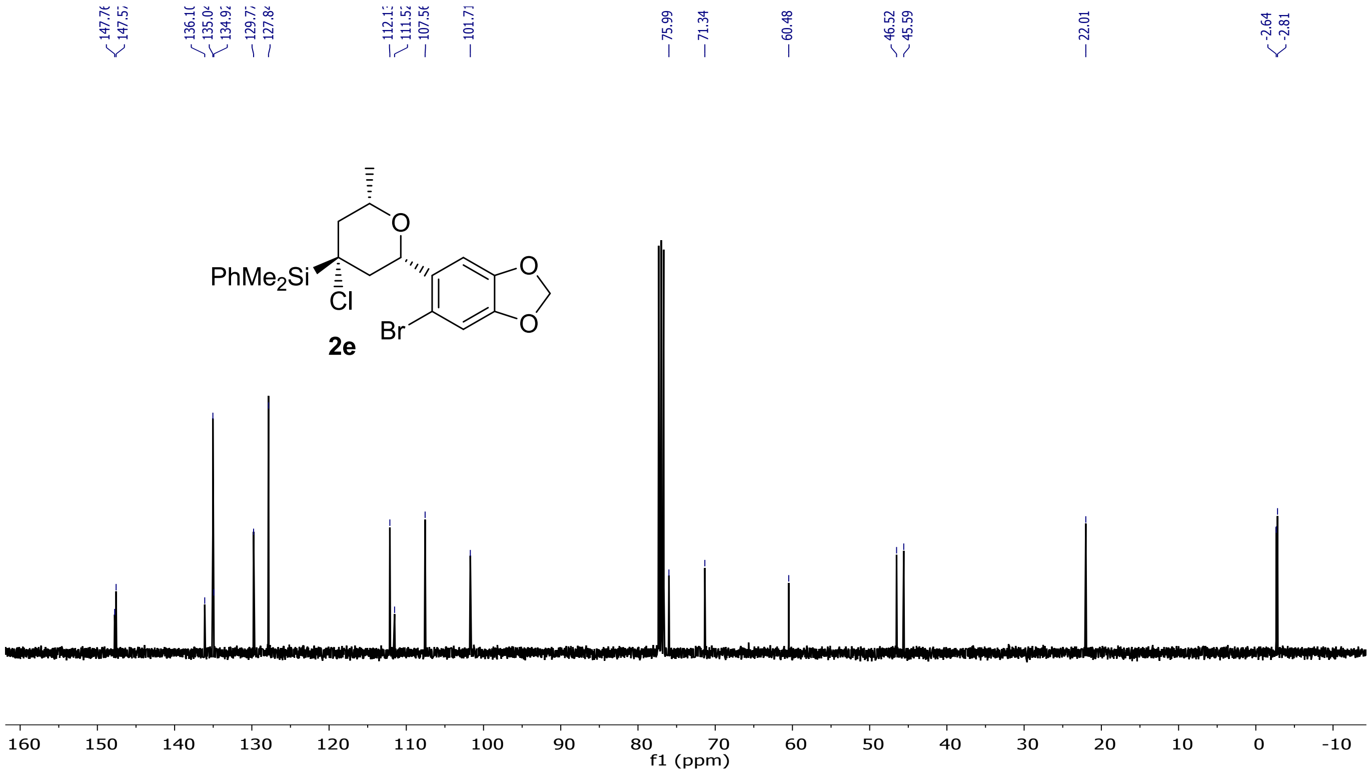

2D-COSY of compound 2e

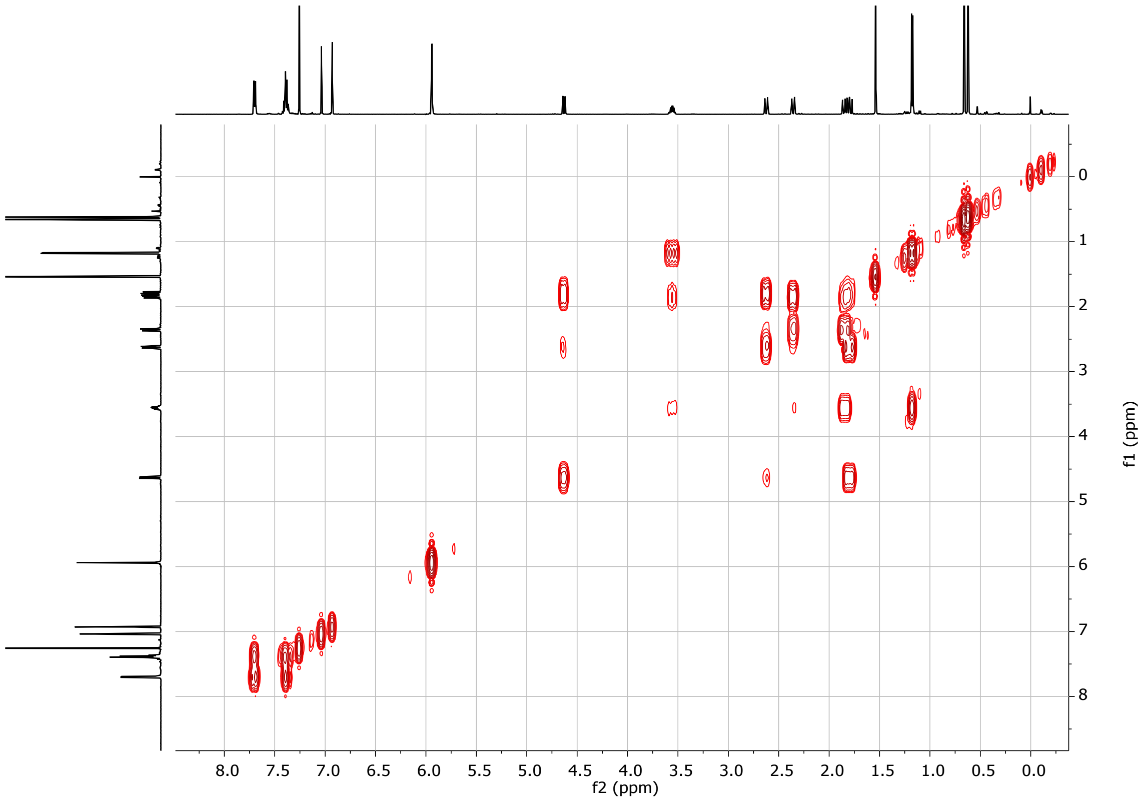

2D-HMBC of compound 2e

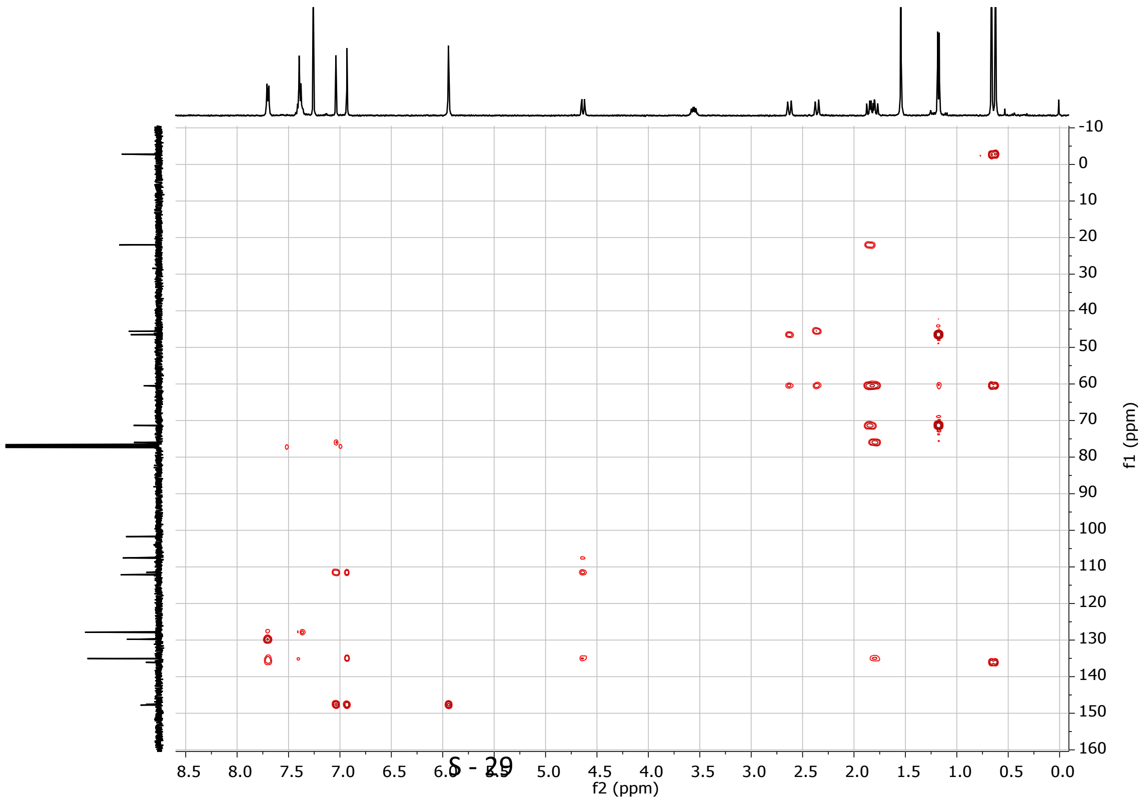

2D-NOESY of compound 2e

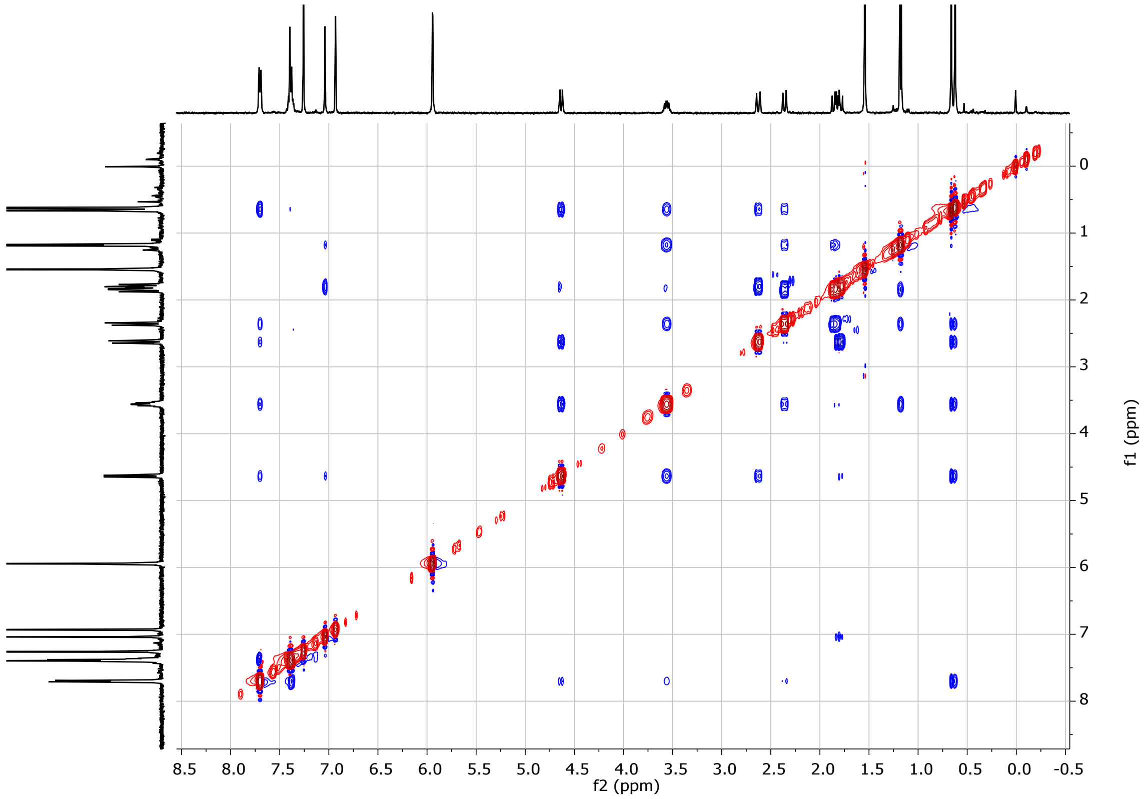

^{1}H NMR (400 MHz, CDCl$_3$)

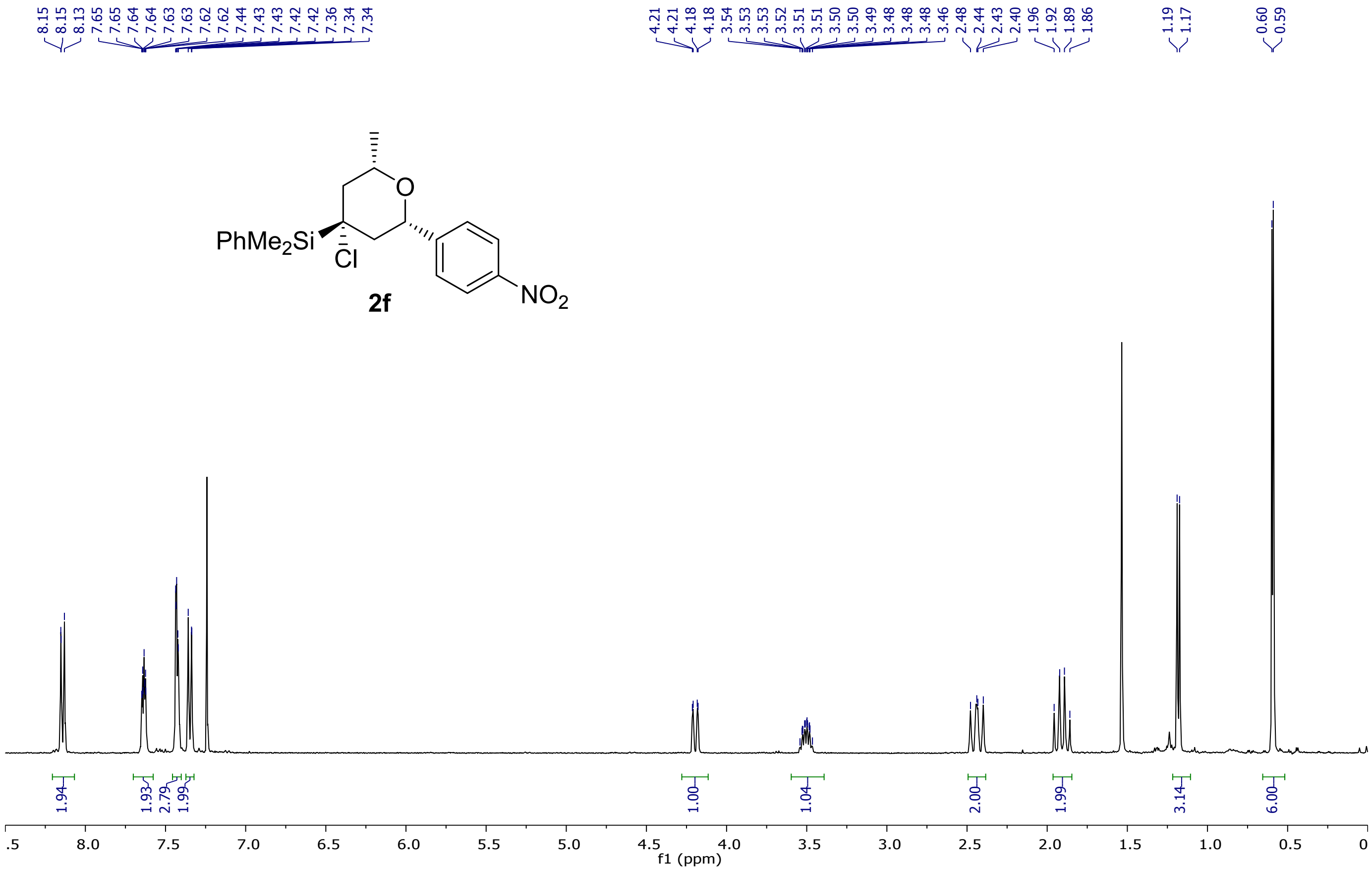

^{13}C {^{1}H} NMR (101 MHz, CDCl$_3$)

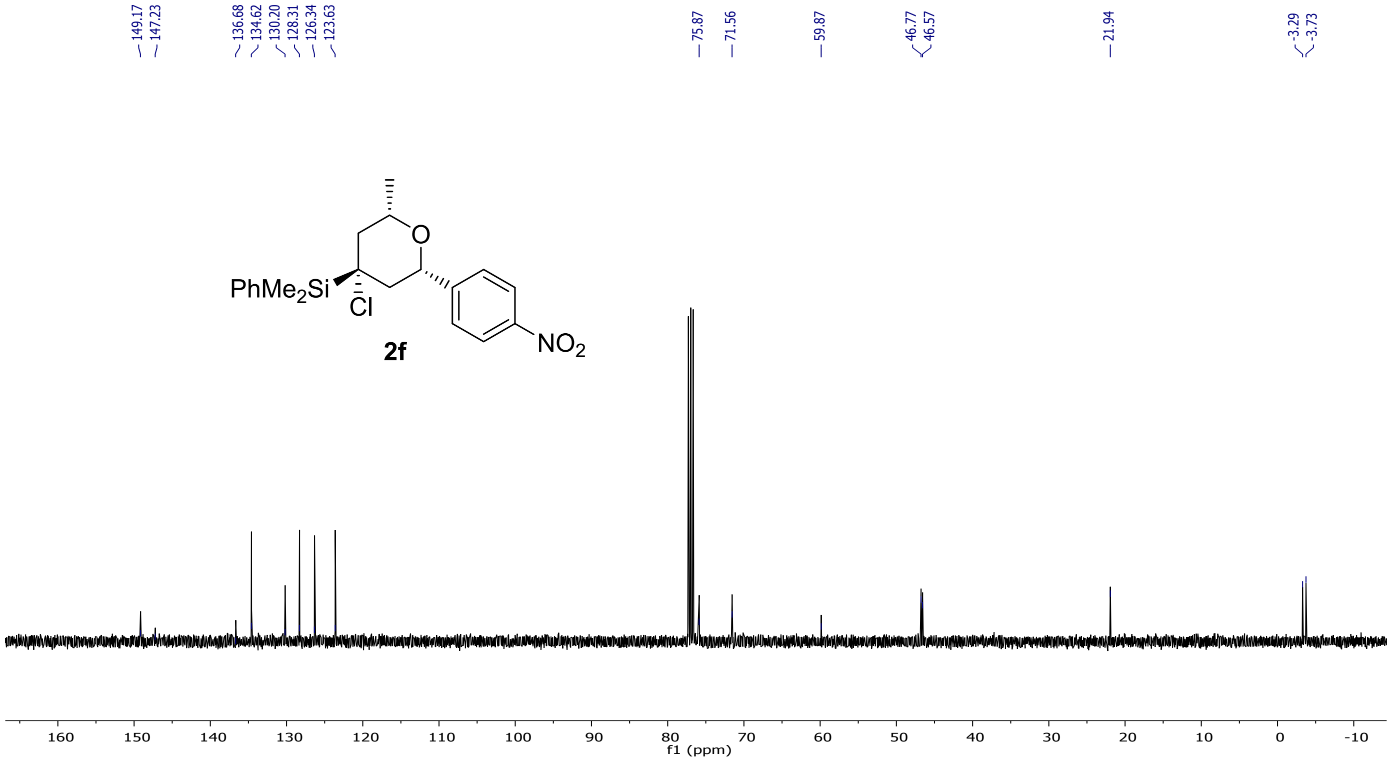

2D-COSY of compound 2f

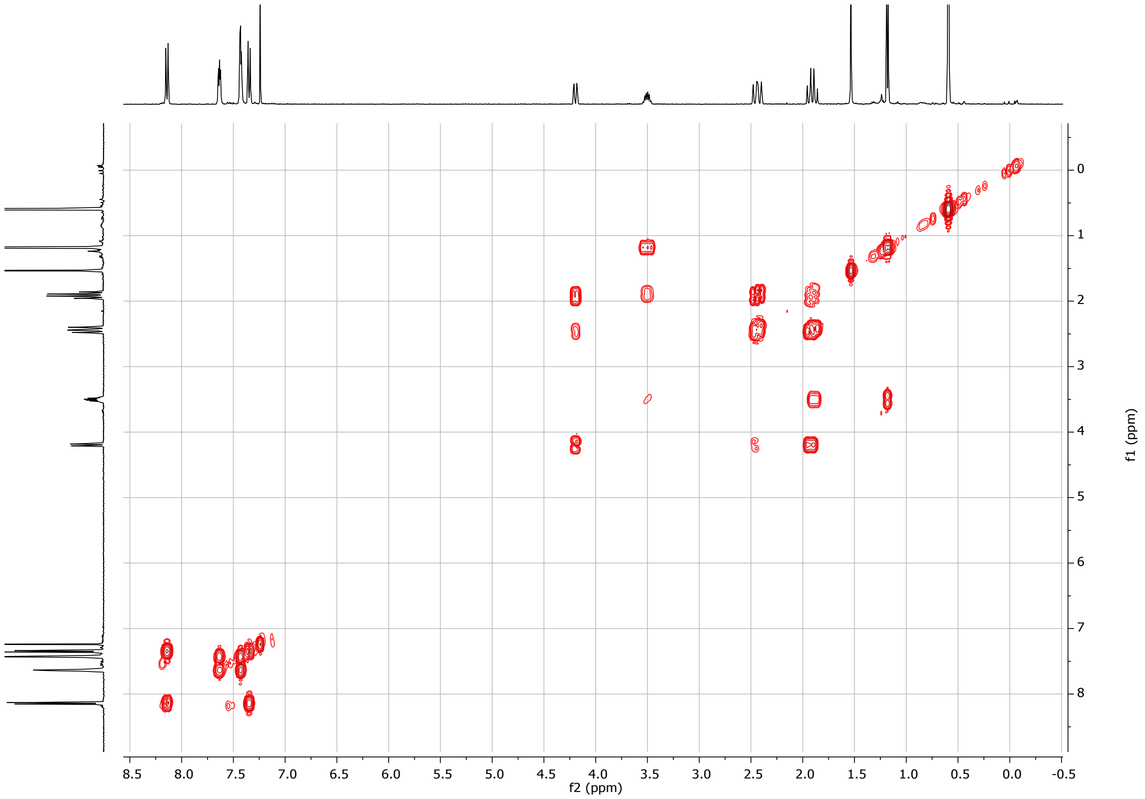

2D-HMBC of compound 2f

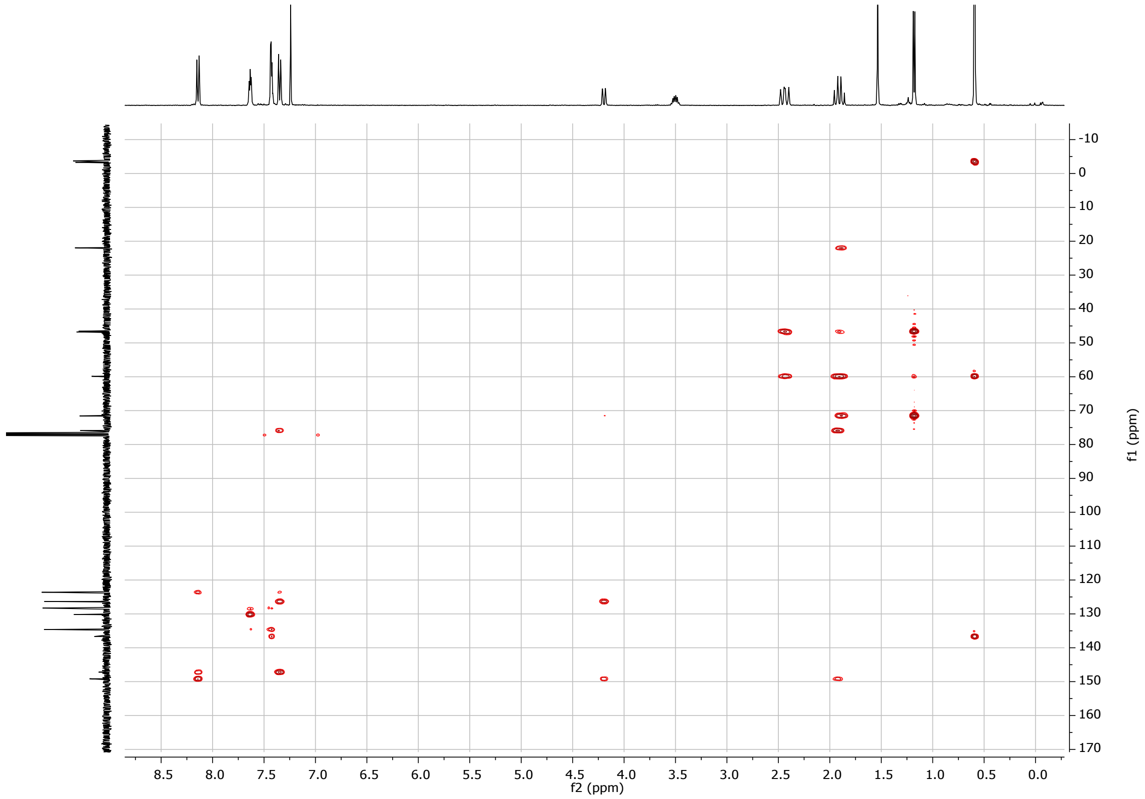

2D-NOESY of compound 2f

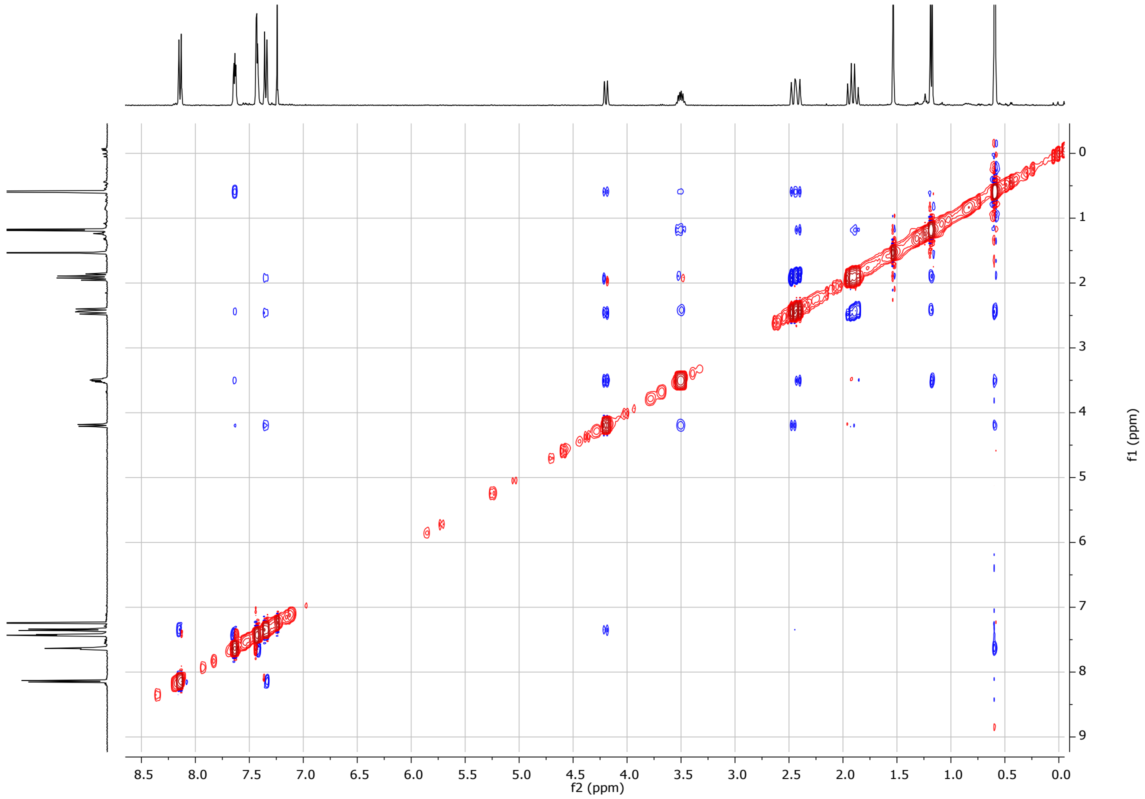

^{1}H NMR (400 MHz, CDCl$_3$)

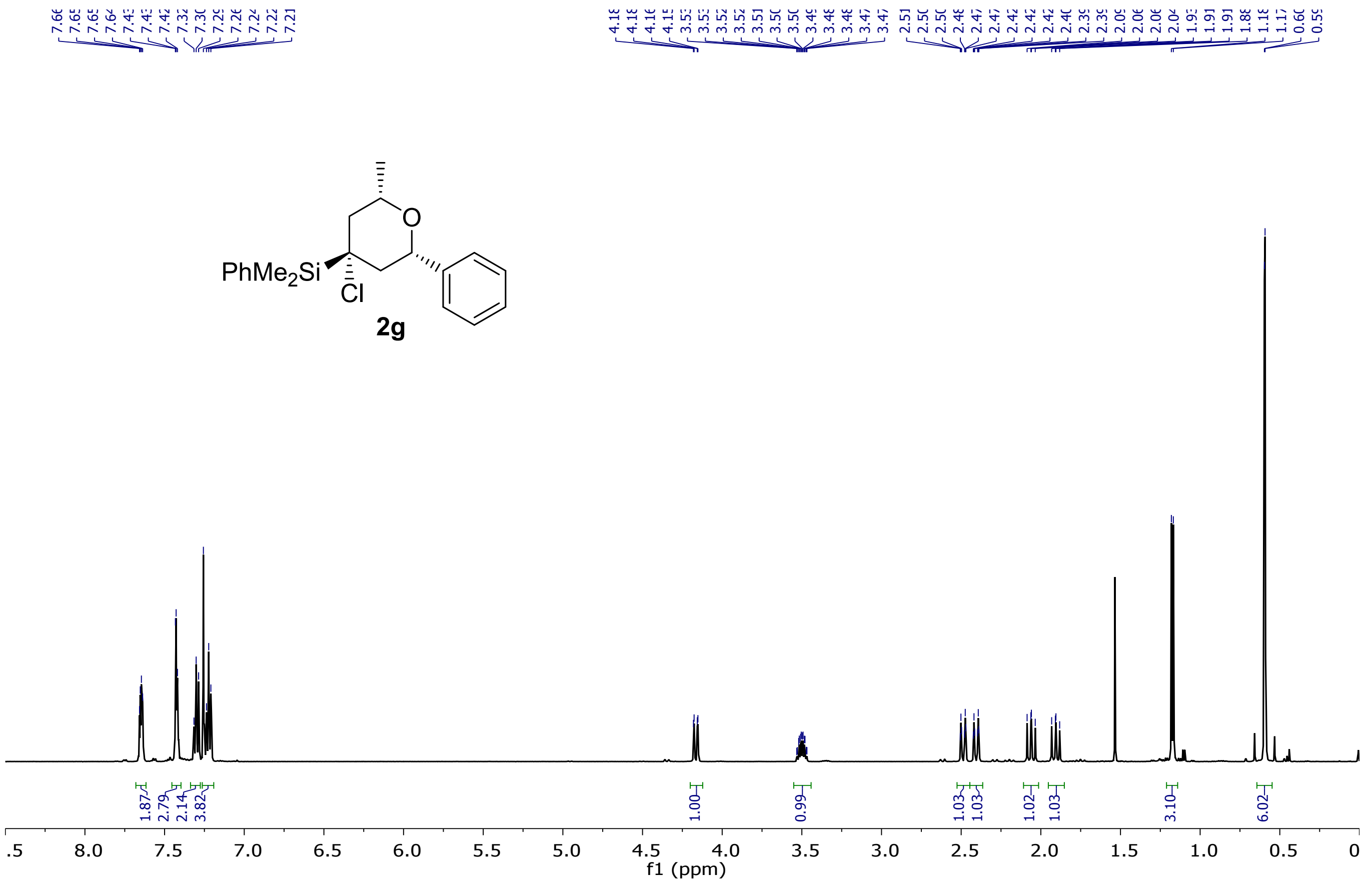

^{13}C {^{1}H} NMR (101 MHz, CDCl$_3$)

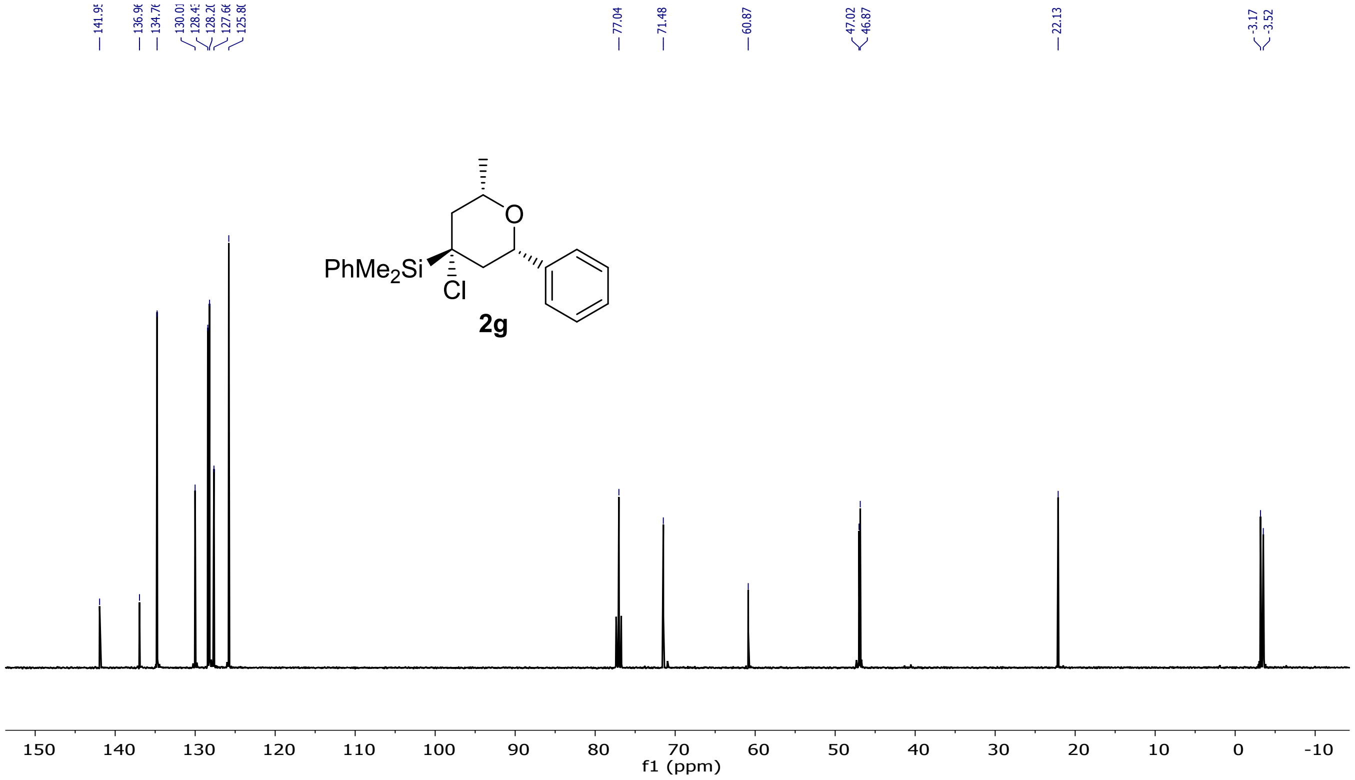

2D-COSY of compound 2g

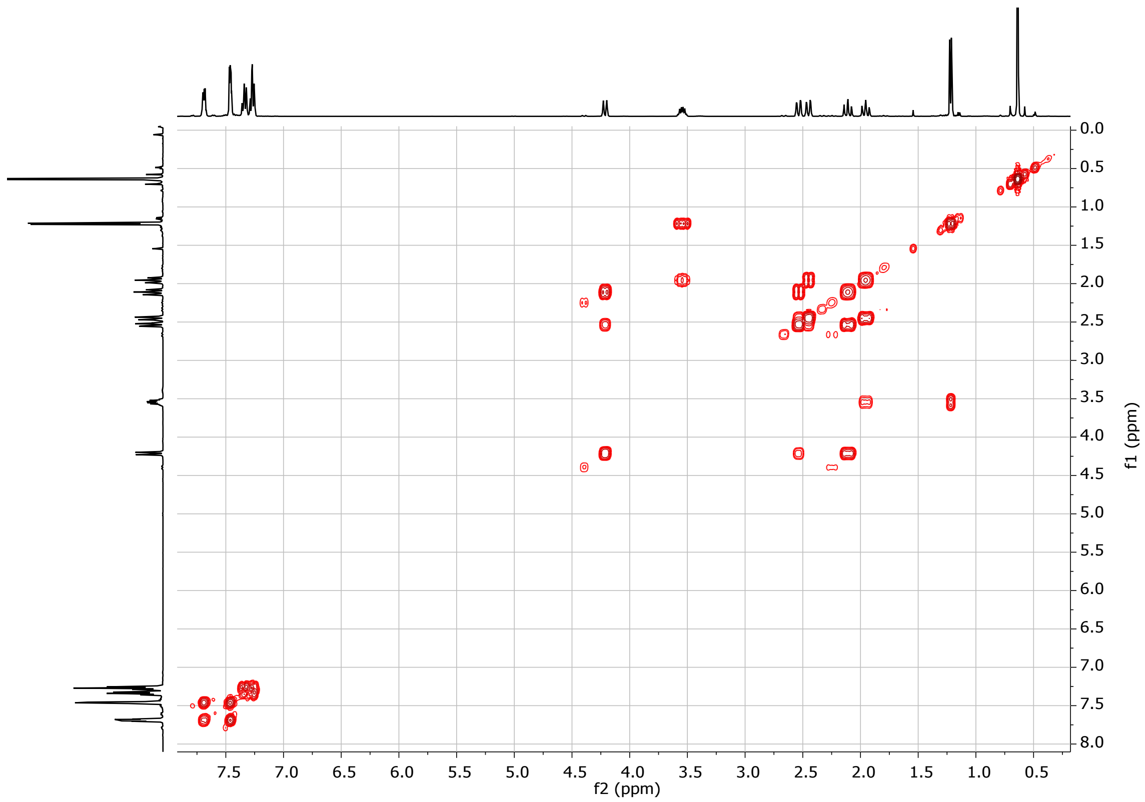

2D-HMBC of compound 2g

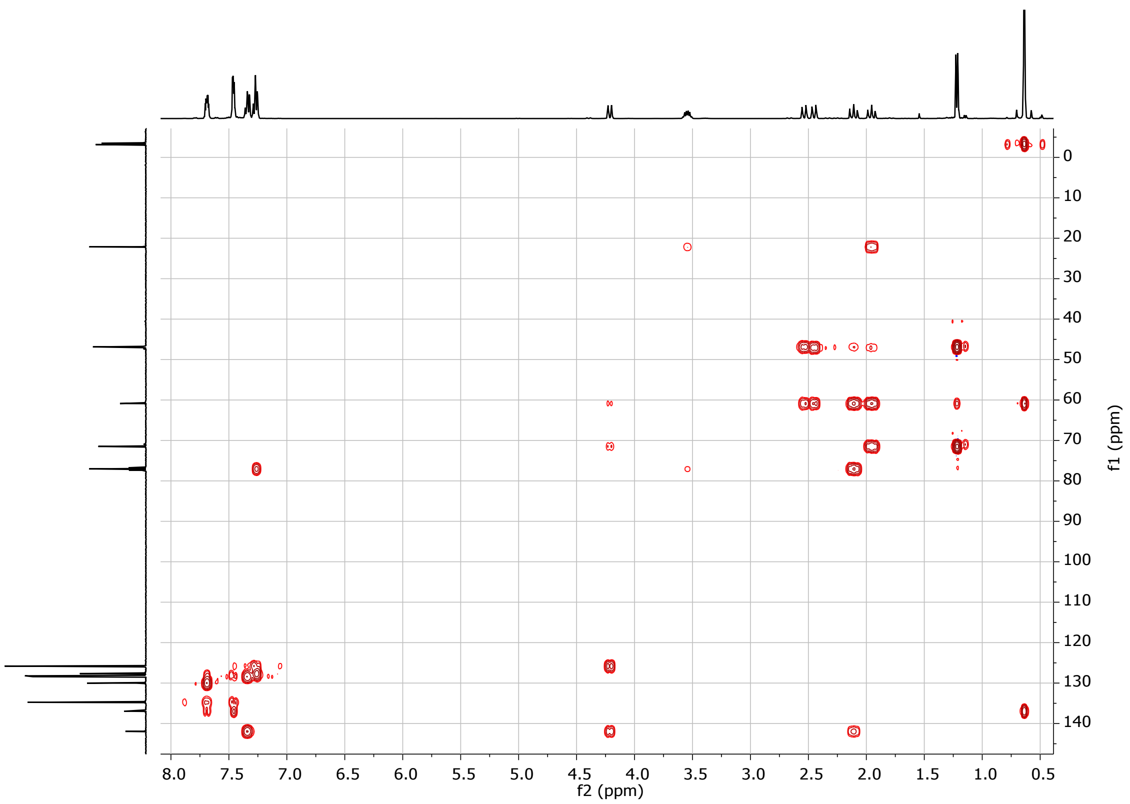

2D-NOESY of compound 2g

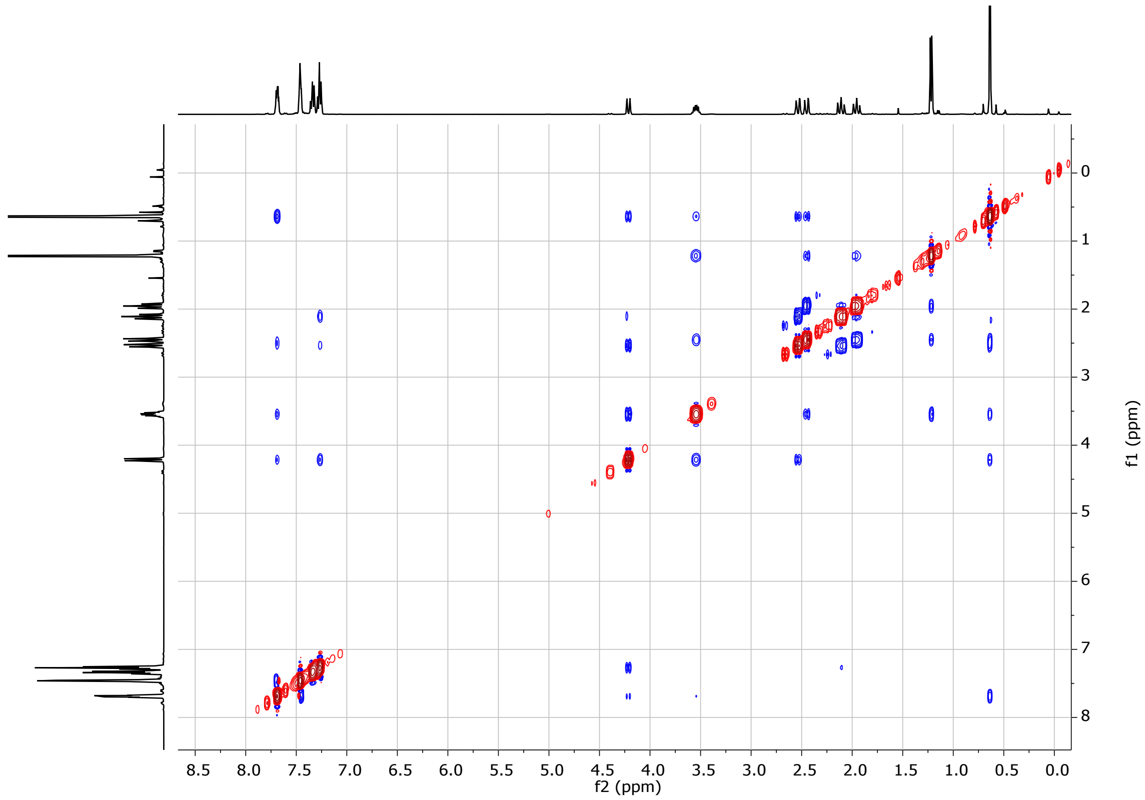

^{1}H NMR (400 MHz, CDCl$_3$)

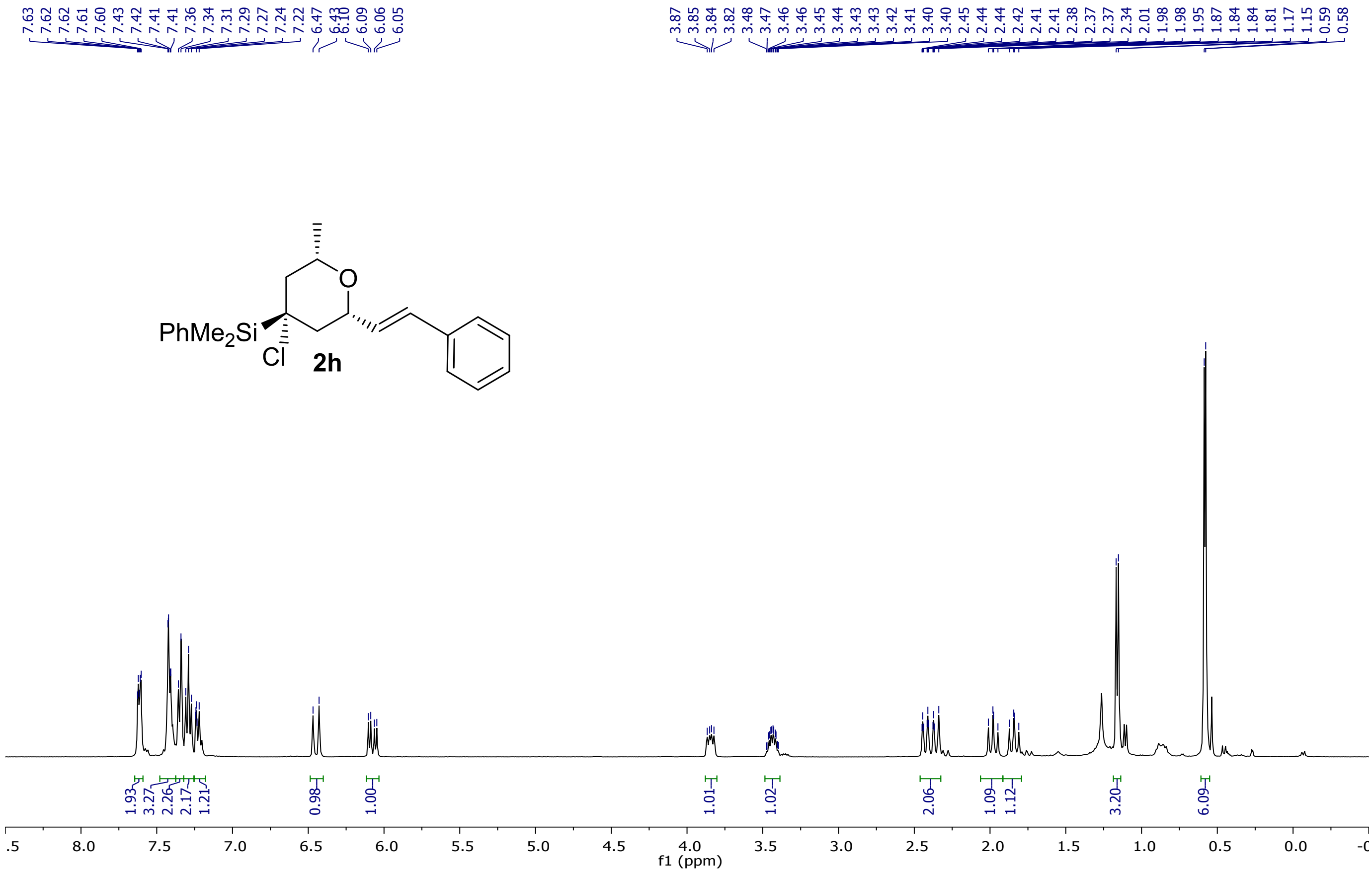

^{13}C {^{1}H} NMR (101 MHz, CDCl$_3$)

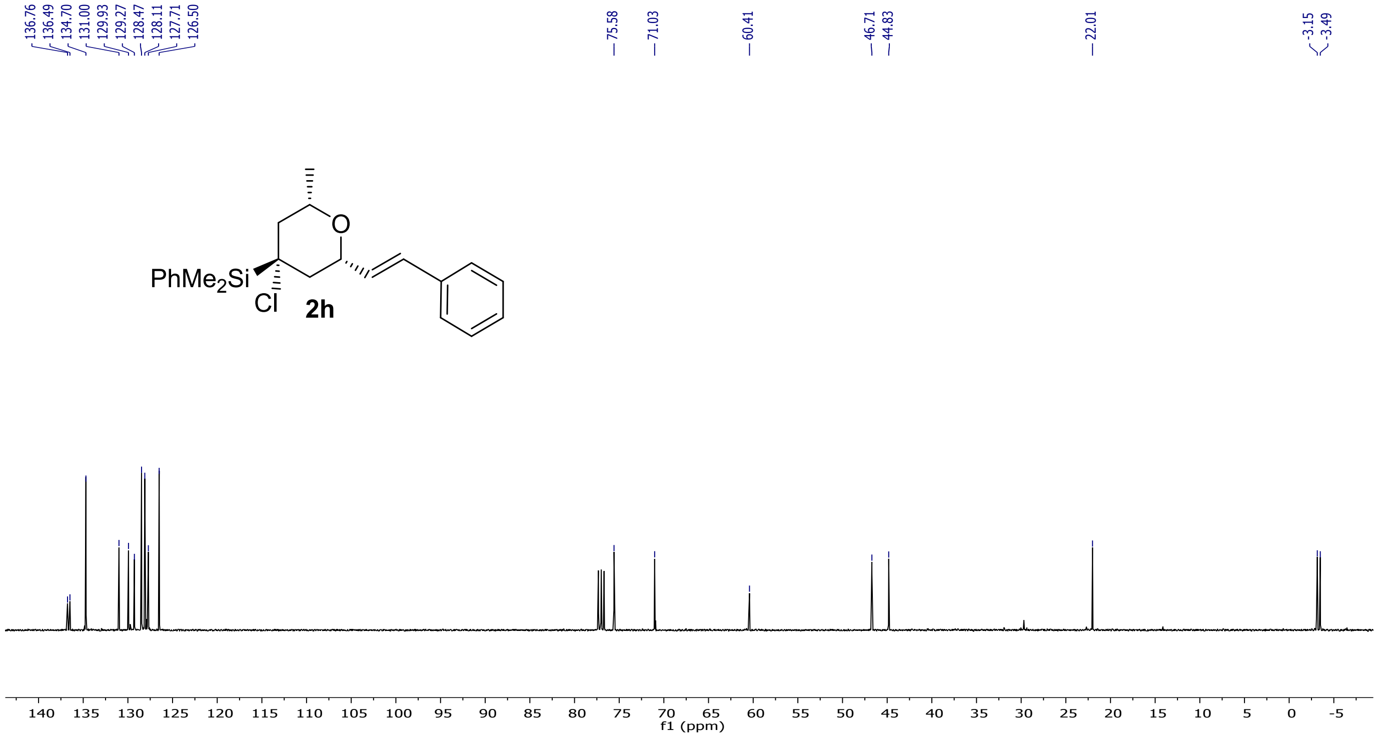

2D-COSY of compound 2h

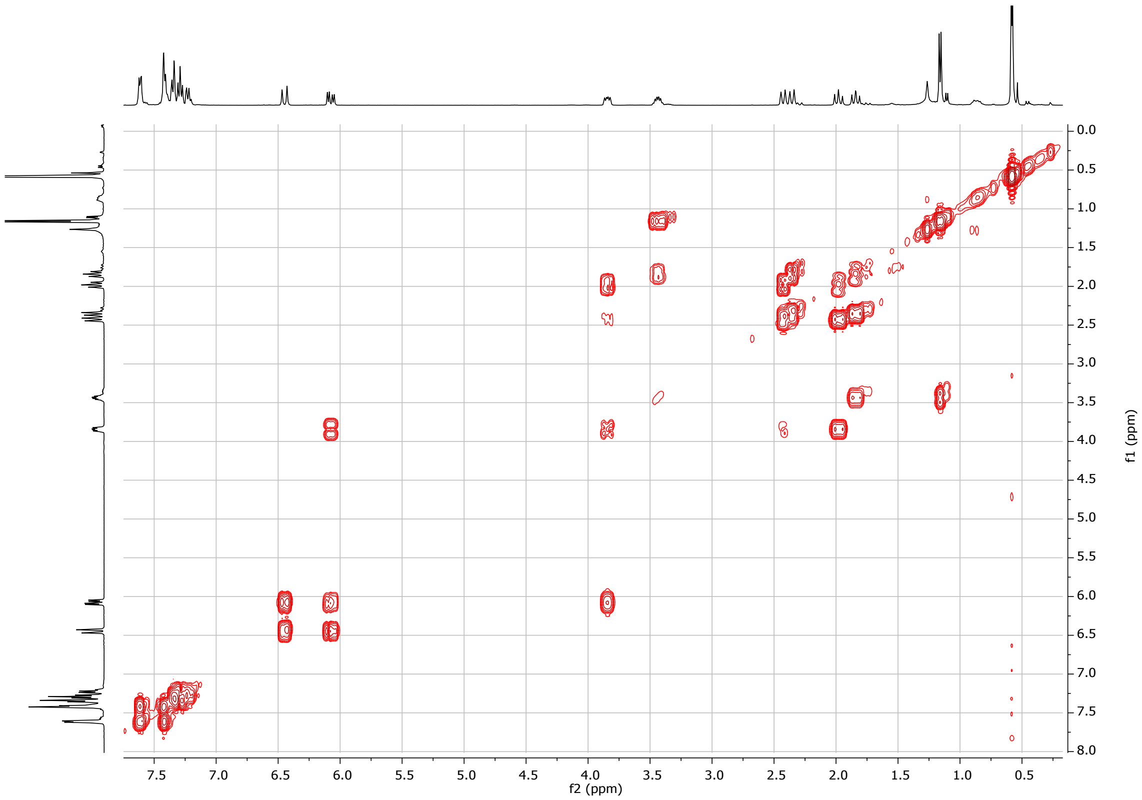

2D-HMBC of compound 2h

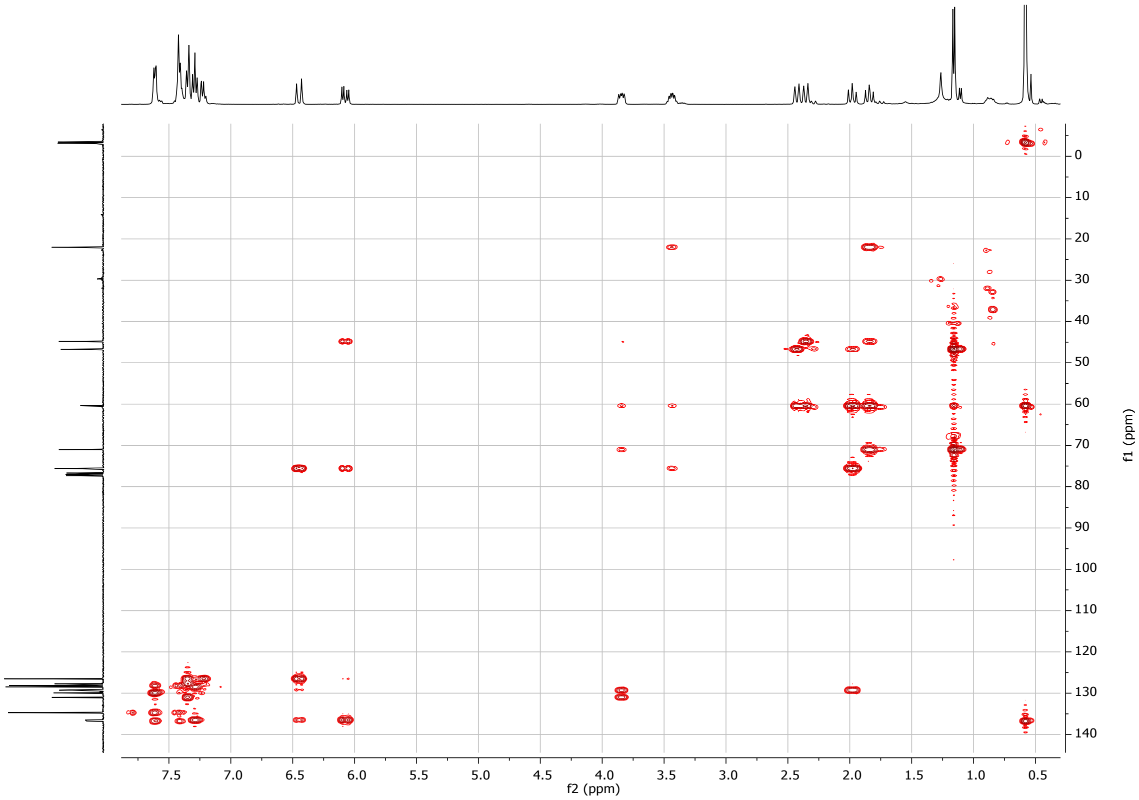

2D-NOESY of compound 2h

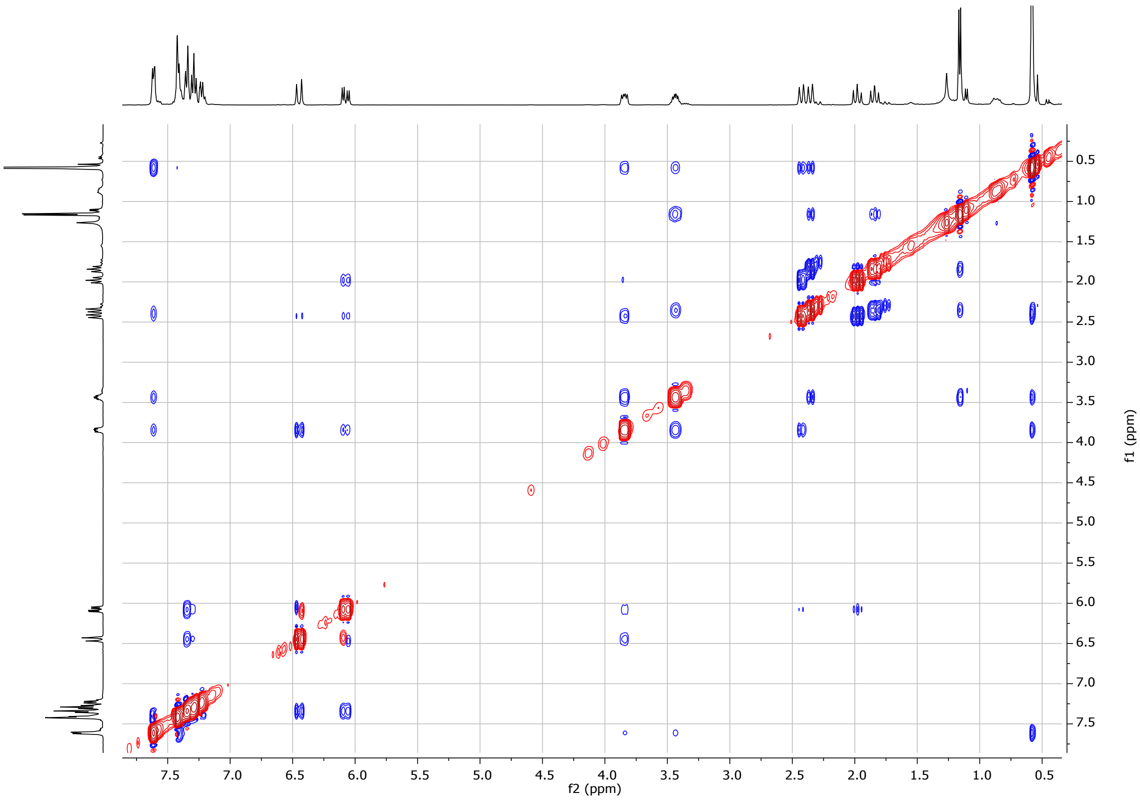

^{1}H NMR (400 MHz, $CDCl_3$)

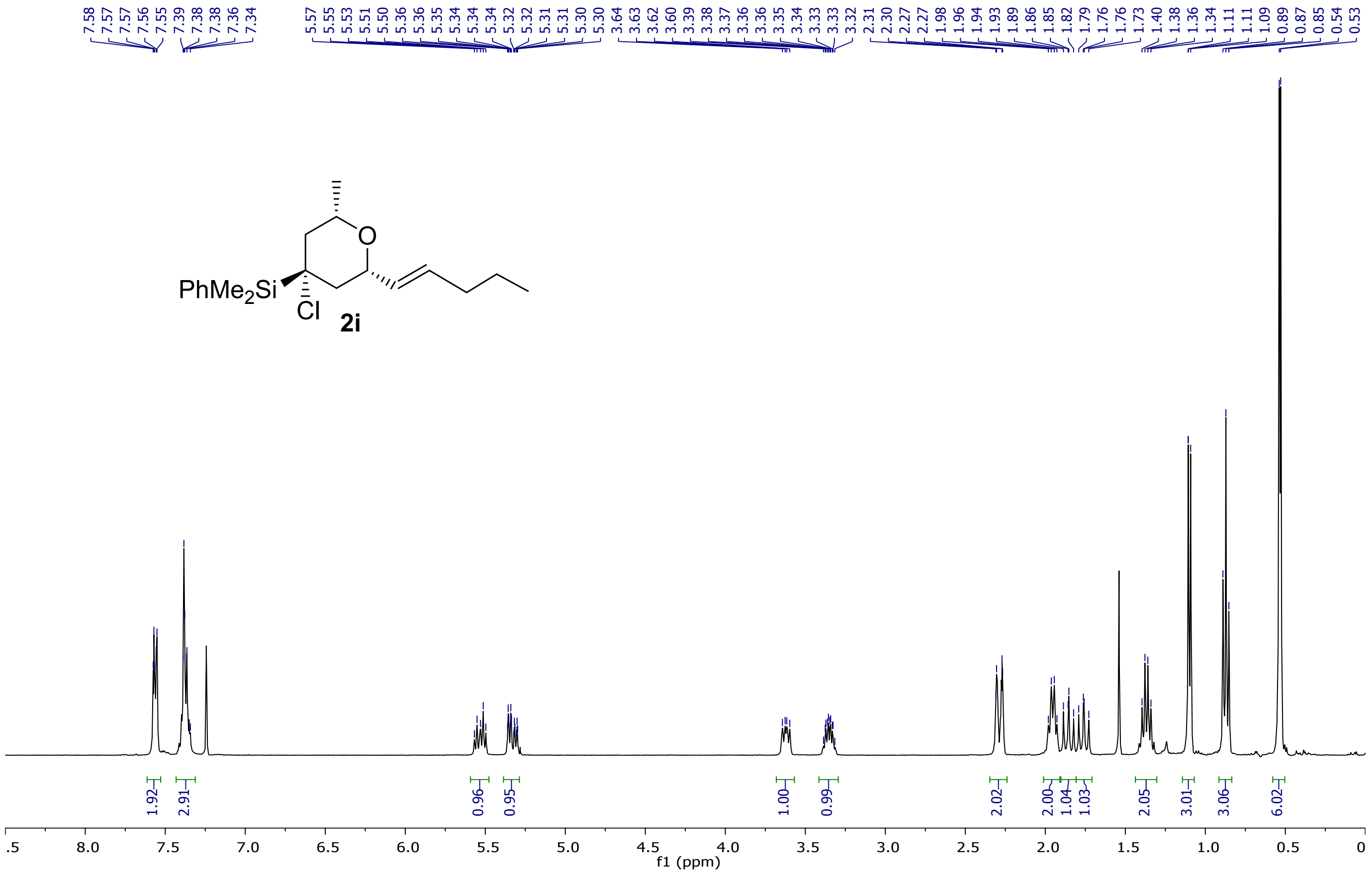

^{13}C {^{1}H} NMR (101 MHz, $CDCl_3$)

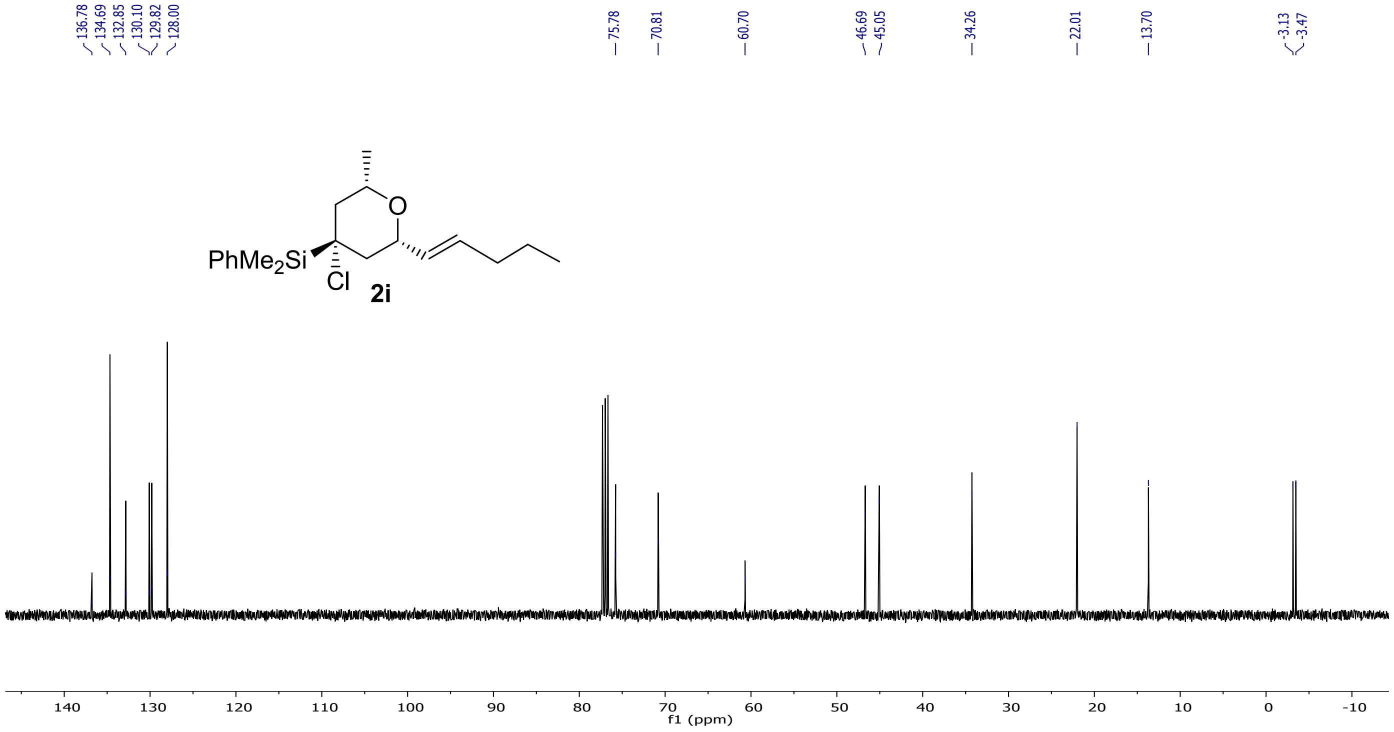

2D-COSY of compound 2i

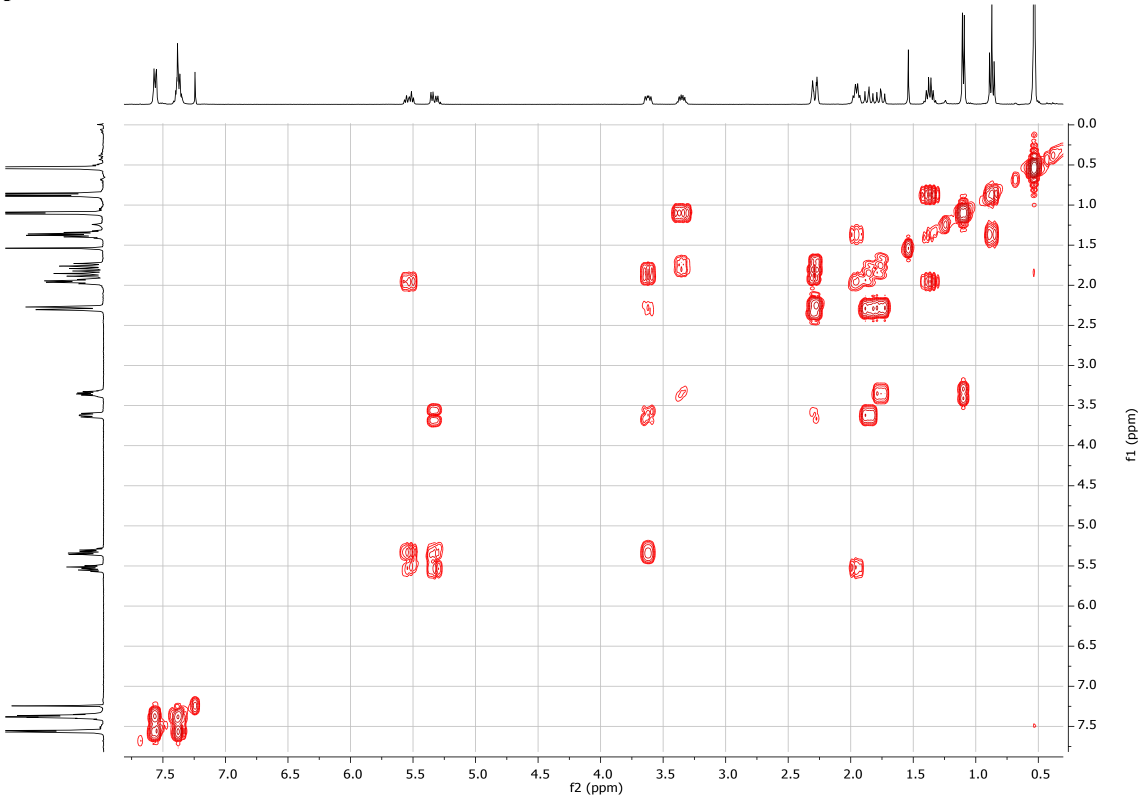

2D-HMBC of compound 2i

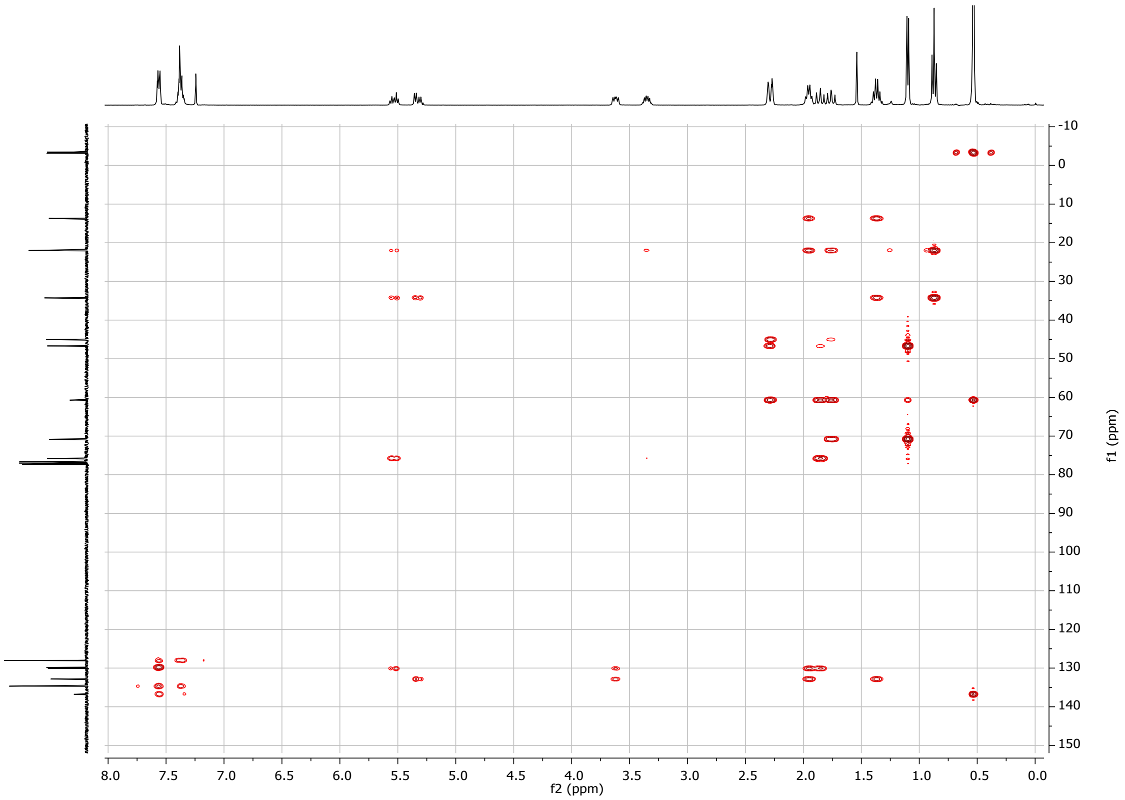

2D-NOESY of compound 2i

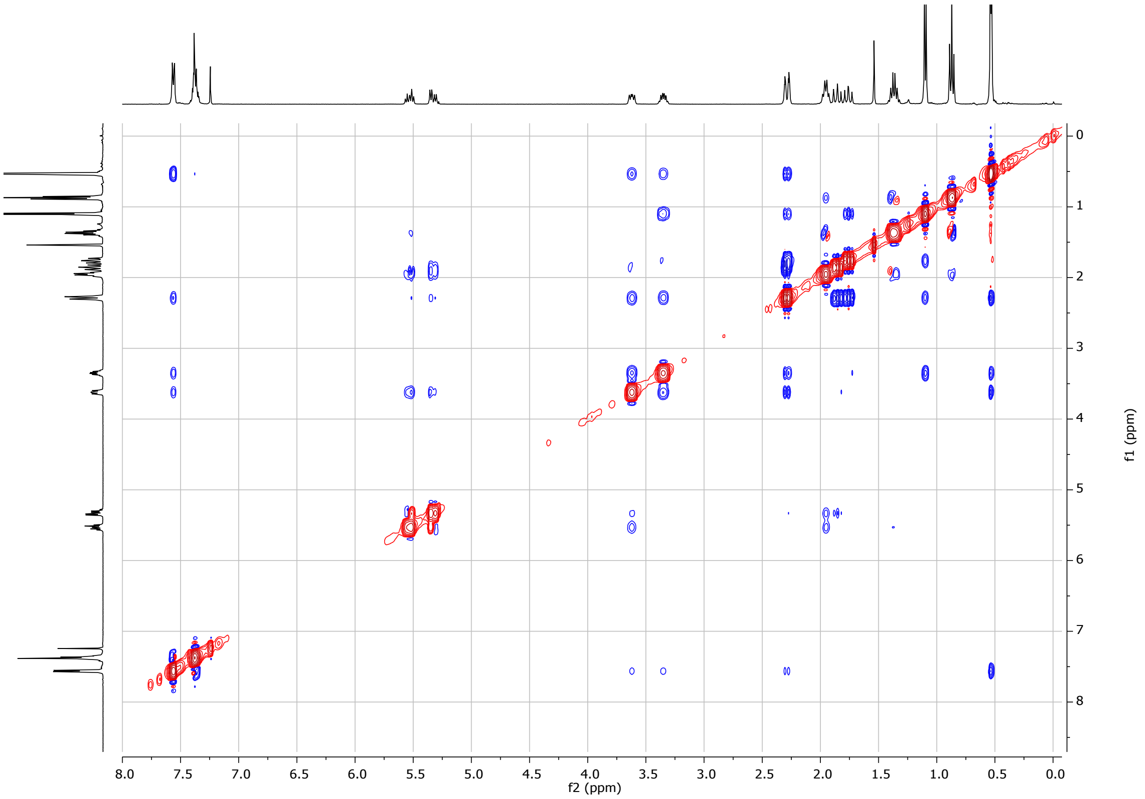

^{1}H NMR (400 MHz, $CDCl_3$)

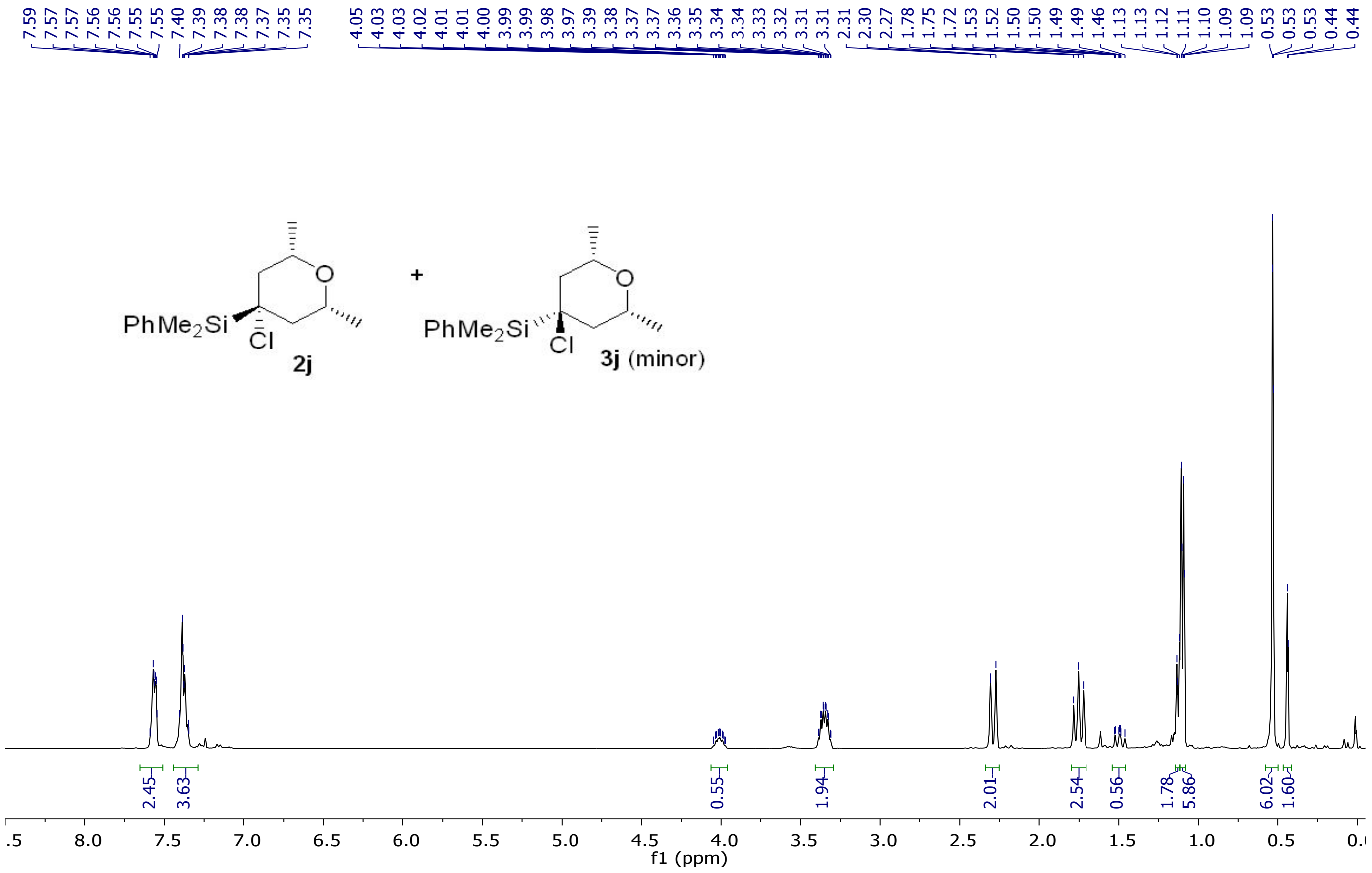

^{13}C {^{1}H} NMR (101 MHz, $CDCl_3$)

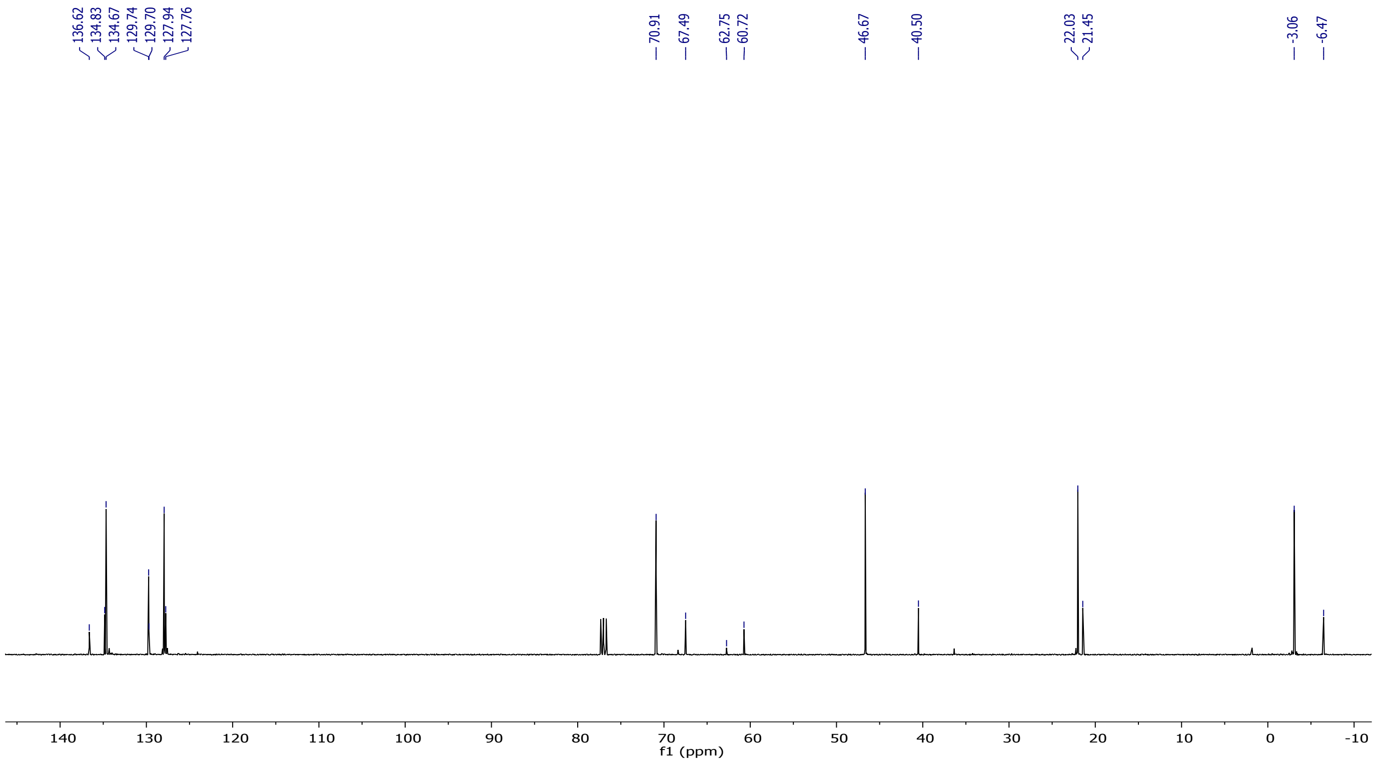

2D-COSY of compound 2j

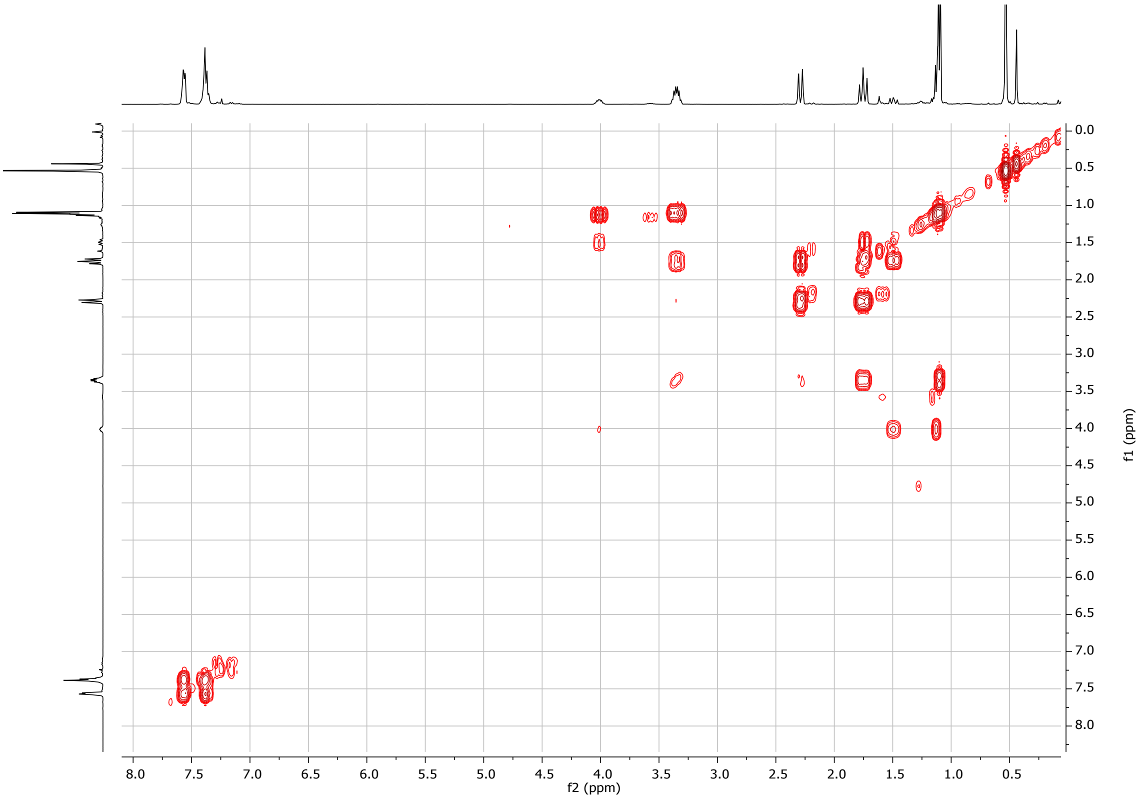

2D-NOESY of compound 2j

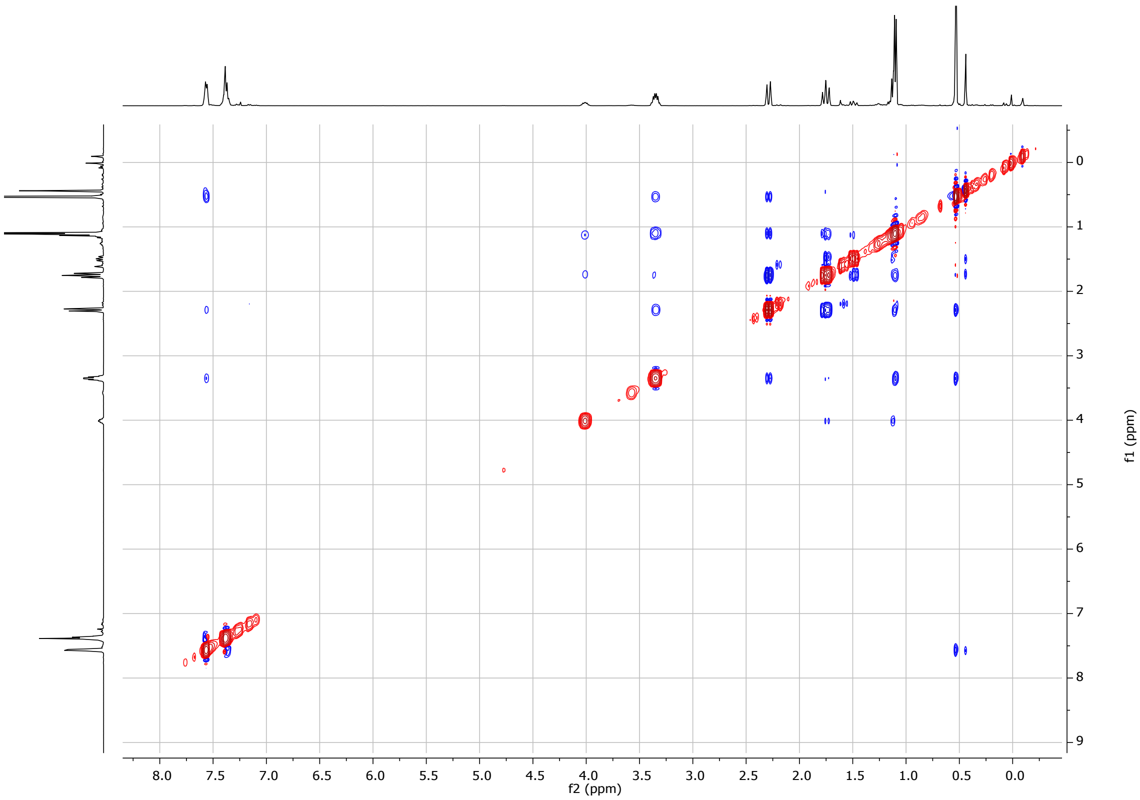

^{1}H NMR (400 MHz, $CDCl_3$)

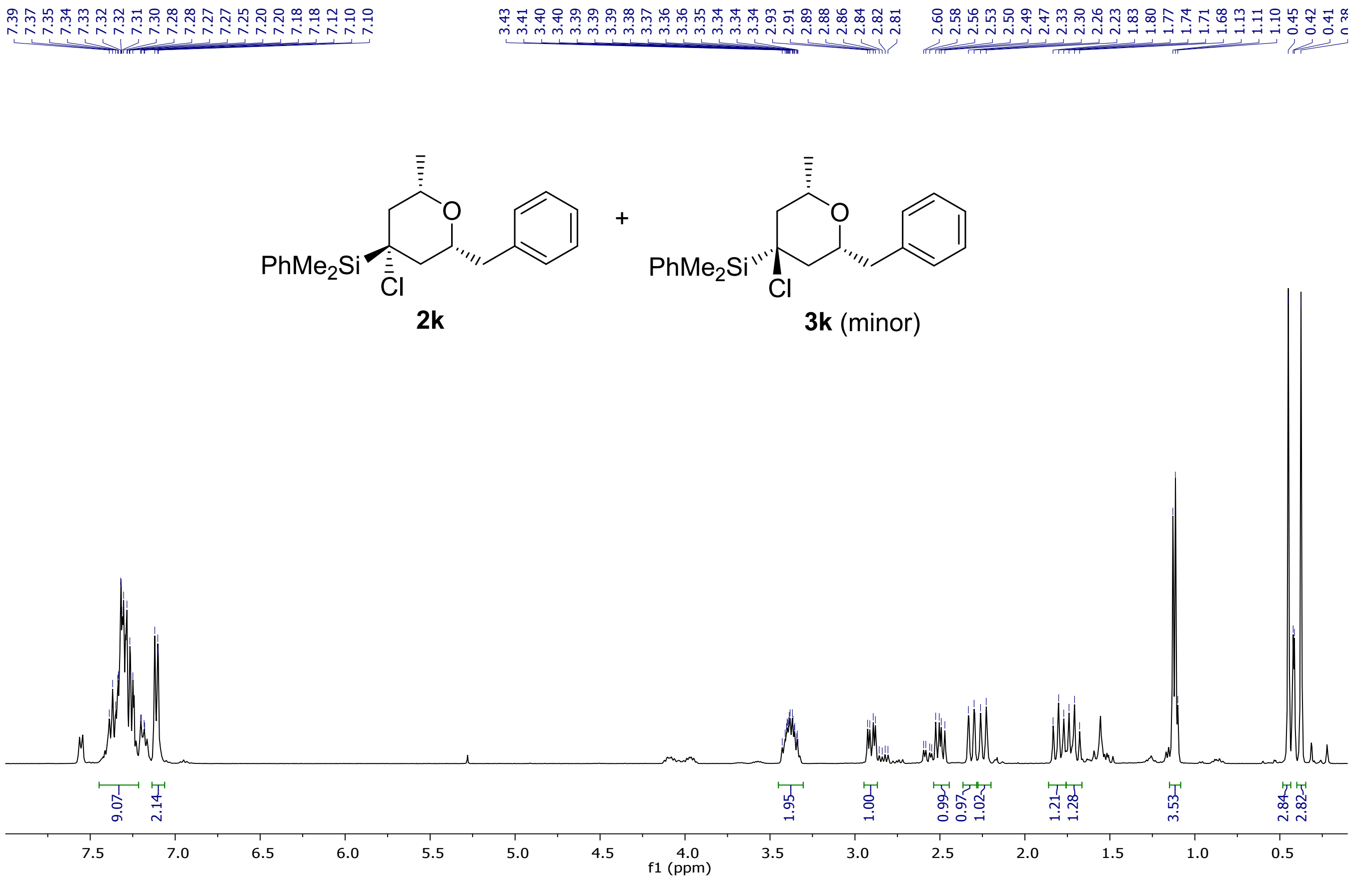

^{13}C {^{1}H} NMR (101 MHz, $CDCl_3$)

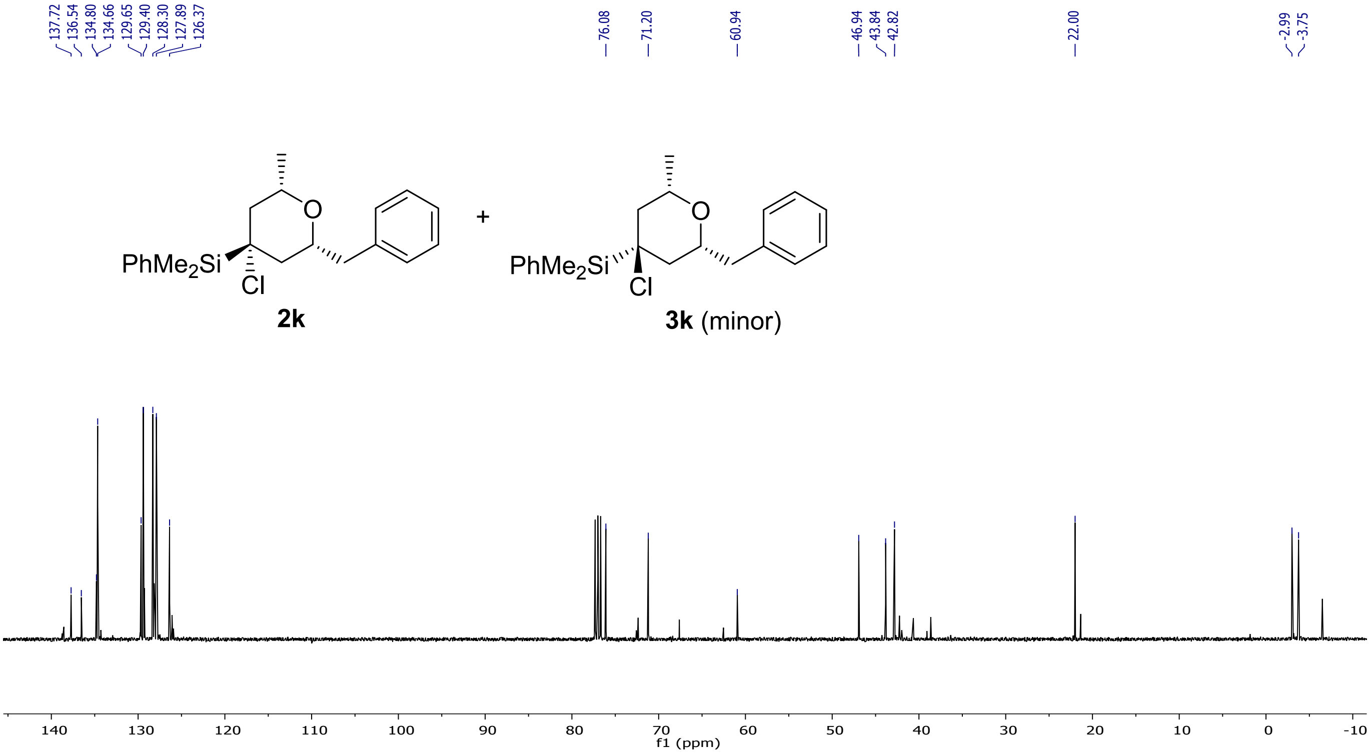

2D-COSY of compound 2k

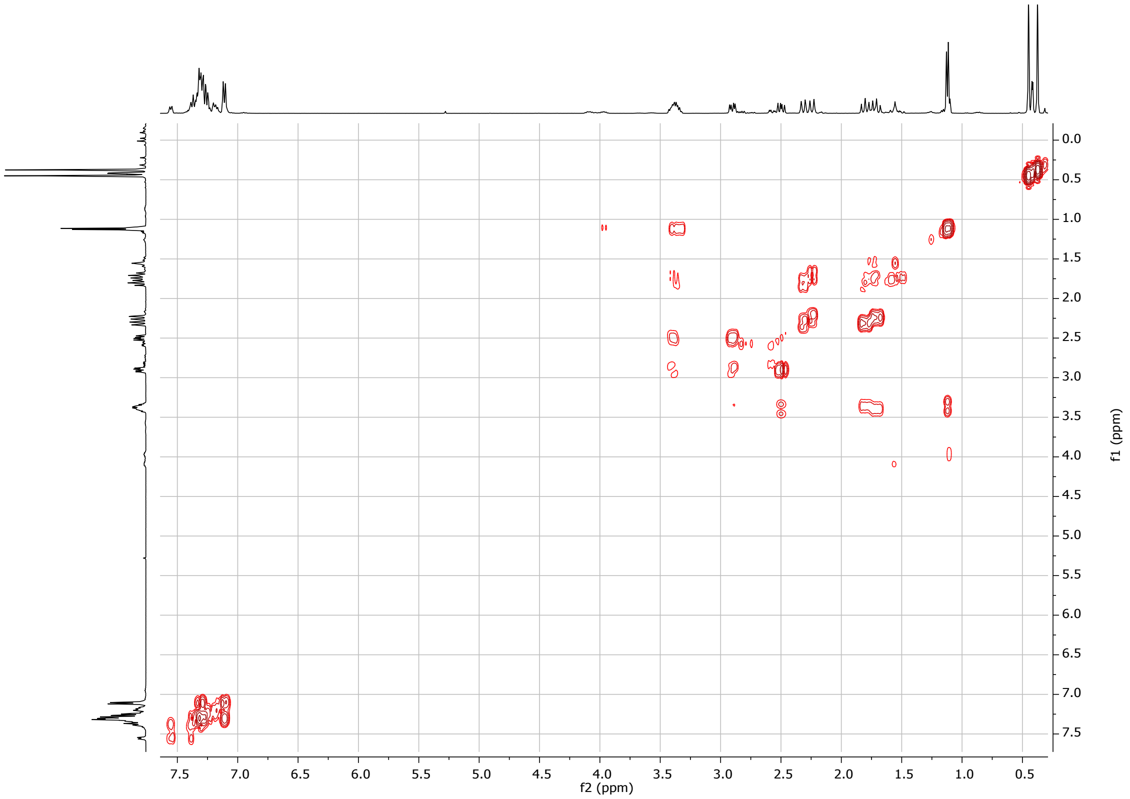

2D-NOESY of compound 2k

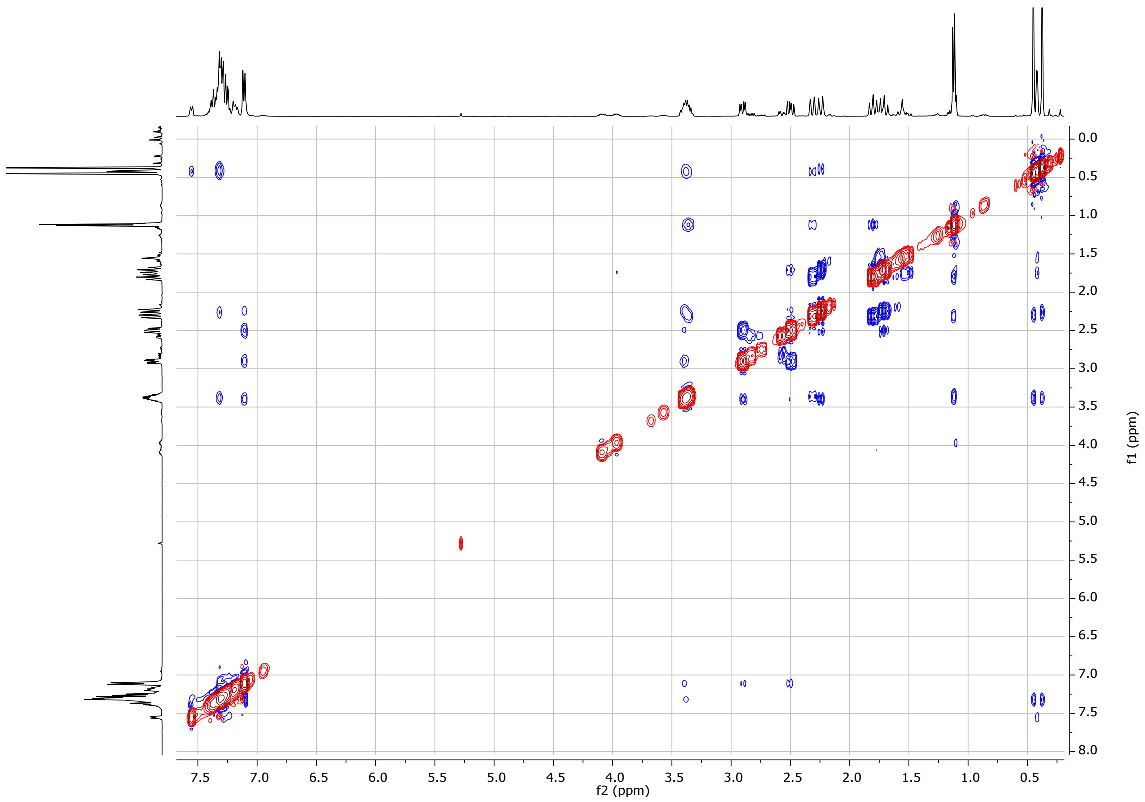

^{1}H NMR (400 MHz, $CDCl_3$)

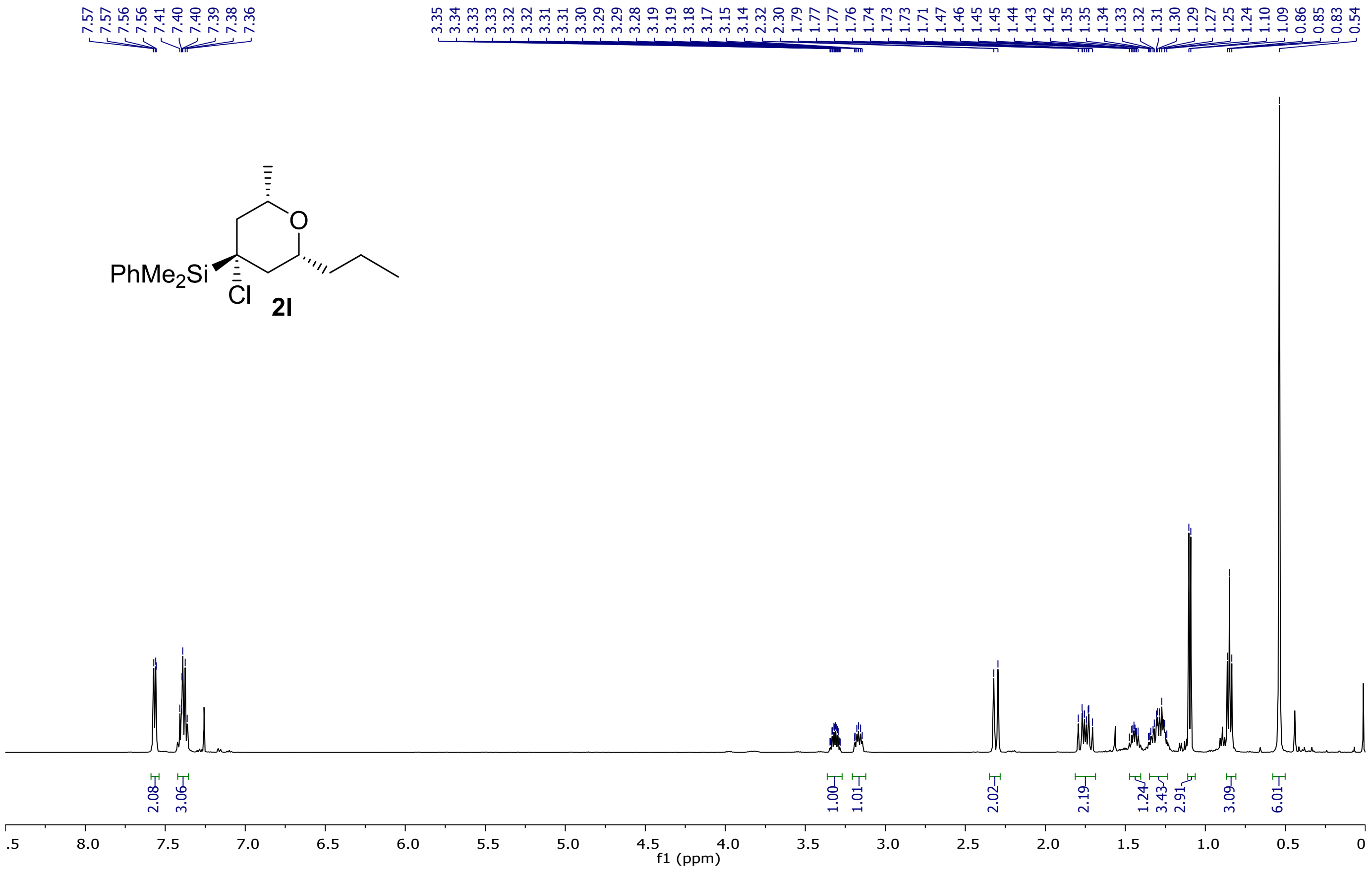

^{13}C {^{1}H} NMR (101 MHz, $CDCl_3$)

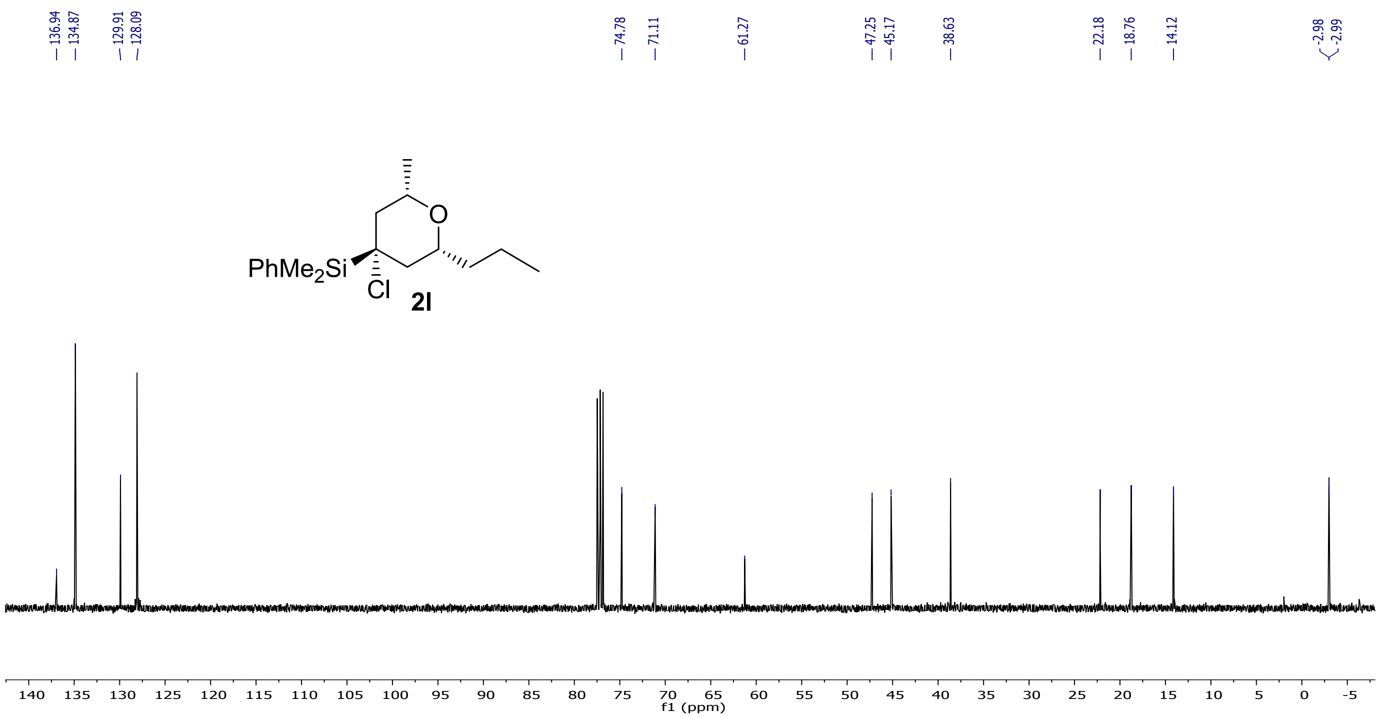

2D-COSY of compound 2l

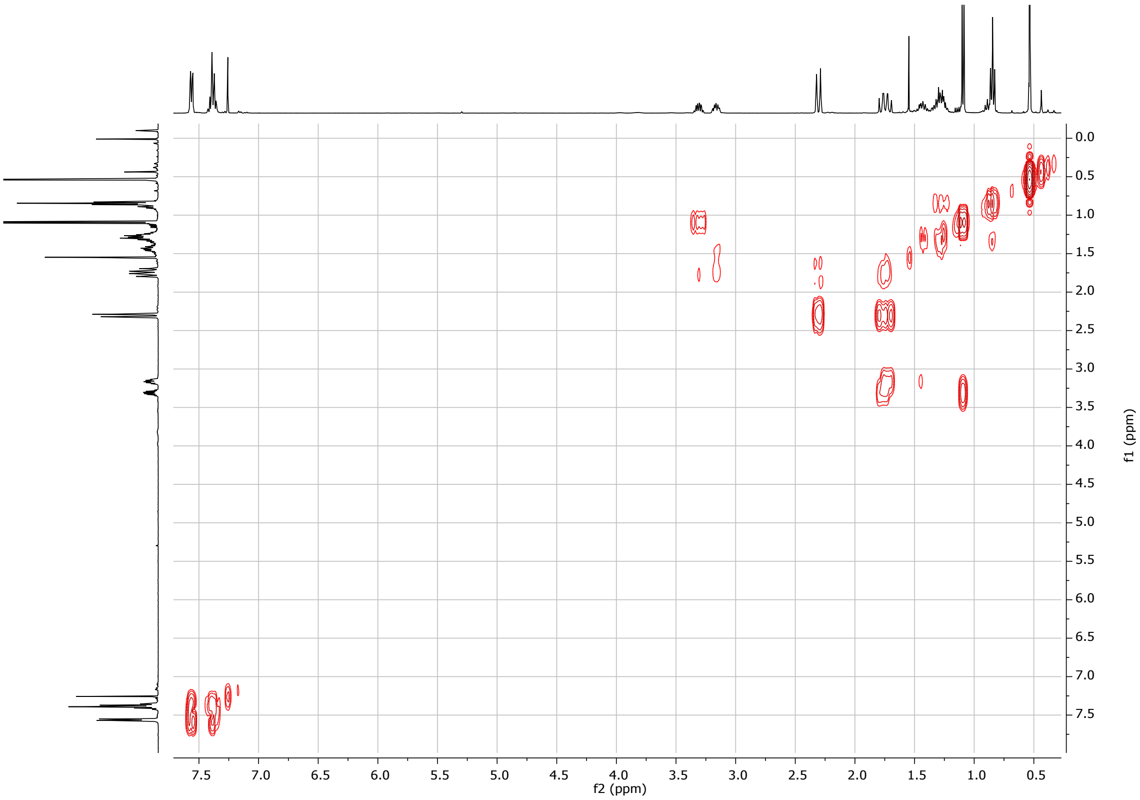

2D-NOESY of compound 2l

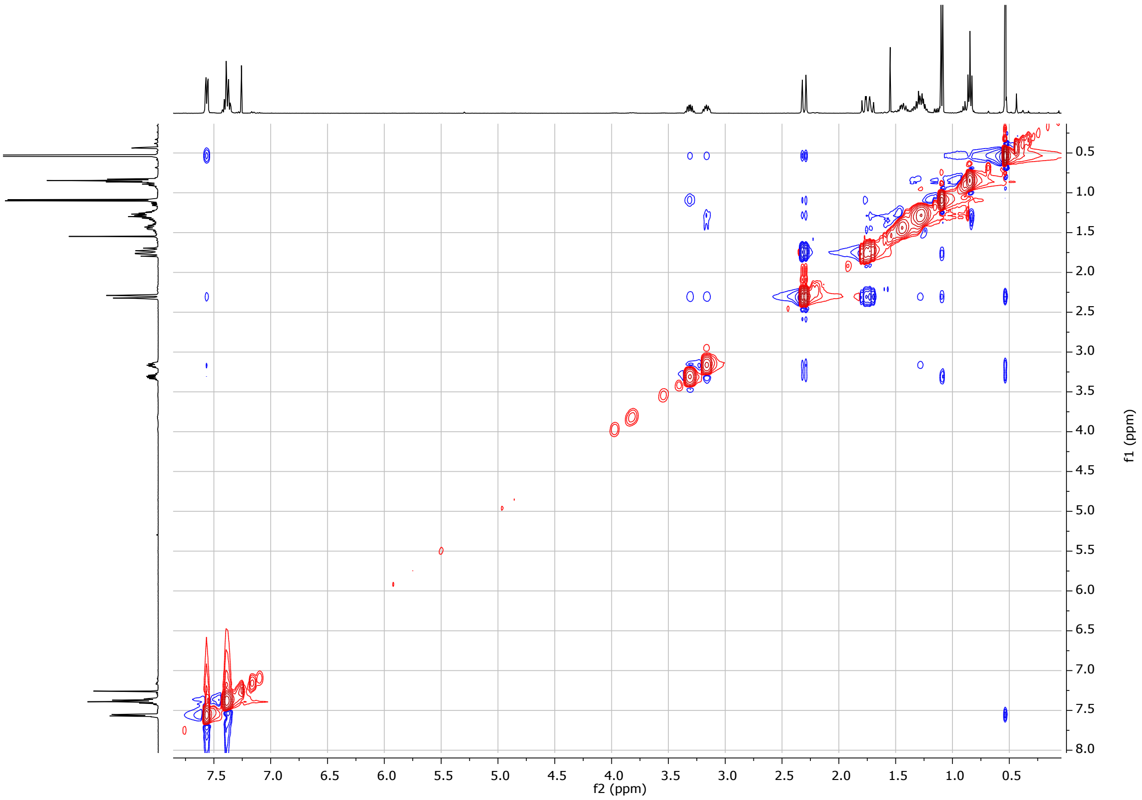

^{1}H NMR (400 MHz, $CDCl_3$)

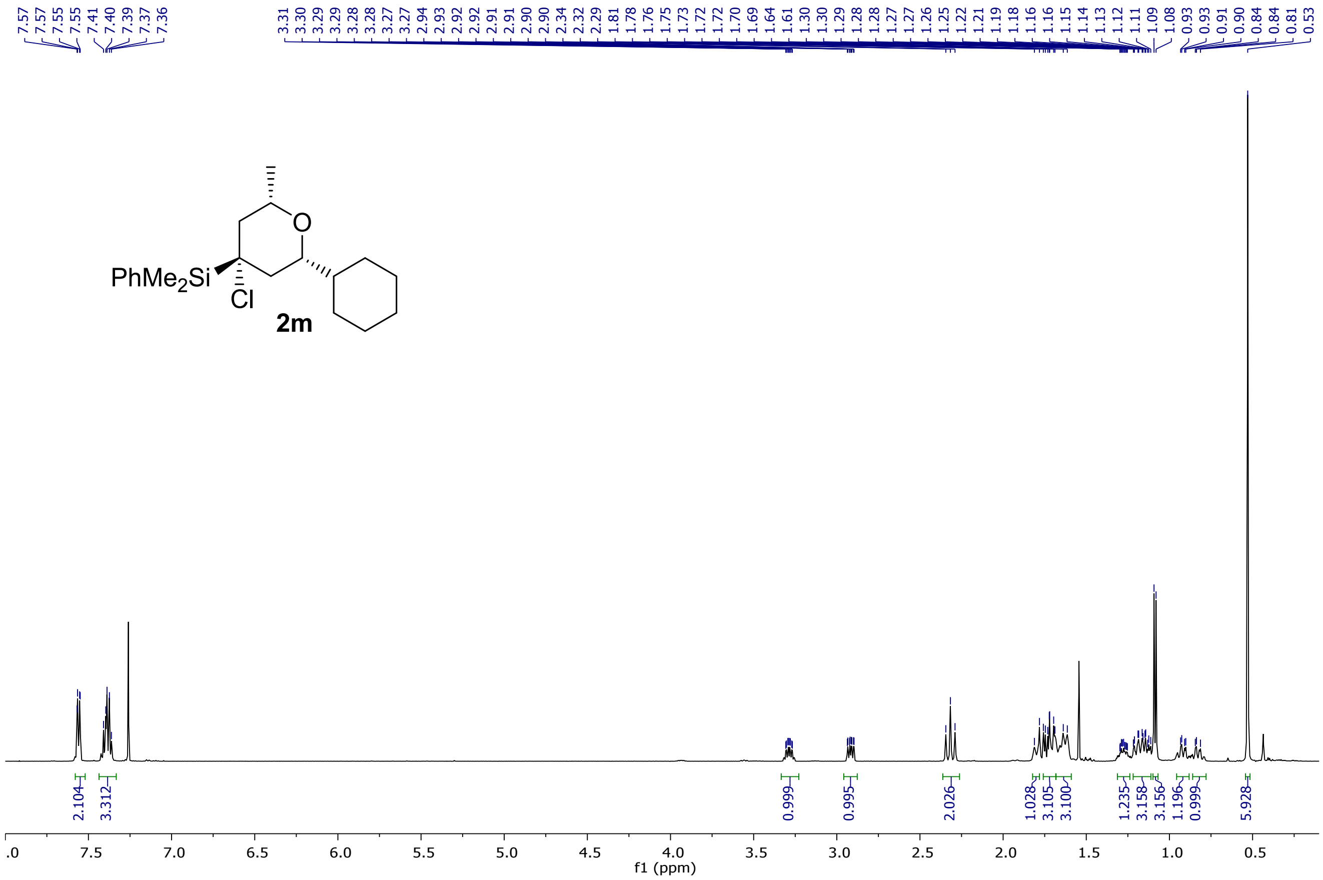

^{13}C {^{1}H} NMR (101 MHz, $CDCl_3$)

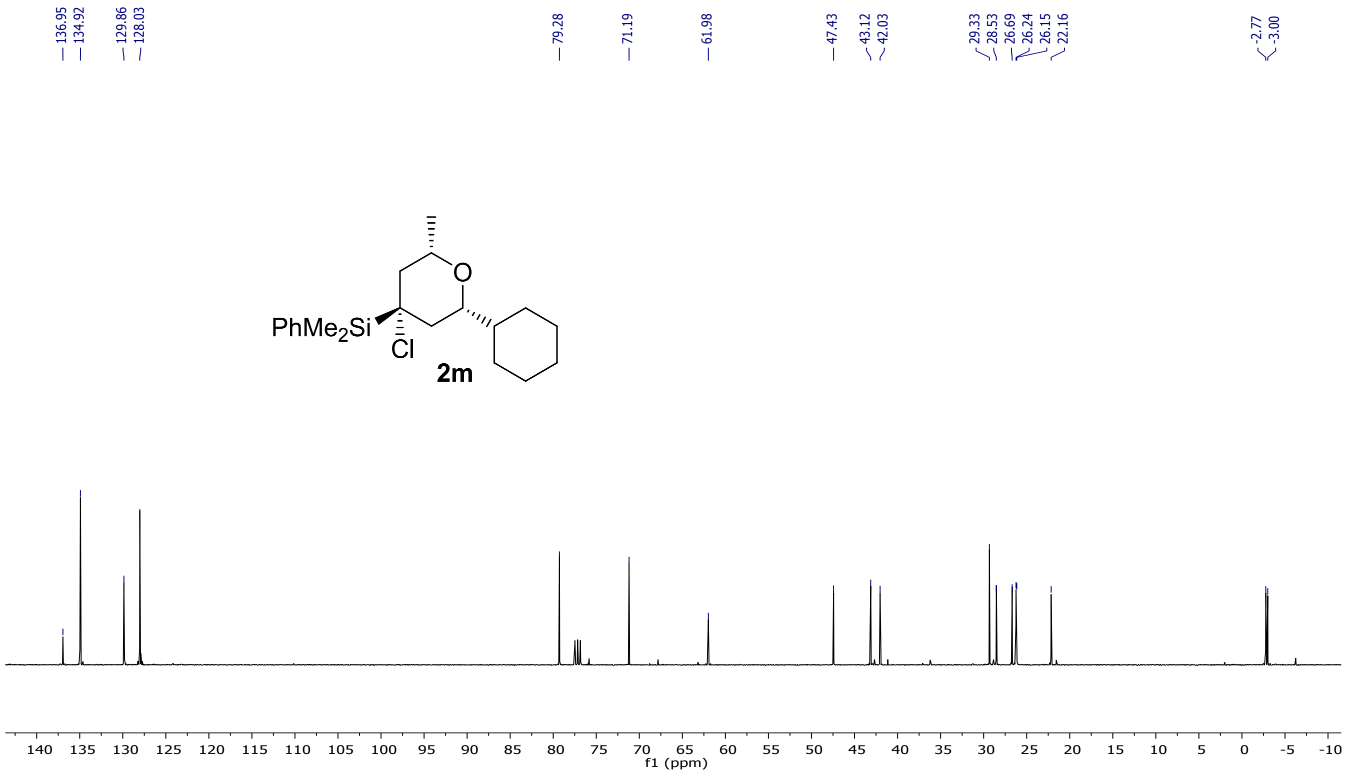

2D-COSY of compound 2m

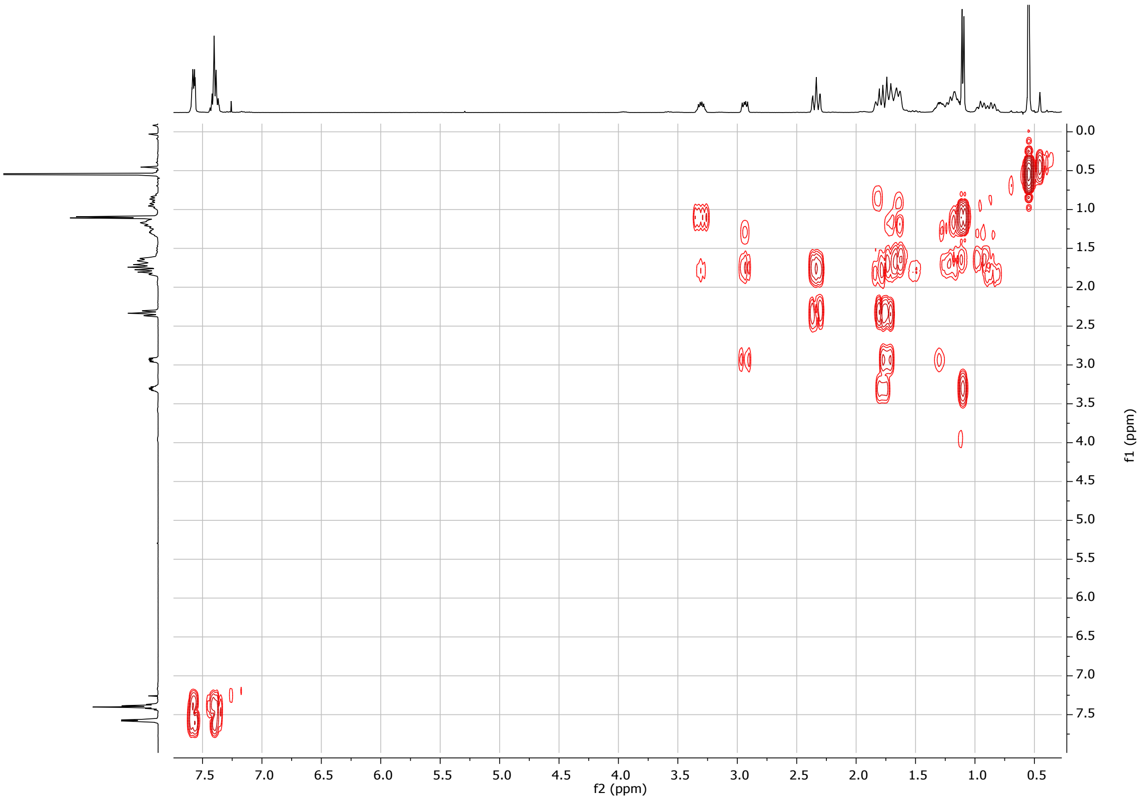

2D-NOESY of compound 2m

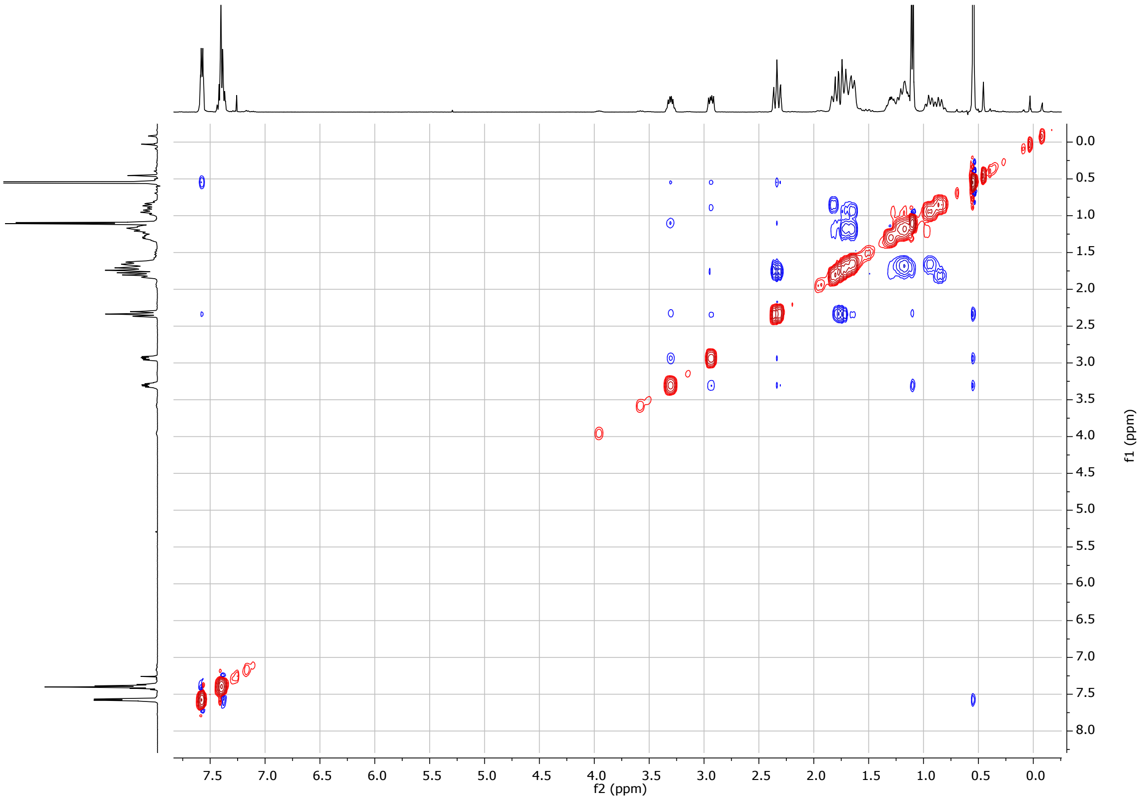

^{1}H NMR (400 MHz, $CDCl_3$)

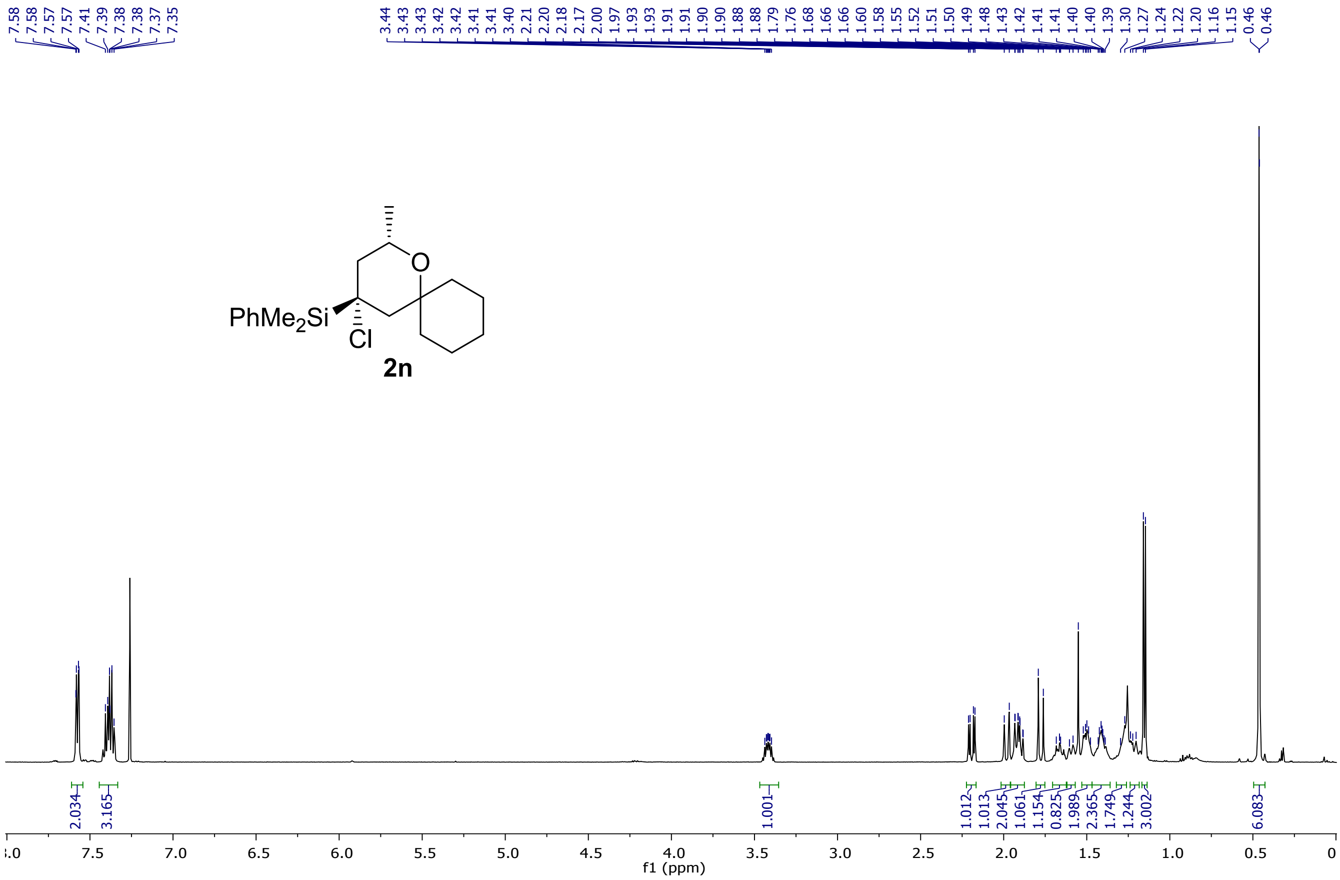

^{13}C {^{1}H} NMR (101 MHz, $CDCl_3$)

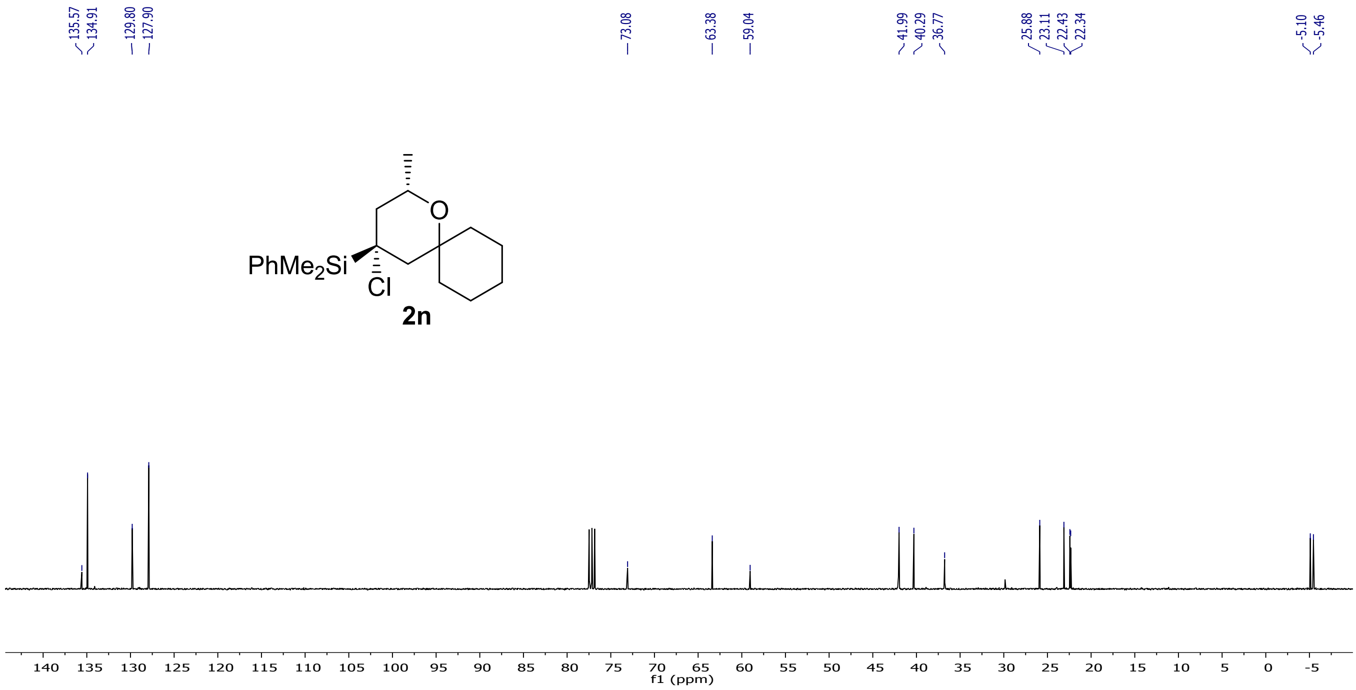

2D-COSY of compound 2n

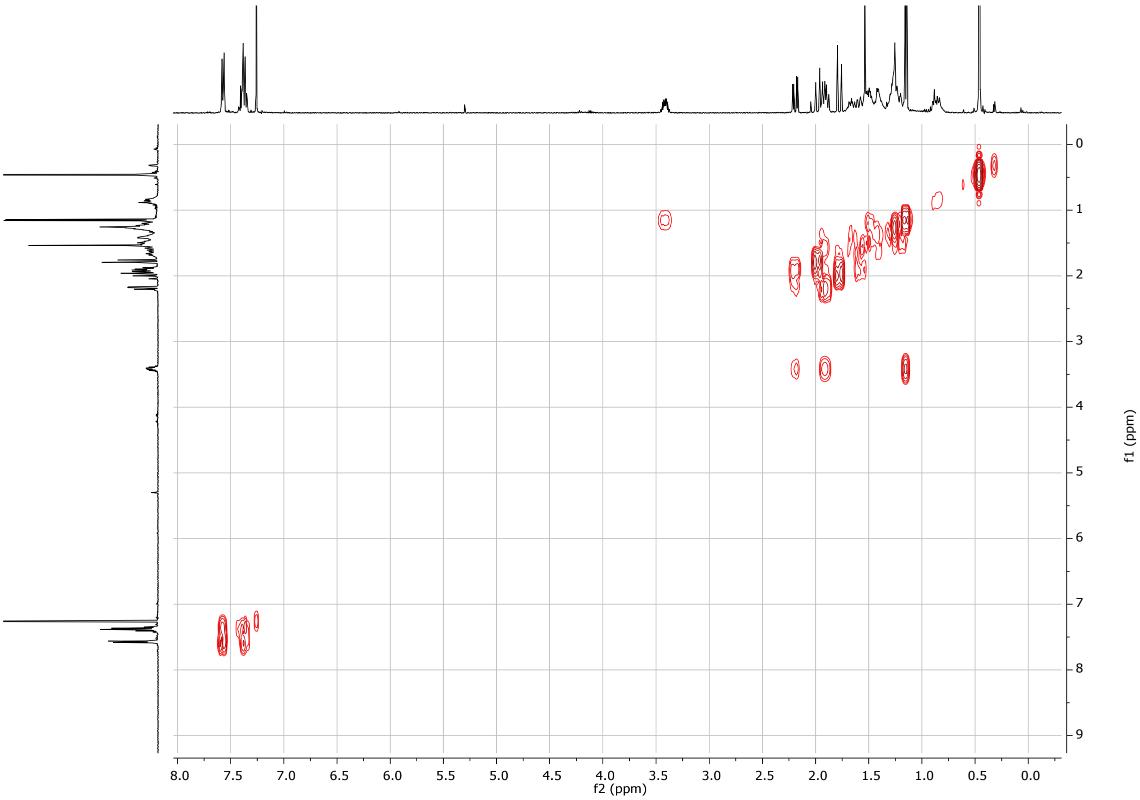

2D-NOESY of compound 2n

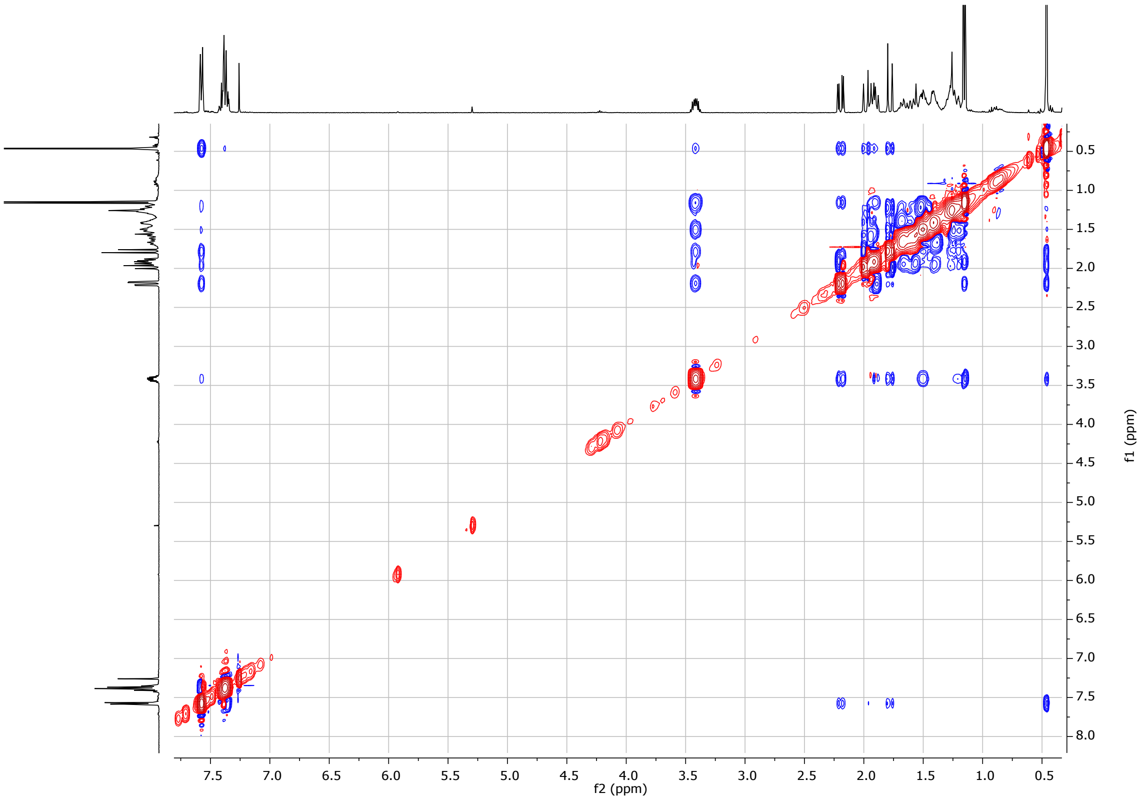

^{1}H NMR (400 ^{1}H NMR (400 ^{1}H NMR (400 MHz, $CDCl_3$)

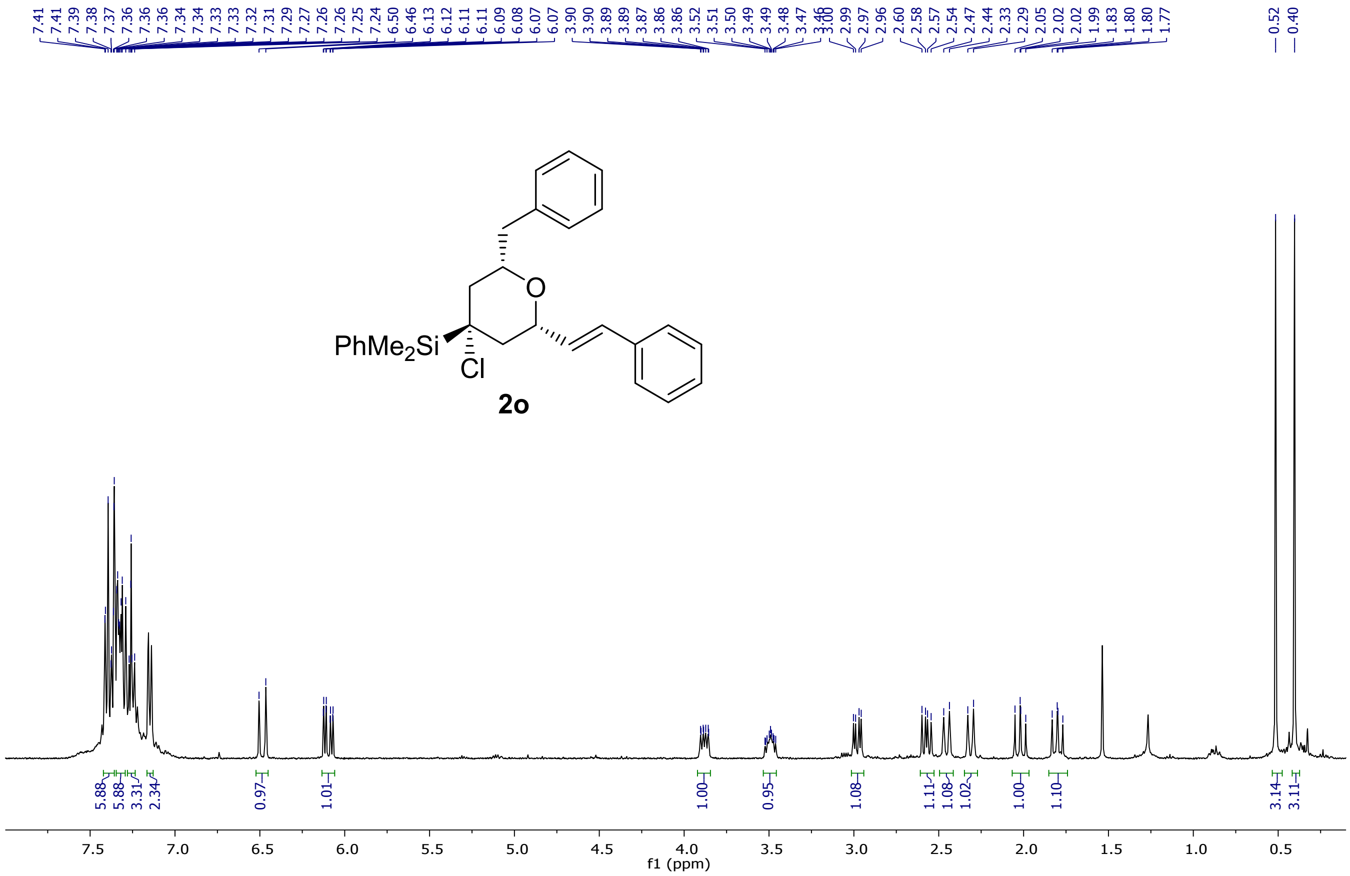

^{13}C {^{1}H} NMR (101 MHz, $CDCl_3$)

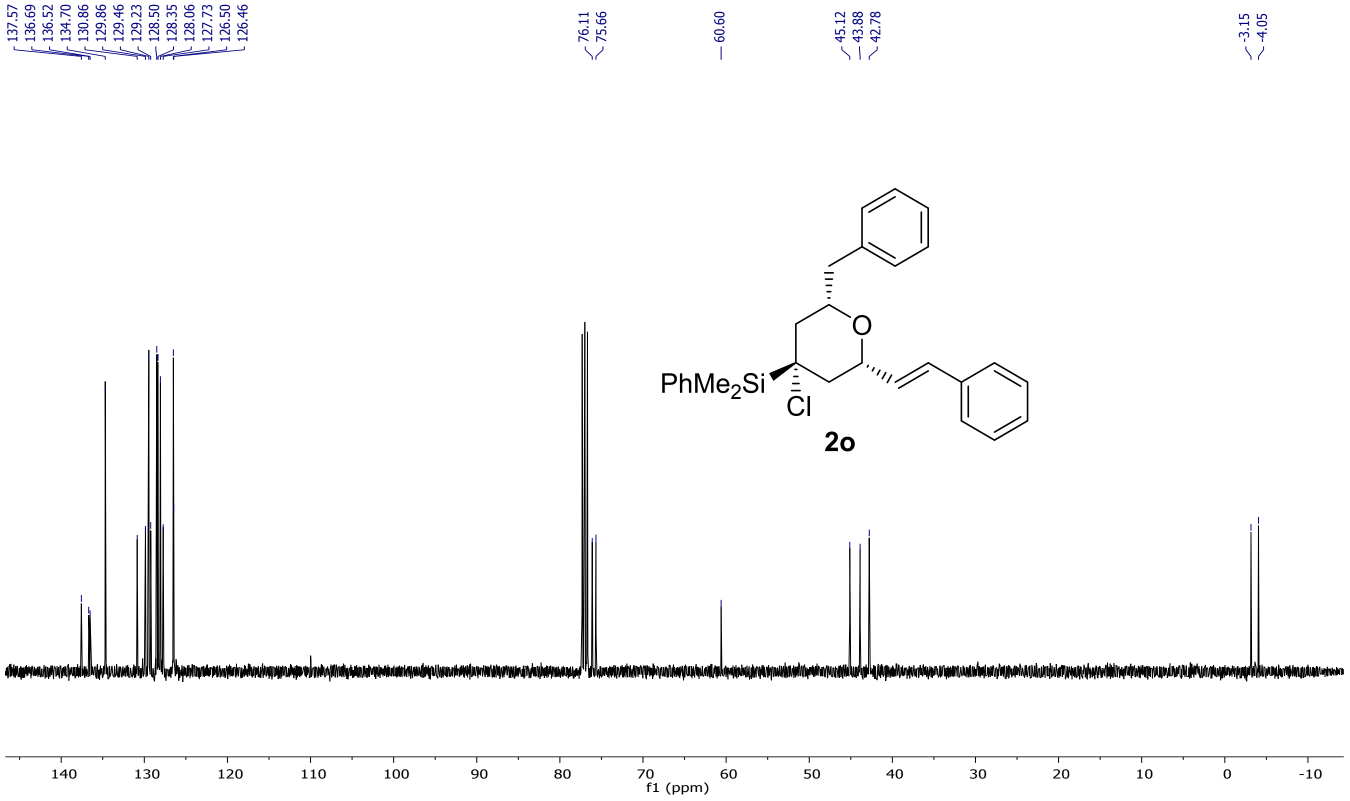

2D-COSY of compound 2o

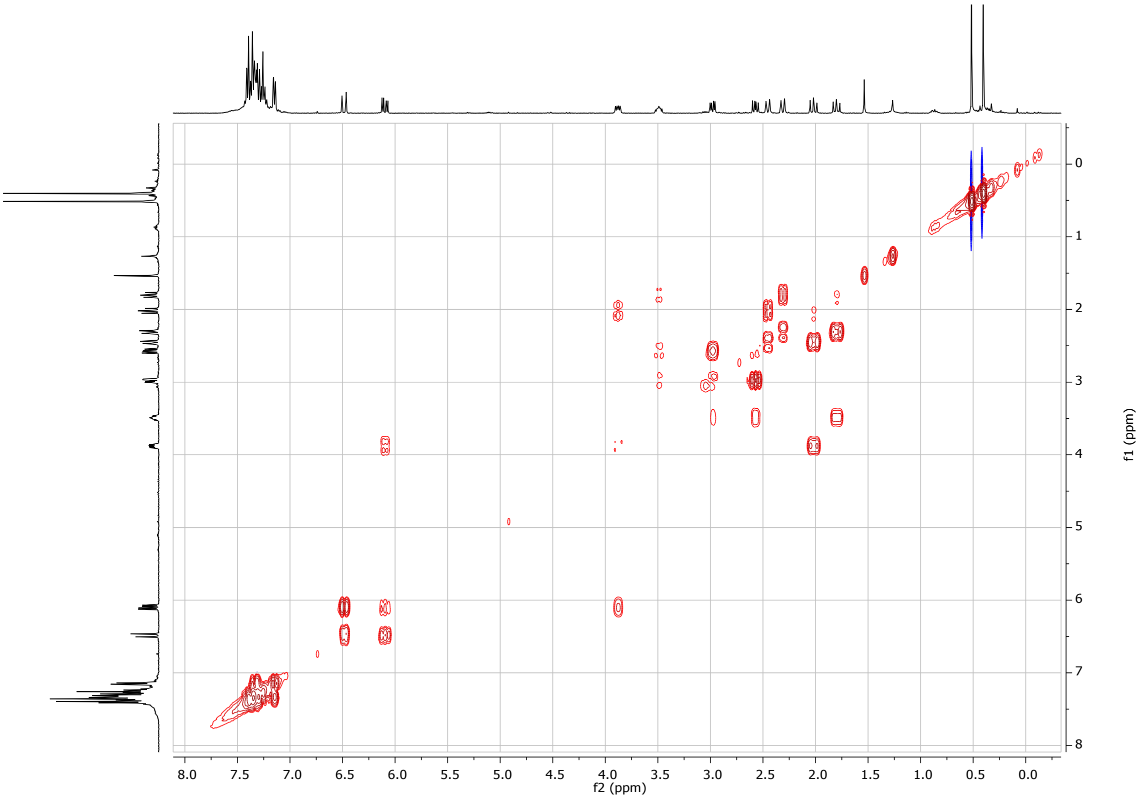

2D-HMBC of compound 2o

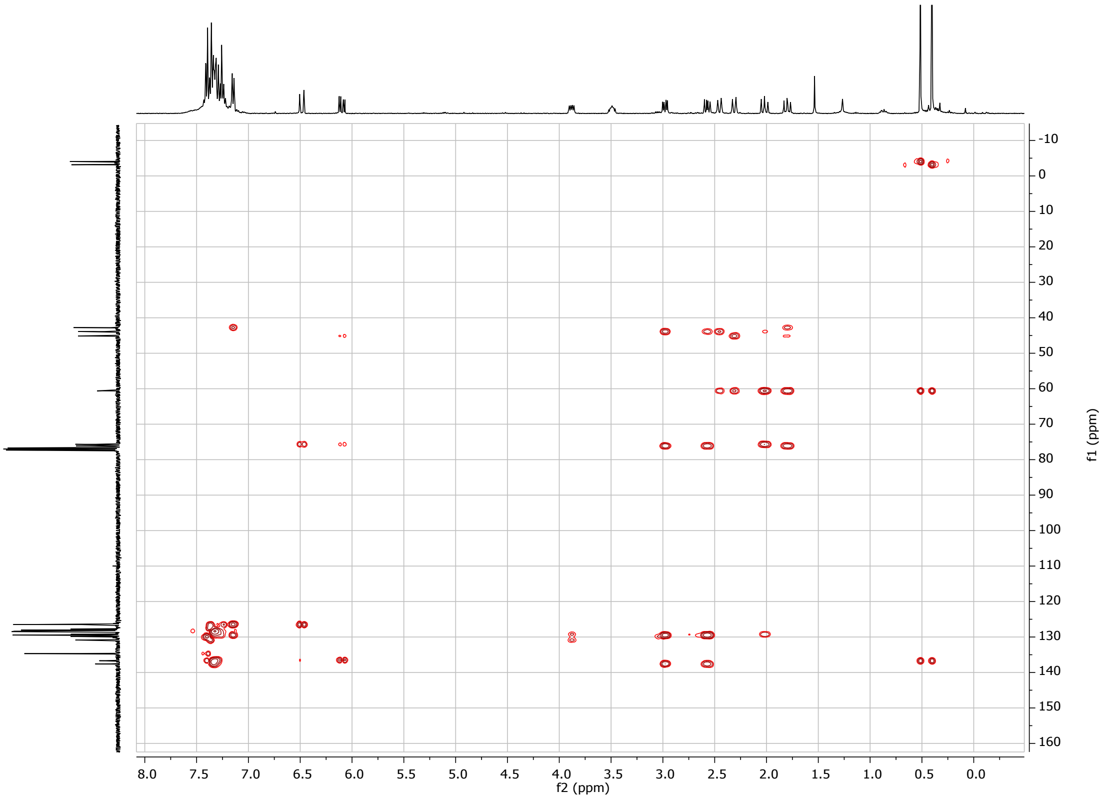

2D-NOESY of compound 2o

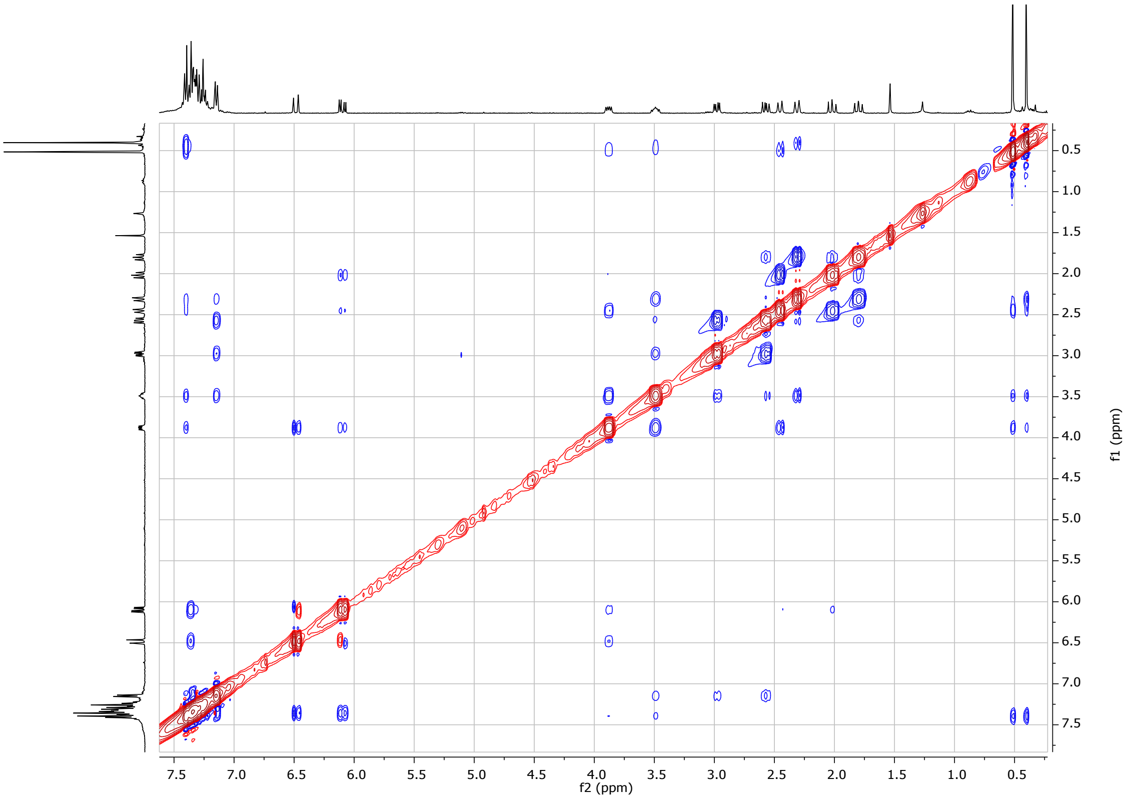

^{1}H NMR (400 MHz, $CDCl_3$)

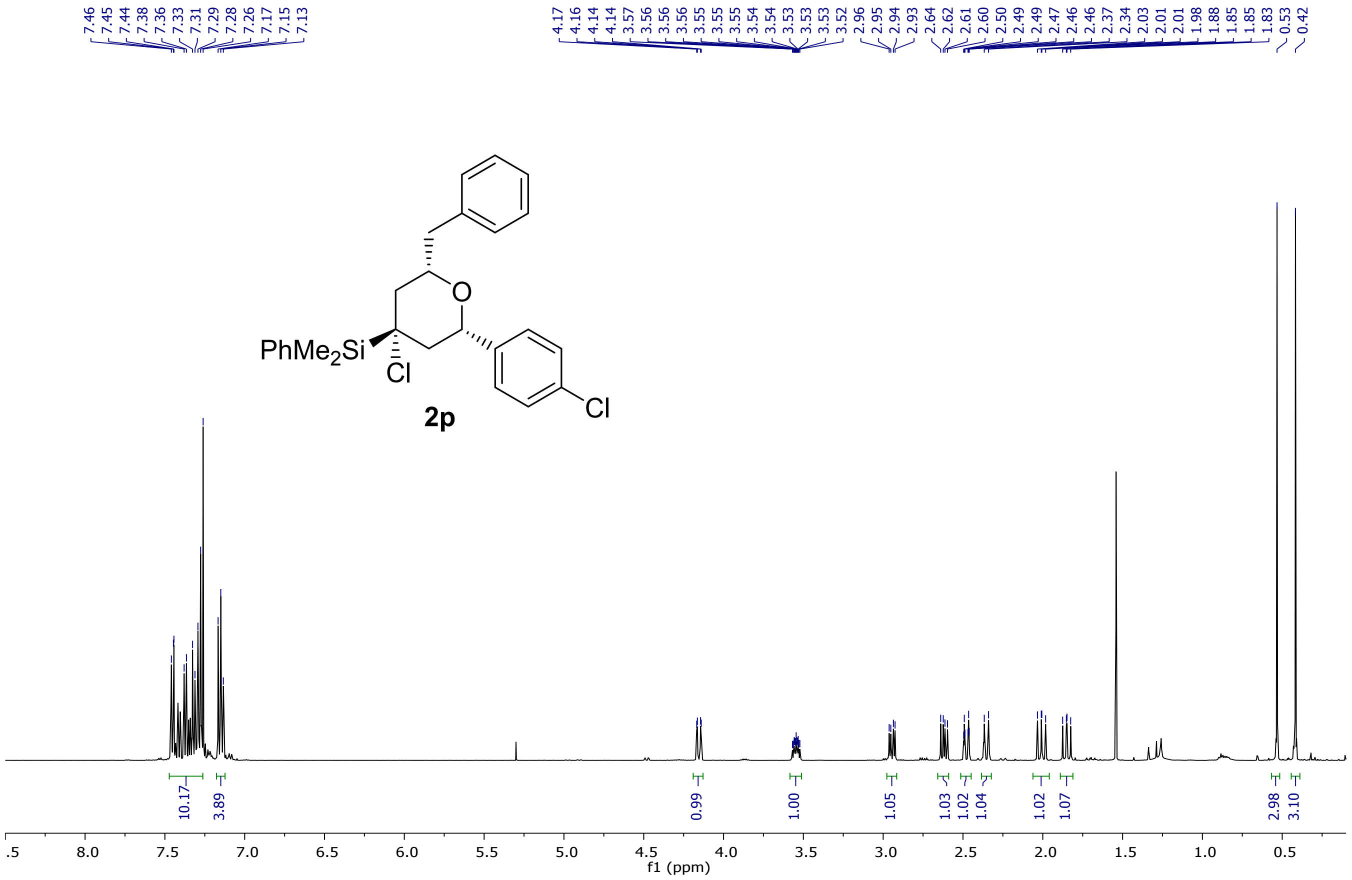

^{13}C {^{1}H} NMR (101 MHz, $CDCl_3$)

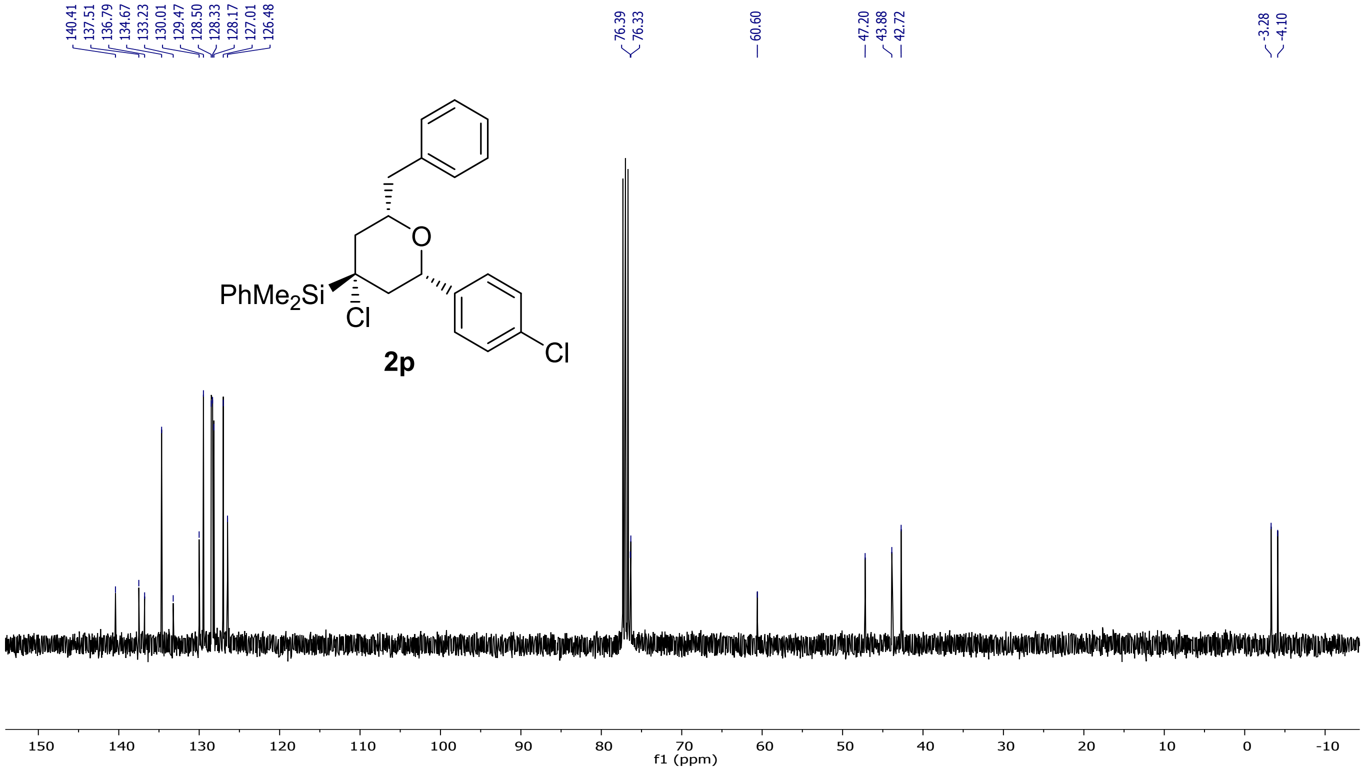

2D-COSY of compound 2p

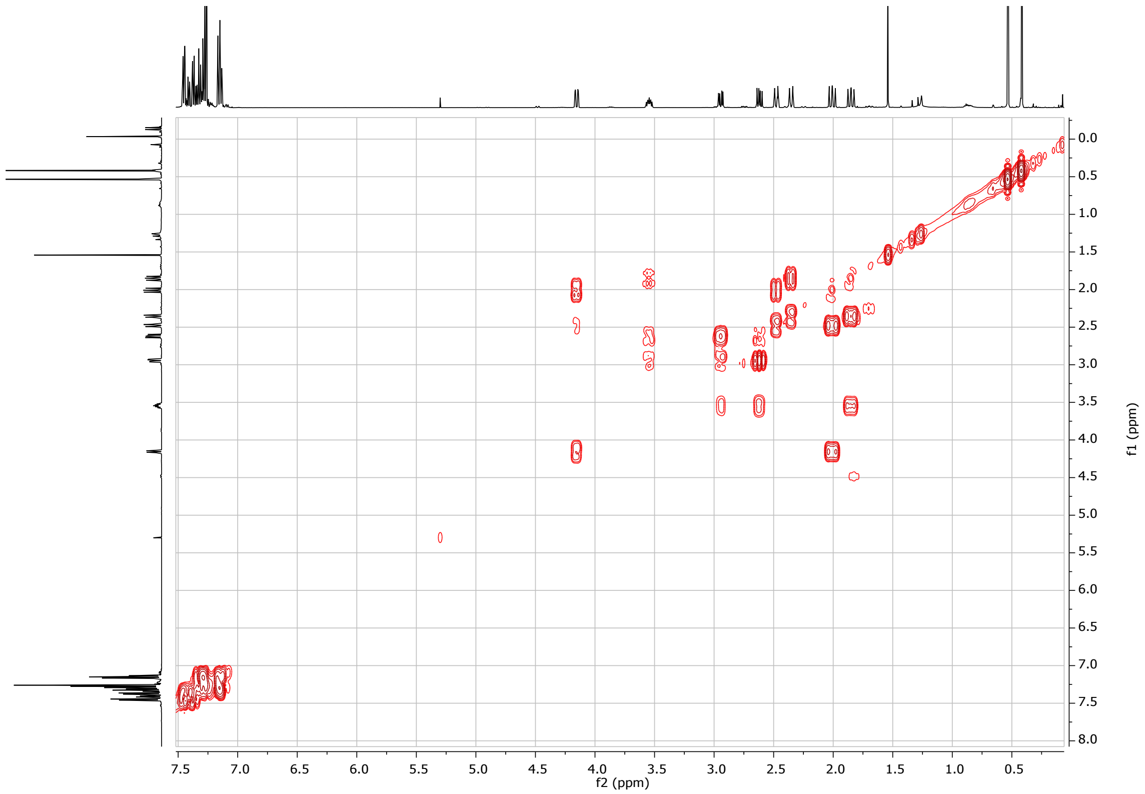

2D-HMBC of compound 2p

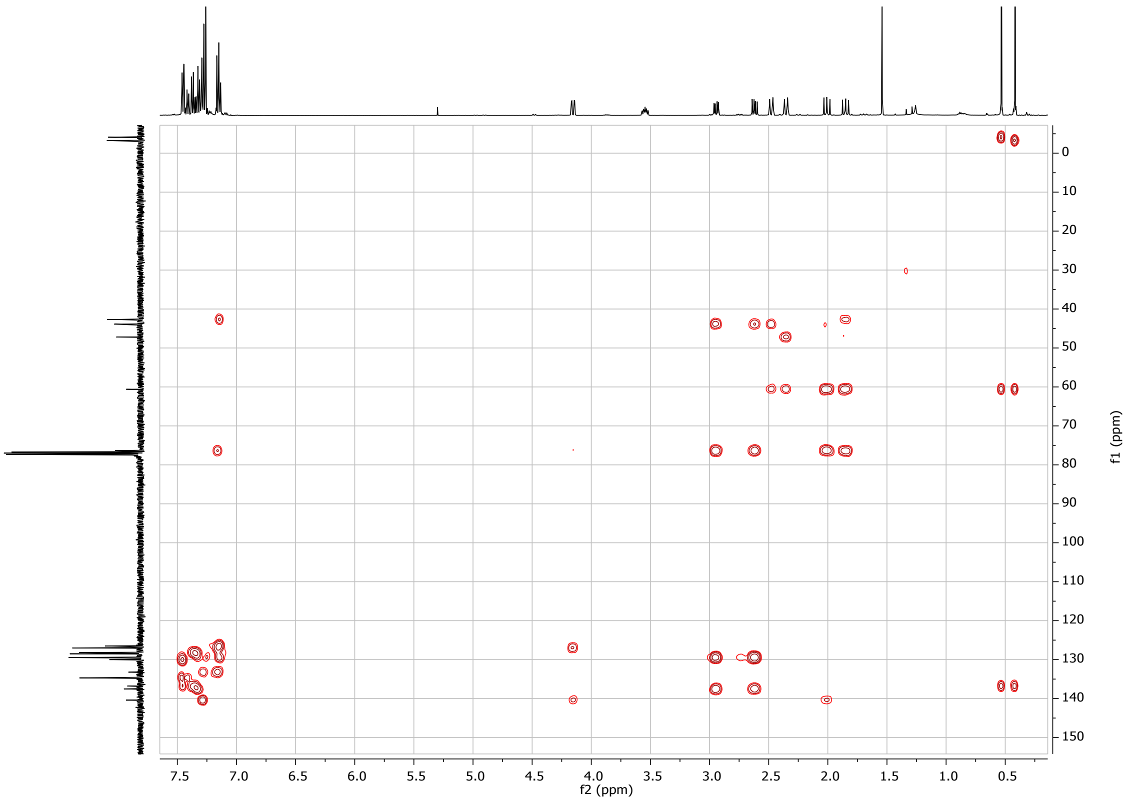

2D-NOESY of compound 2p

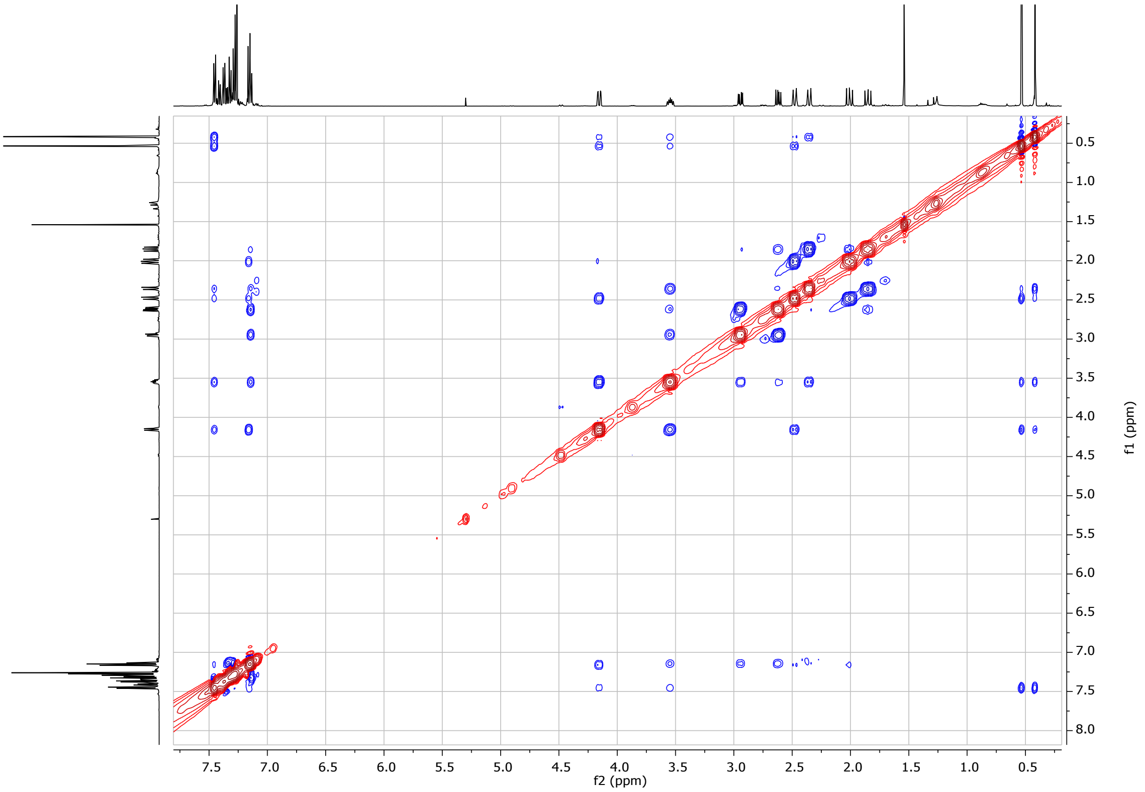

^{1}H NMR (400 MHz, $CDCl_3$)

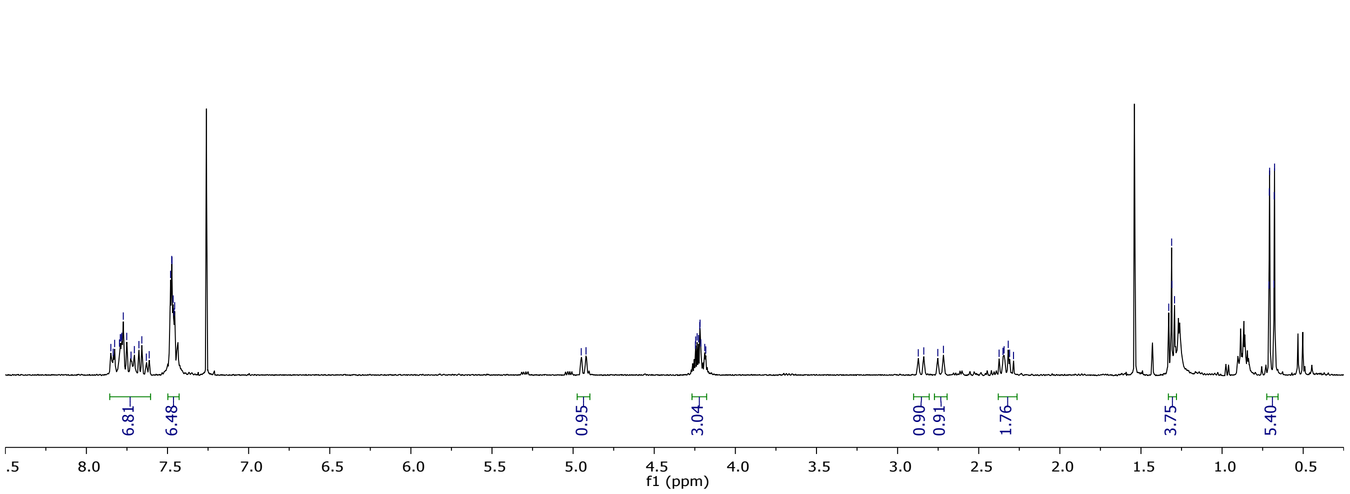

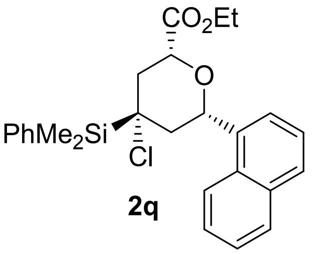

^{13}C {^{1}H} NMR (101 MHz, $CDCl_3$)

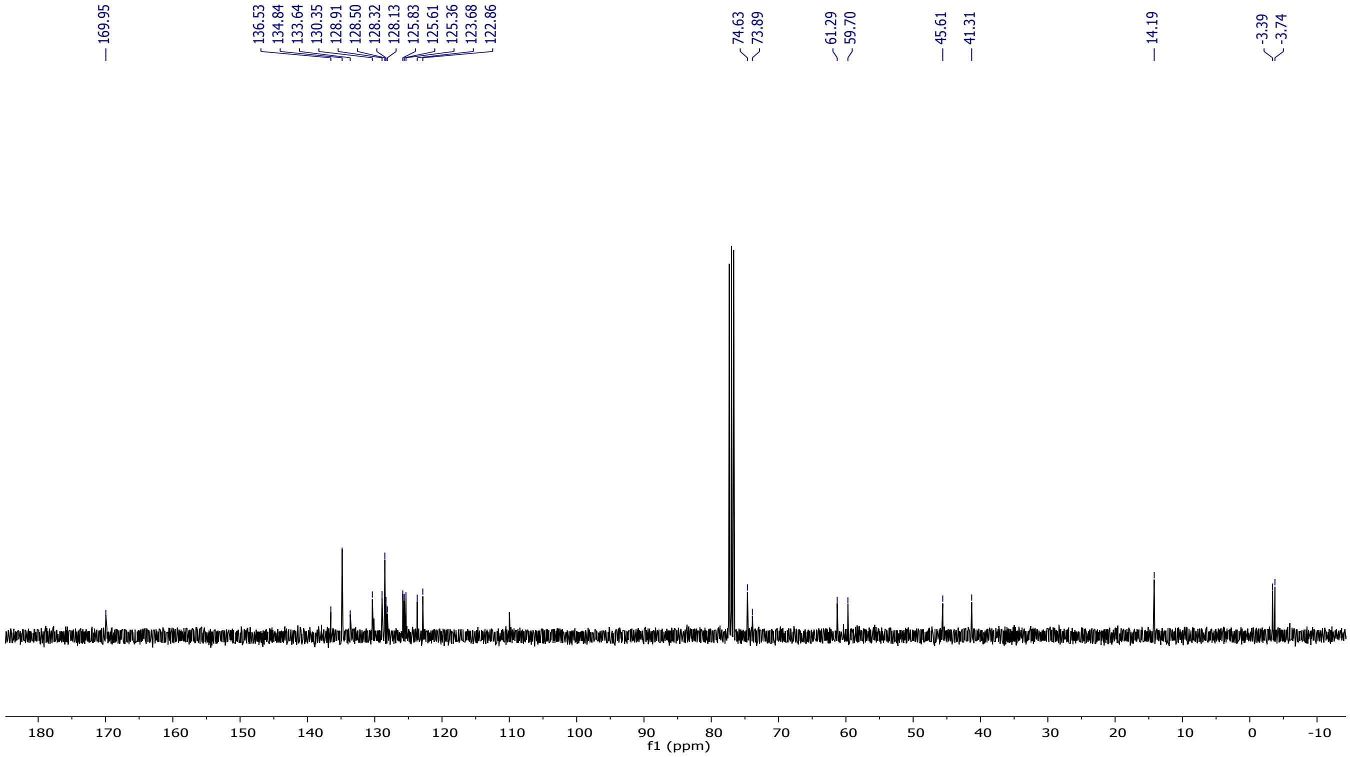

2D-COSY of compound 2q

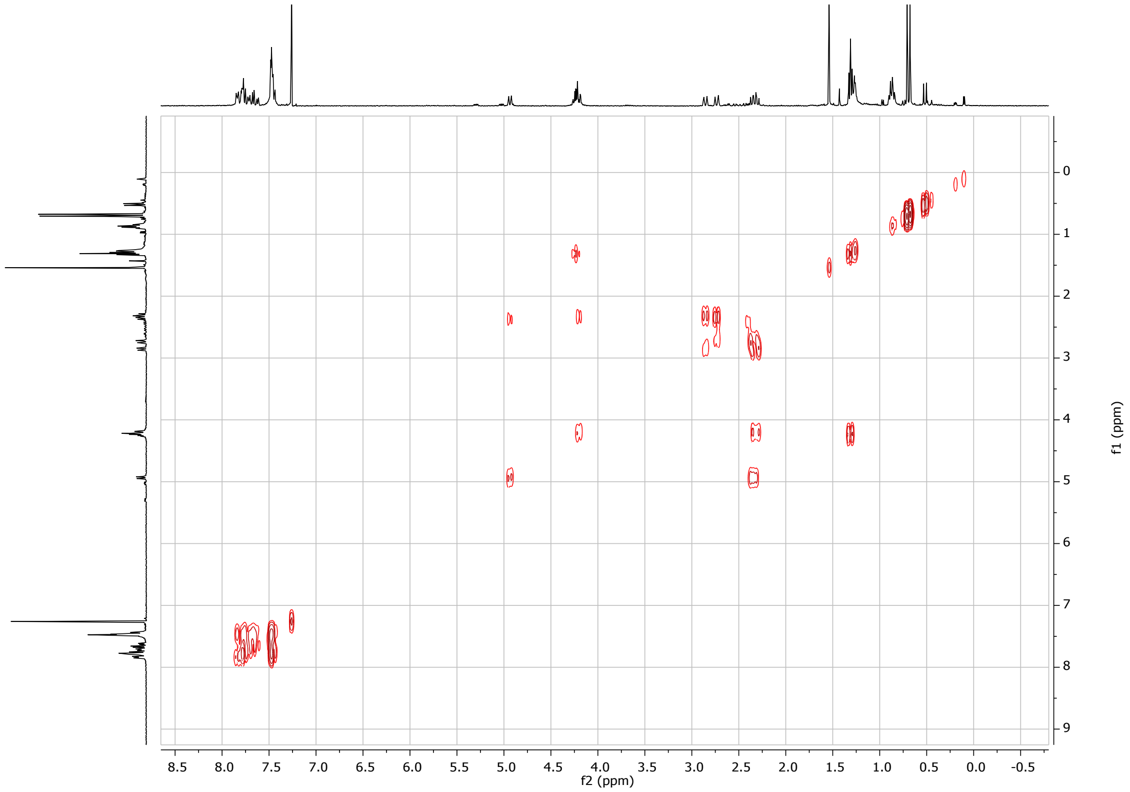

2D-NOESY of compound 2q

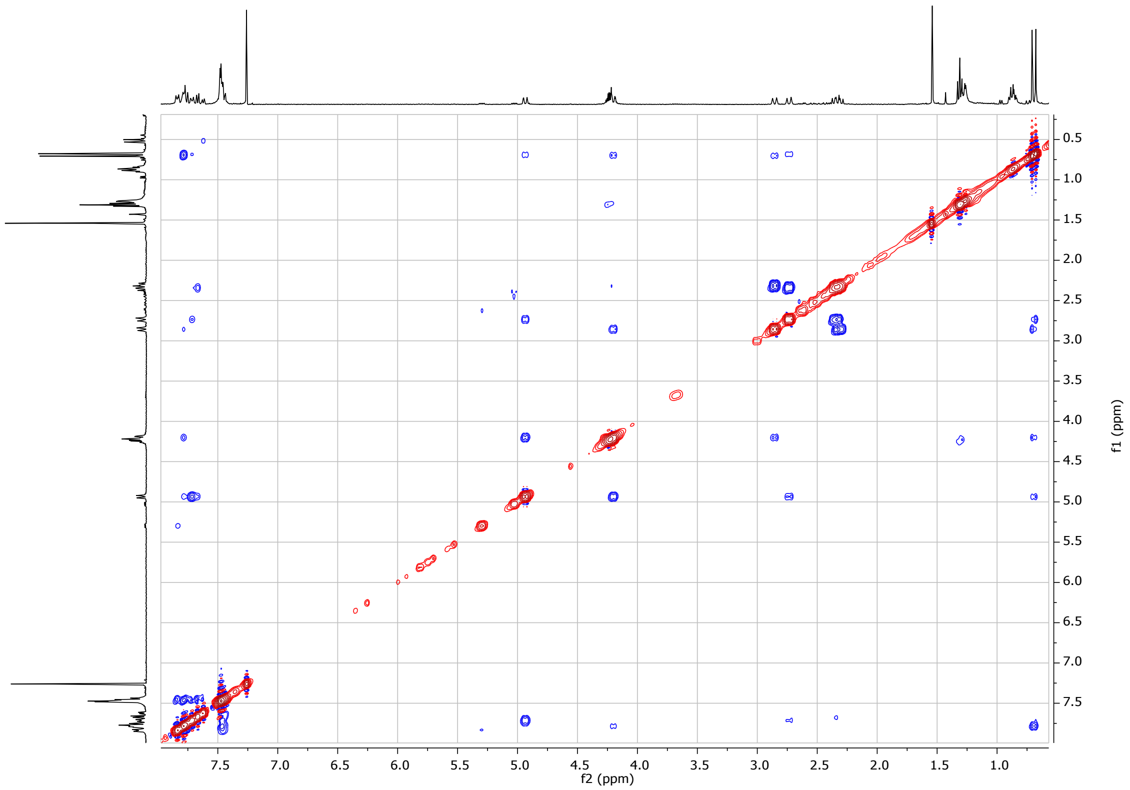

^{1}H NMR (400 MHz, $CDCl_3$)

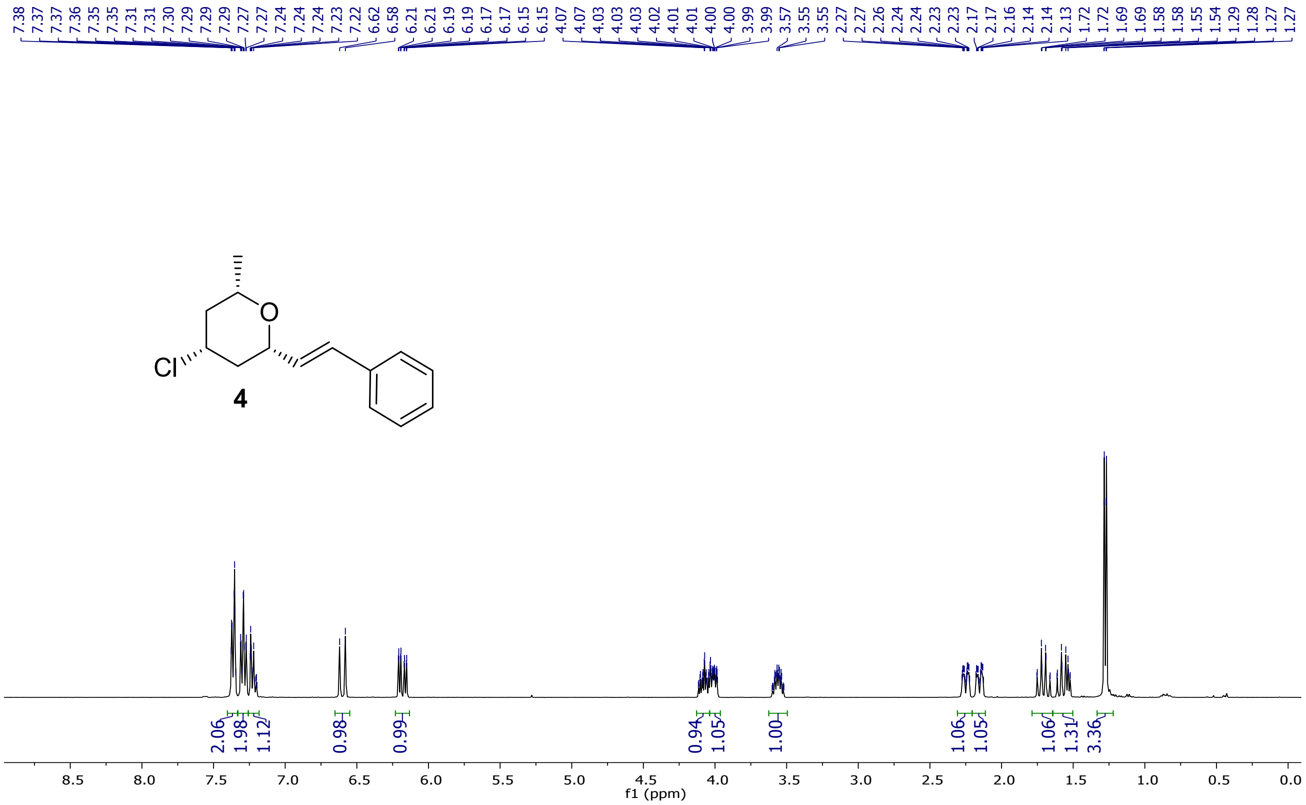

^{13}C {^{1}H} NMR (101 MHz, $CDCl_3$)

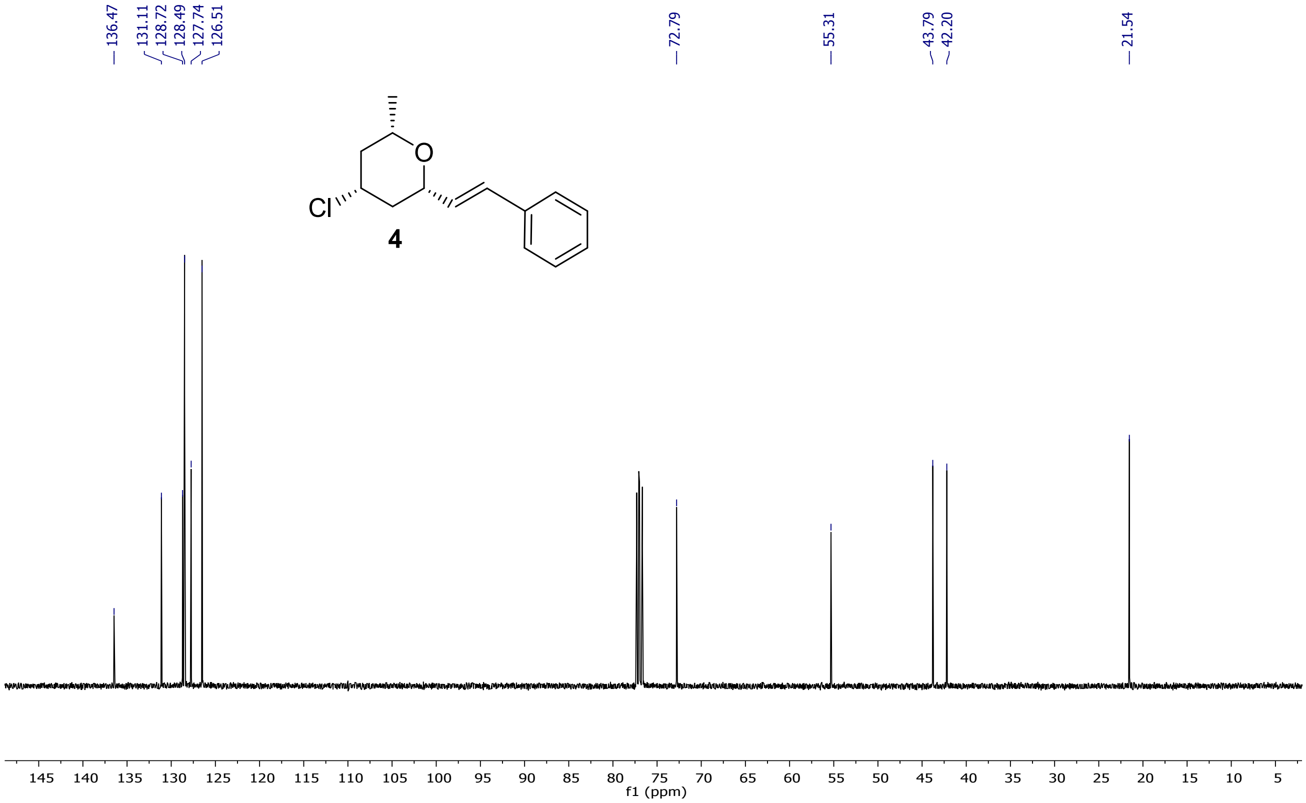

2D-COSY of compound 4

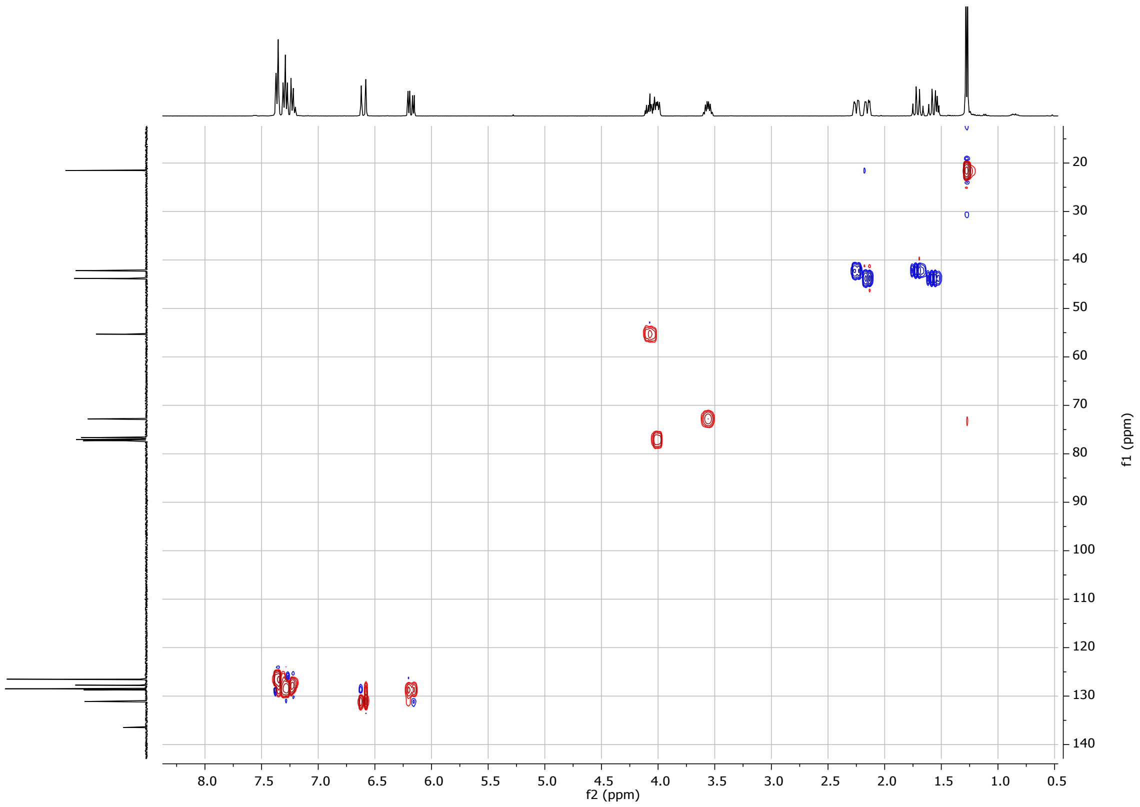

2D-NOESY of compound 4

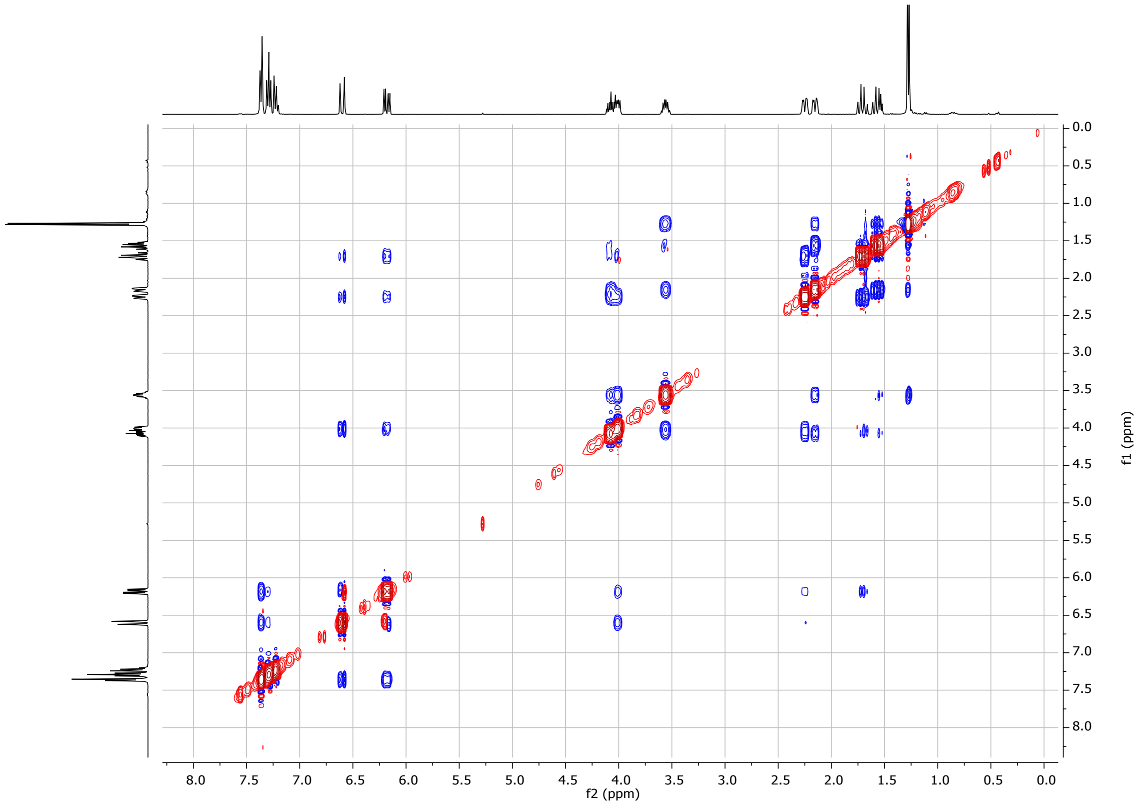

^1H NMR (400 MHz, $CDCl_3$)

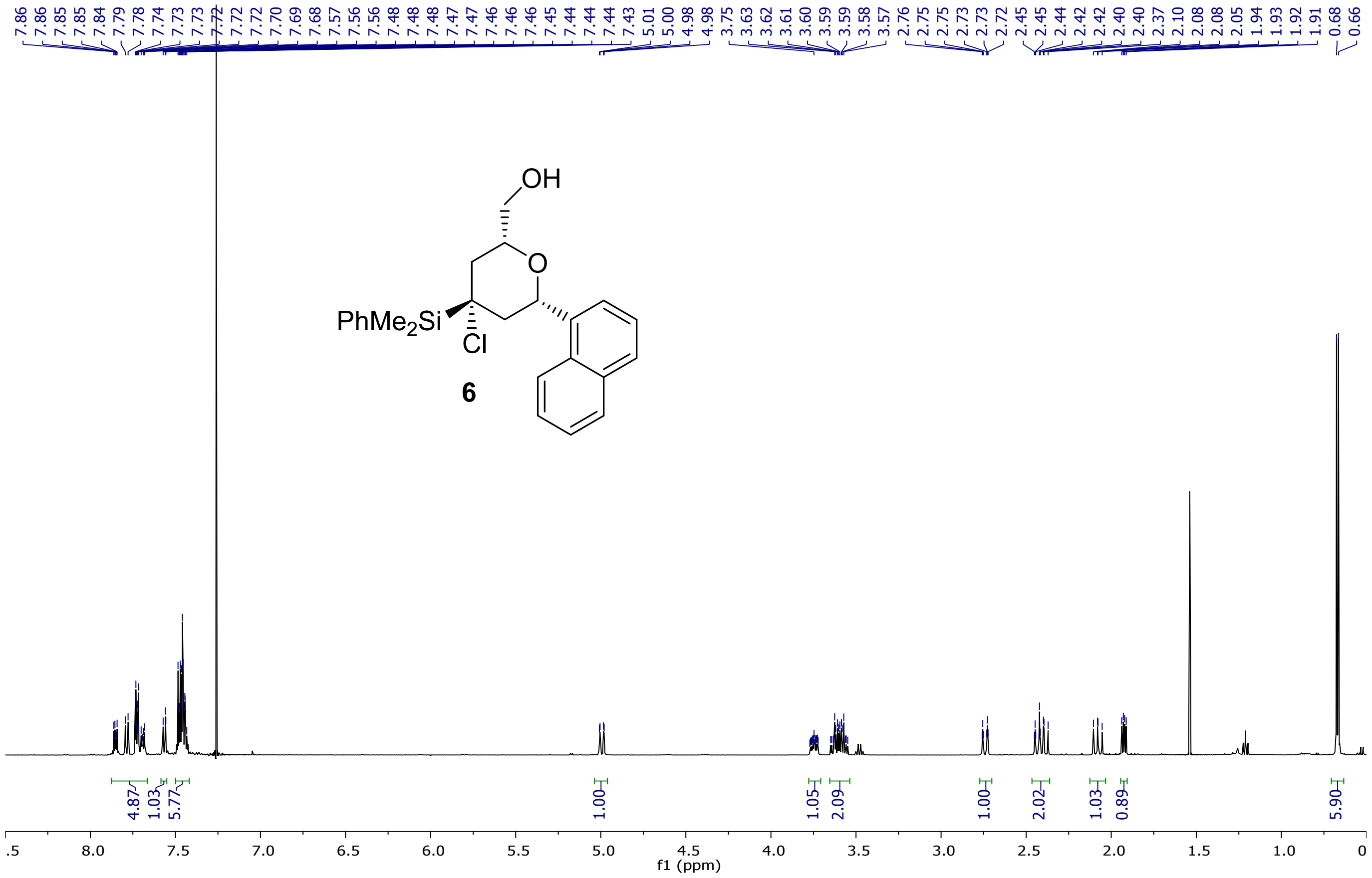

^{13}C {^1H} NMR (101 MHz, $CDCl_3$)

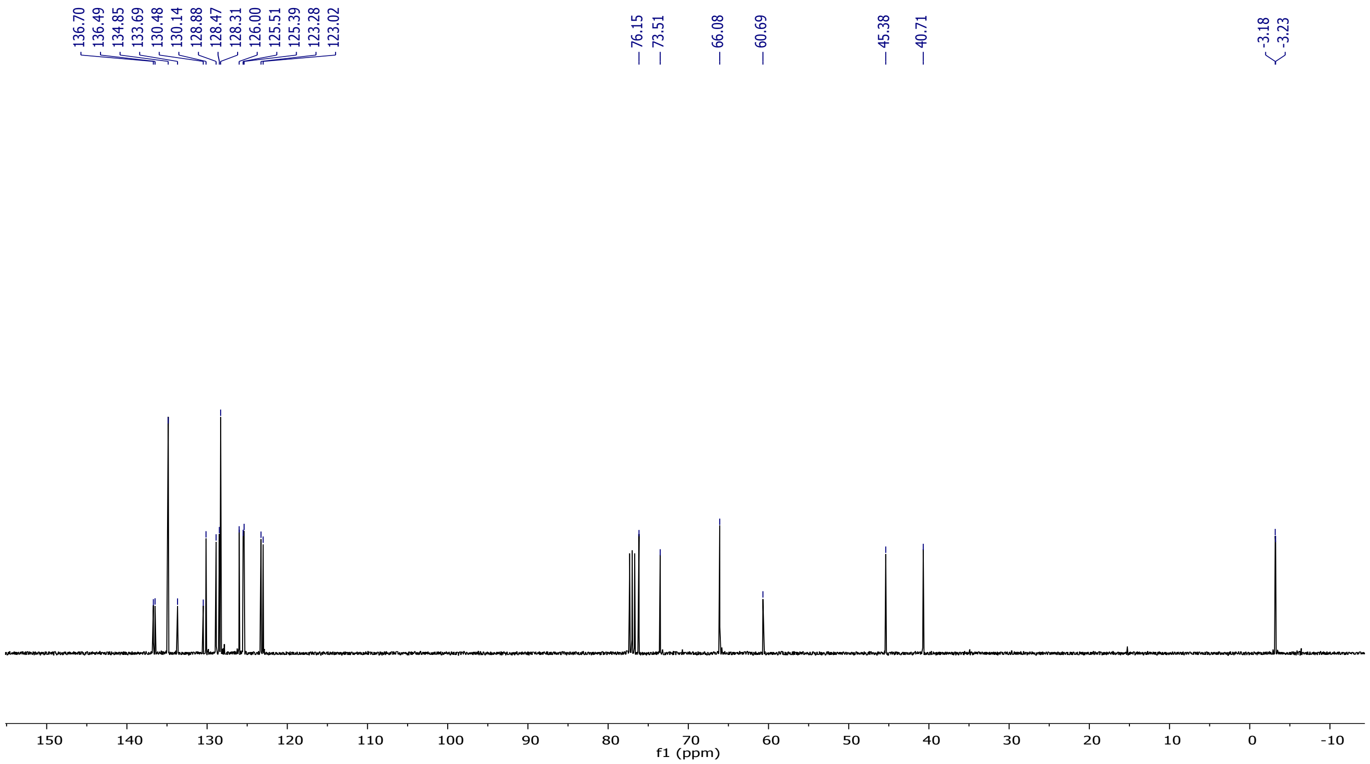

2D-COSY of compound 6

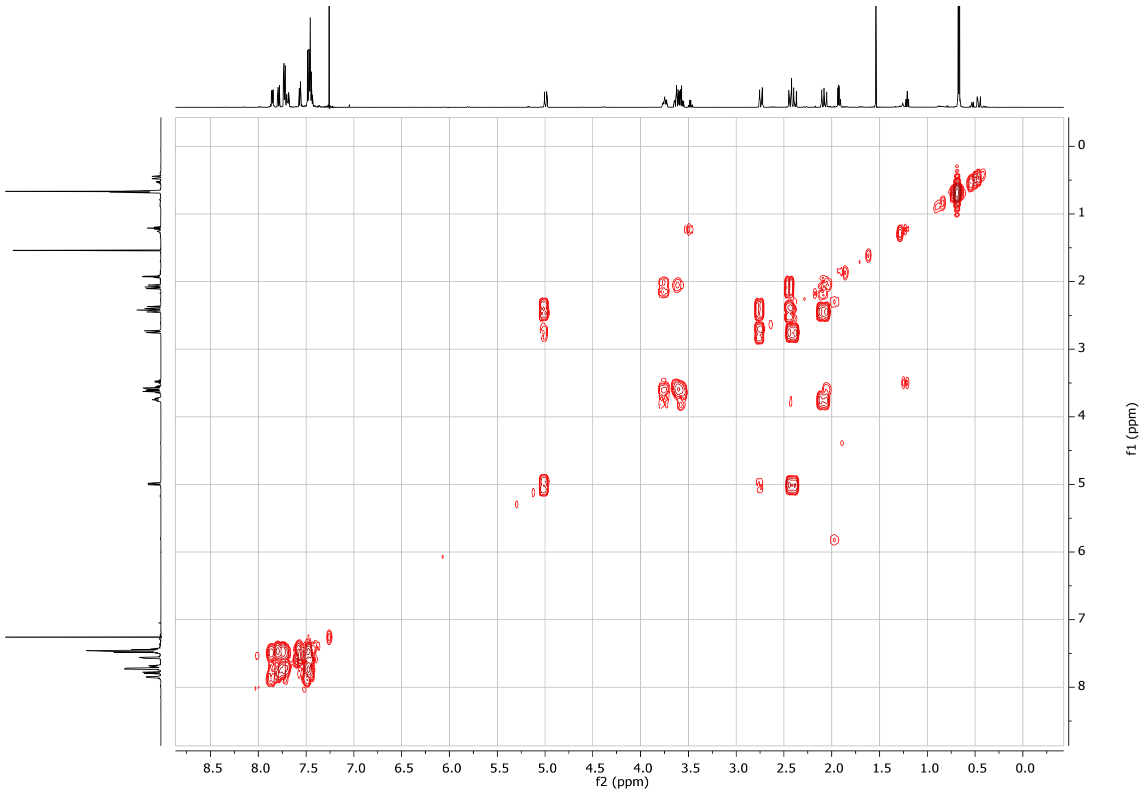

2D-NOESY of compound 6

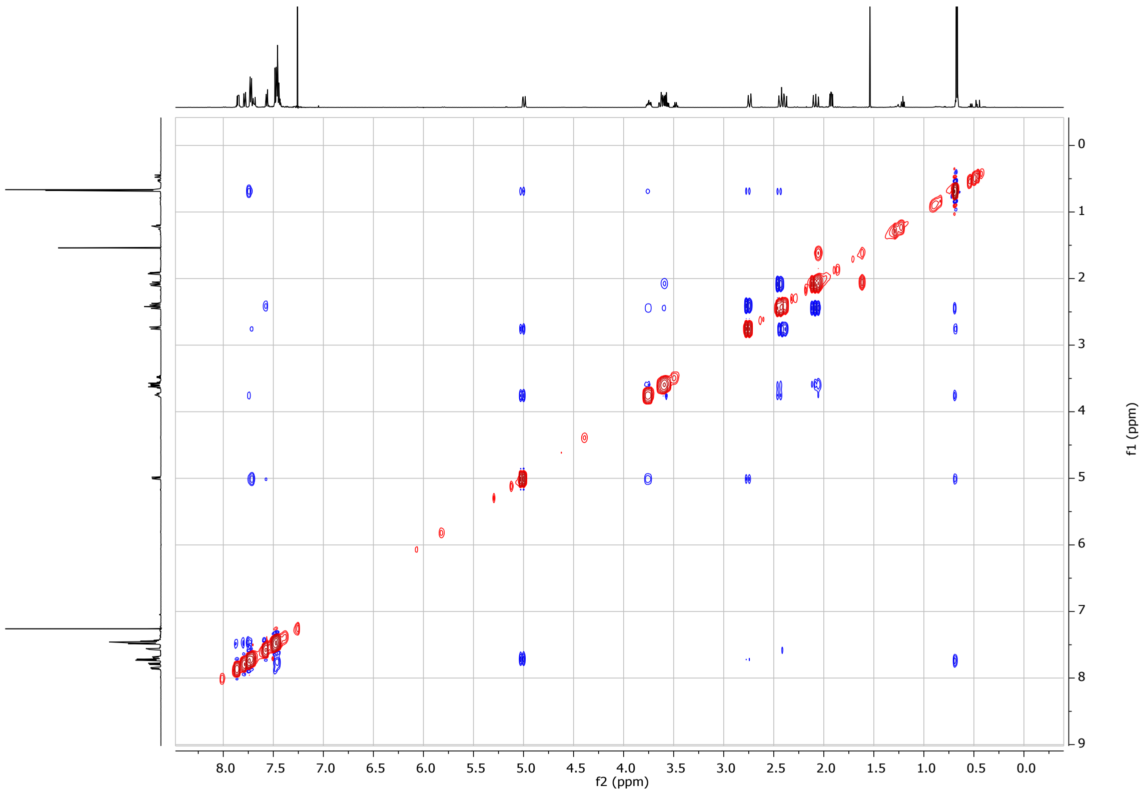

^{1}H NMR (400 MHz, $CDCl_3$)

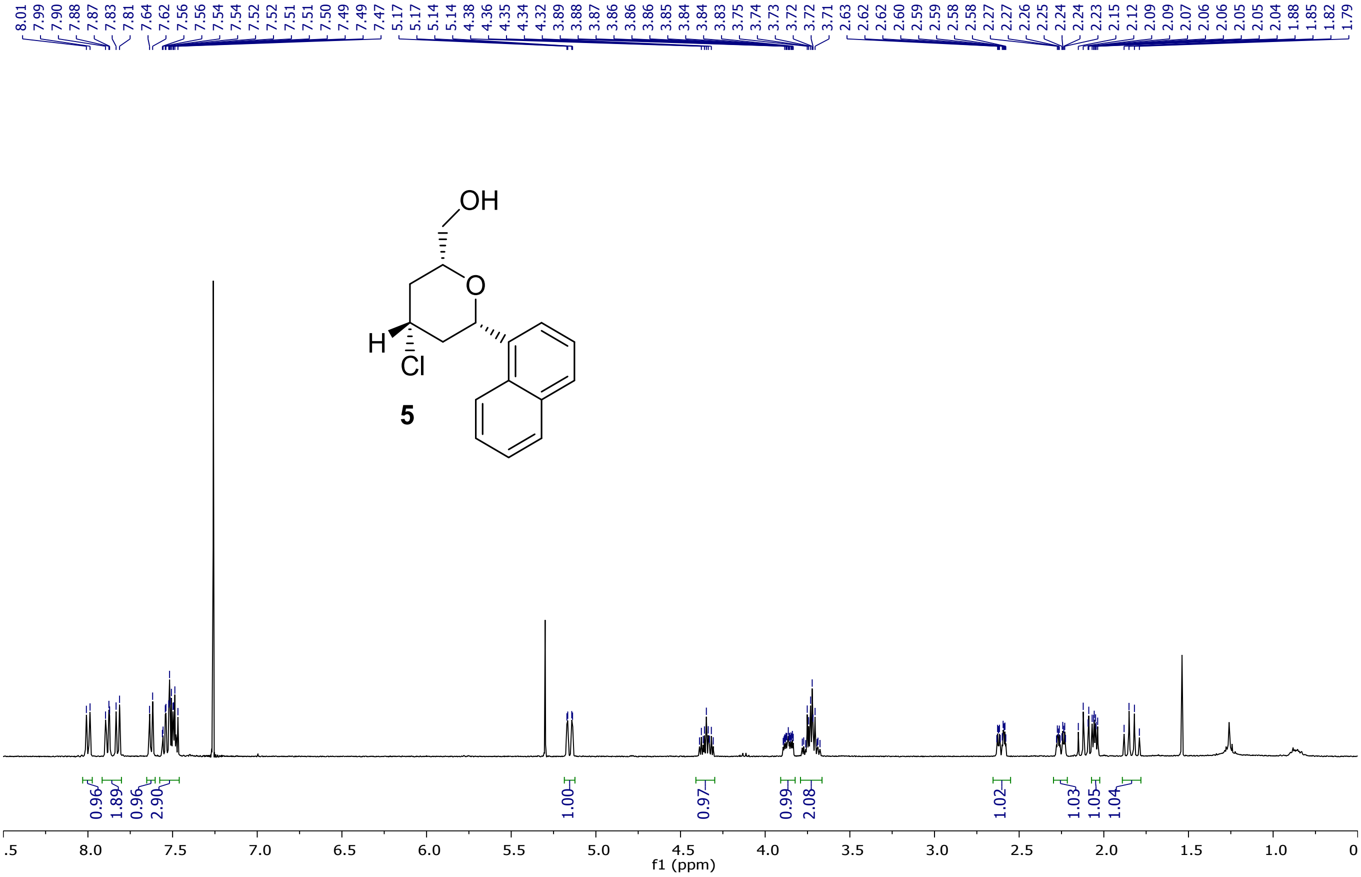

^{13}C {^{1}H} NMR (101 MHz, $CDCl_3$)

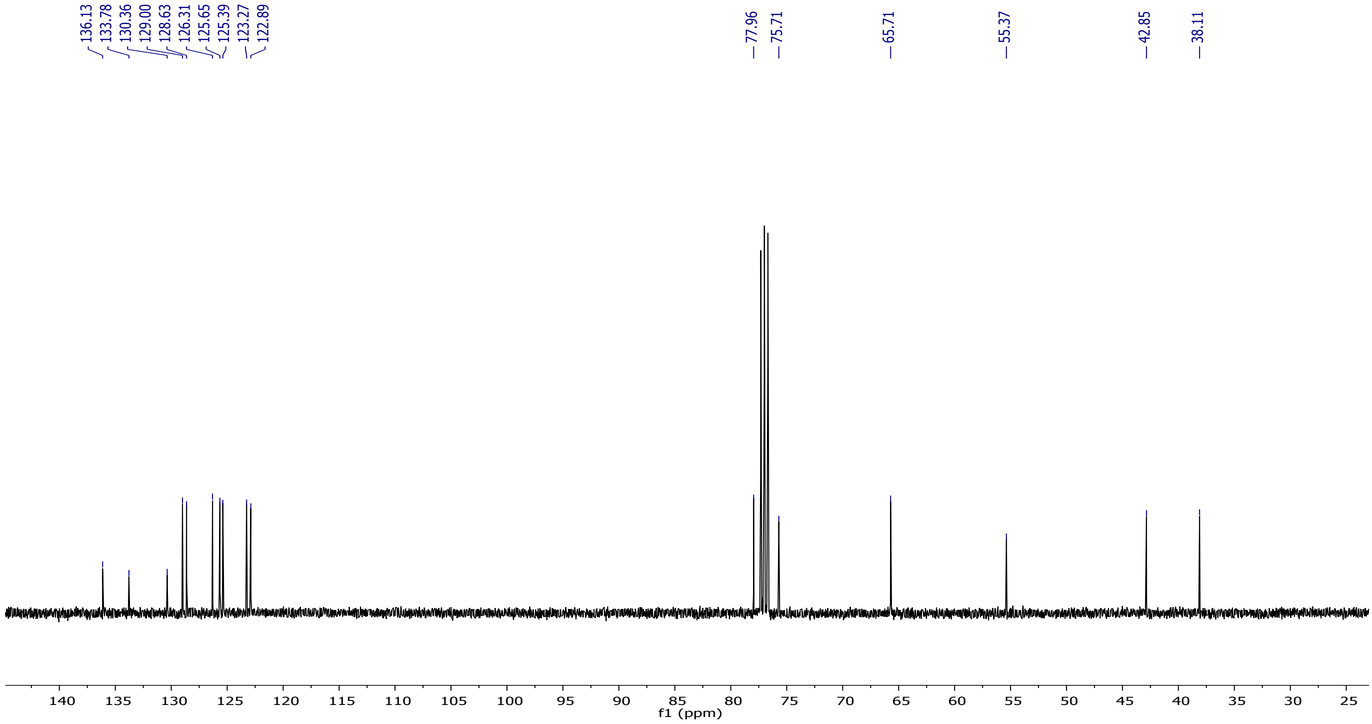

2D-COSY of compound 5

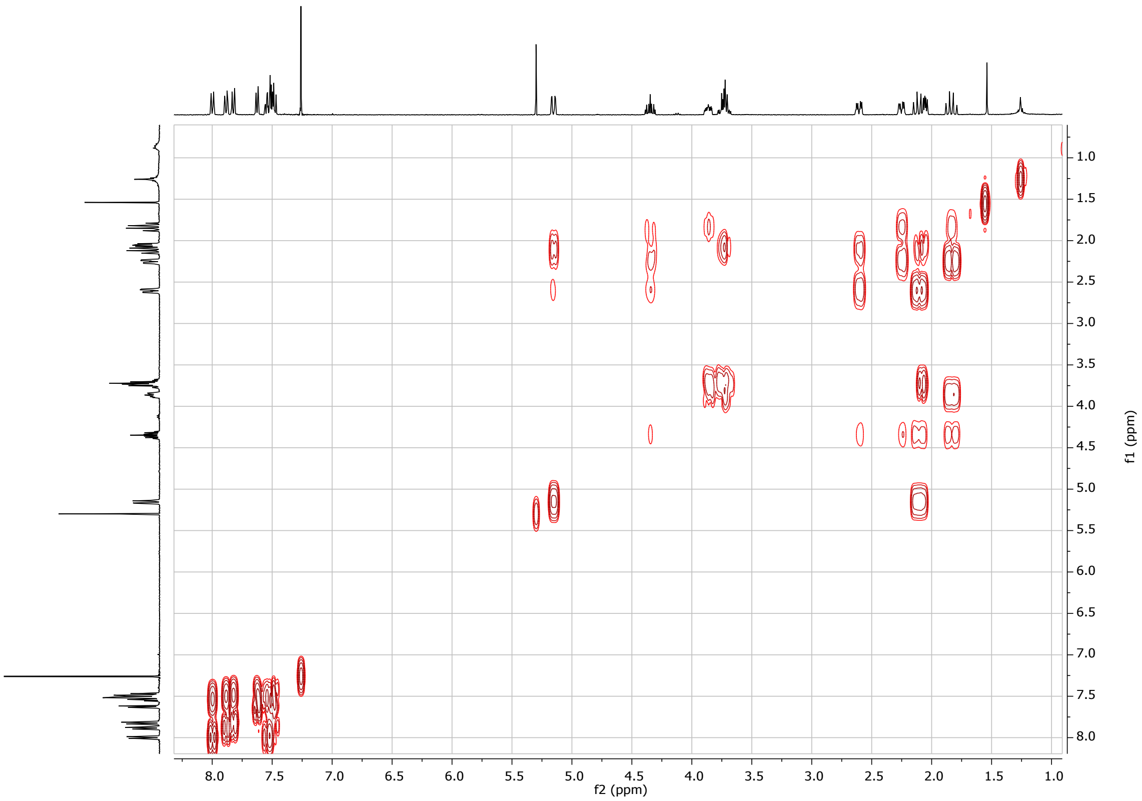

2D-NOESY of compound 5

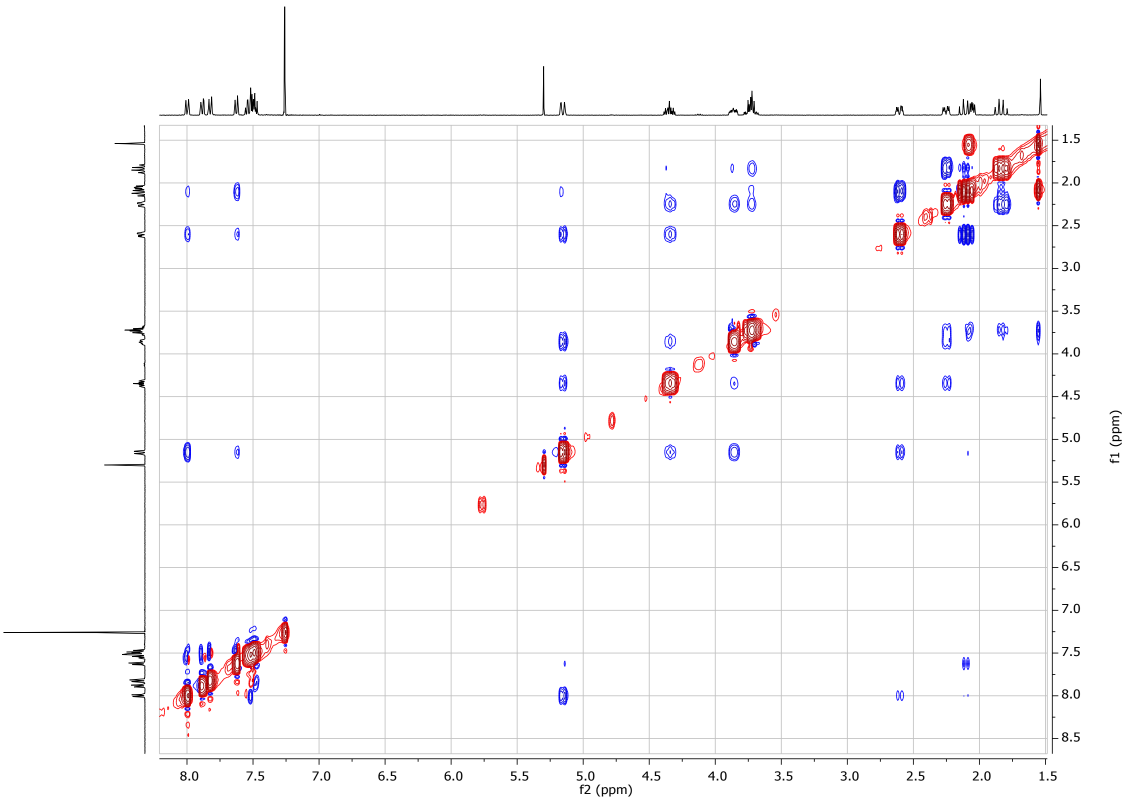

Supplement: Supplementary file 1 — jo3c00050_si_001.pdf [file jo3c00050_si_001.pdf]
